# Computational models of melanoma

**DOI:** 10.1186/s12976-020-00126-7

**Published:** 2020-05-14

**Authors:** Marco Albrecht, Philippe Lucarelli, Dagmar Kulms, Thomas Sauter

**Affiliations:** 1 Systems Biology Group, Life Science Research Unit, University of Luxembourg, 6, avenue du Swing, Belval, 4367 Luxembourg; 2 Experimental Dermatology, Department of Dermatology, Dresden University of Technology, Fetscherstraße 105, Dresden, 01307 Germany

**Keywords:** Melanoma, Systems biology, Physical oncology, Tumor growth

## Abstract

Genes, proteins, or cells influence each other and consequently create patterns, which can be increasingly better observed by experimental biology and medicine. Thereby, descriptive methods of statistics and bioinformatics sharpen and structure our perception. However, additionally considering the interconnectivity between biological elements promises a deeper and more coherent understanding of melanoma. For instance, integrative network-based tools and well-grounded inductive in silico research reveal disease mechanisms, stratify patients, and support treatment individualization. This review gives an overview of different modeling techniques beyond statistics, shows how different strategies align with the respective medical biology, and identifies possible areas of new computational melanoma research.

## Background

Melanoma is a neoplasm of the skin and originates from transformed melanocytes. It causes the loss of 1.6 million disease-adjusted life-years worldwide, and the incidence rate will increase in the next decades [1]. Since the discovery of the high prevalence of mutations in b-Raf proto-oncogene (BRAF) and NRAS protooncogene, GTPase (NRAS) [2, 3], small-molecule inhibitors such as dabrafenib and vemurafenib have been developed. More recently, immunotherapies, with antibodies binding immune receptors like the cytotoxic T-lymphocyte associated protein 4 (CTLA4) or the programmed cell death 1 (PDCD1), have proven to be clinically effective [4]. However, many drug resistance mechanisms occurred and represent a major problem in both targeted therapy and immunotherapy [5–7]. As a result, life expectancy remains low. The two-year survival rate is 53.5% for combined BRAF + mitogen-activated protein kinase kinases (MAP2K) inhibitors and 63% for combined CTLA4 + PDCD1 immunotherapy [8]. Consequently, a deeper understanding of disease mechanisms is still demanded. An approach to better understand causative relations, to check hypothesis consistency, but also to reveal missing qualitative information is constructing evidence-based models of these biological systems [9]. Models depict several interconnected biological elements with a structure, which is derived from the current understanding, and parameters, which are based on data. While many life-scientists still rely on straight-forward relationships between observation and insight to extend their knowledge, leading scientists report that the direct link between observation and insight seems to fade [10]. Thus, experimentally proven relationships are increasingly transferred into the language of mathematics to enhance our understanding of experimental findings and underlying reasons.

Cancer scientists can benefit from well-designed computational models, whereby systems biologists deliver models of cancer biochemistry, and physical oncologists provide models of tissues. Systems biology helps understanding how biochemical pathways change during melanoma cell proliferation, invasiveness, survival, and drug resistance based on network structure and dynamic behavior [11]. By contrast, physical oncology helps understanding how transport, growth, and deformations in tissues occur and is characterized by principles of geometry and mechanics [12, 13].

In this review, we tried to collect all published computational models of melanoma and describe them regarding their contribution to the field. In particular, we focus on the interconnection of system elements or network characteristics while omitting classical statistics and bioinformatics of melanoma. By sorting models and methods around the topic of melanoma, we intend to support readers in finding the most appropriate mathematical model to address their melanoma-specific research questions. Additionally, the review shall describe potentials for improvement, encourage readers to discover potential extensions, and create awareness of missing melanoma topics to be tackled in the next decade. However, even if some models seem simplistic in biology, they often represent technically challenging stepping-stones for more biologically meaningful models in the future. Consequently, reviewing the currently existing models might help to push forward the modeling and computational characterization of melanoma.

**Fig. 1:**
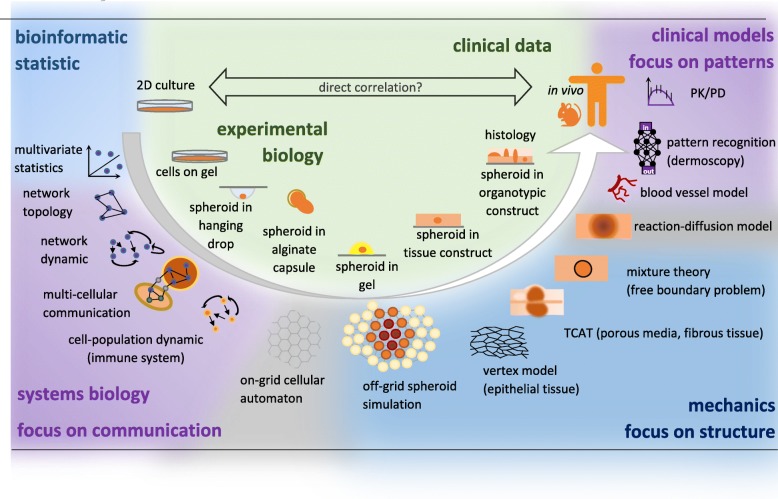
**Computational and experimental approaches to understand cancer**. **Experimental approaches** span from 2D cell culture to clinical data and are often correlated directly. Possible intermediate steps can delineate the response of cells to certain characteristics of the environment. Cells on gel sense the rigidity of the substratum, spheroids in hanging drops can develop a necrotic core, spheroid growing in alginate capsules reveal the growth pressure at which the capsule burst, spheroids in gel reveal the cellular response to a confined environment, spheroids in a tissue construct shows interactions with fibroblasts and host cells in a confined environment, and organotypic constructs and histological sections emphasize the behavior in a realistic anatomical structure. **Computational models** change accordingly in scale and approach. Methods are classified counter-clockwise, beginning at the top left corner. *Descriptive methods of statistics and bioinformatics* focus on the identification of single features. Often groups are compared, or the explanatory power of certain factors is investigated. *Systems biologists* increasingly connect different elements, focus on network information, and study dynamic effects. The network topology in steady-state is the first step but can also be extended to time dynamic and directed interactions. The networks might be compartmentalized to study communication across different cells, but the cells themselves can also represent network nodes, which is common in immunological studies. If interconnections between cells, with or without ECM, are studied and spatially distributed, on-grid and off-grid cellular automatons, vertex models, and reaction-diffusion models become relevant. Deformed tissue structures and anatomical obstacles require the integration of mechanical information. The more the approaches move from cell data to clinical images, the more pattern recognition becomes relevant. The functioning of the blood vessel system often depends on the pattern of the vessel network. Clinical images, such as from dermoscopy, might be linked via artificial intelligence to various pathologies. At the top right, computational methods of pharmacokinetics and pharmacodynamics relate drug dose to the concentration in blood plasma and then to the mode of action. The upper half of the figure pronounce the statistical significance; the bottom half of the figure shows models, which pronounce the importance of physical and mechanistic dependencies. In conclusion, a direct correlation between in vitro and in vivo data might be straight-forward, but might be also too simplistic. The laborious indirect way with step-wise experimental and computational extension of knowledge might be harder and more expensive, but more insightful in the long term and can enrich meaningful model development

The review is structured as follows: Network-based approaches are explained in “Molecular networks” and complemented by melanoma-specific repositories. The complex interaction between molecular players requires network-based approaches to suggest novel key intervention strategies, to stratify patients, and to individualize patient treatment. In “Cell population models: bridging cell culture to clinics”, the dynamic changes in cell count of different melanoma cell types, immune cells, and fibroblasts are modeled and complemented by stimulating or inhibiting effects between cells. Such cellular models represent another way to achieve therapy individualization and patient stratification. “Spatial models of melanoma” leads to geometric effects which will be augmented by the mechanics of melanoma in “Mechanical models of melanoma”. Further aspects of oxygen, nutrient, and drug transport are presented in “Transport of oxygen and drugs” sections. The confined, spatial, and physiological tissue environment is relevant for tumor growth prognosis, drug delivery, surgery, and dermoscopic pattern recognition. All available computational melanoma models are listed in Supplemental Table 1 and summarized in Fig. 1.

## Molecular networks

Molecular networks represent larger sets of molecules in an interconnected manner and go beyond the statistical significance of single features and the gene-set enrichment analysis paradigm [14]. Network science shows how biological functions emerge from the interactions between the components of living systems and how these emergent properties enable and constrain the behavior of those components [9]. In order to explore this rich information source, system biology provides frameworks tailored to each commonly known -omics data type. Melanoma-specific -omics data can be obtained from genomic [15, 16] and proteomic studies [17] but also from the secretome [18] and the metabolome, respectively [19, 20]. Because multiple -omics data are rarely integrated with a systems-centered approach [21], the following studies and repositories are only a starting point.

### Repositories to inform network models

Published knowledge in the form of structured and centralized databases facilitates model development. Beside general sources for system biologists [22], melanoma-specific databases are available (Table 1). The Melanoma Molecular Map Project (MMMP) is an open-access, participative project that structures published knowledge about molecules, genes, and pathways to enable translational perspectives [23]. The MelGene project provides an easily searchable database of genetic association studies of cutaneous melanoma, as well as a meta-analysis for many polymorphisms [24]. The MelanomaDB database lists published genomic datasets, including clinical and molecular information, and allows the creation of gene lists by merging selected studies [25]. The Melanoma Gene Database (MGDB) provides extensive entries about 527 melanoma-associated genes (422 protein-coding), including epigenetic and drug-related evidence [26]. Caution is required when using these databases, which accumulate data from multiple sources, sometimes in an automated manner, and are therefore susceptible to perpetuate the biases and errors of the data source [27].

**Table 1:** Data bases containing melanoma data

Databases	Information	Last update	Source
Melanoma Molecular Map	Information about single molecules molecular	2015	[23]
Project	profiles and molecular pathways involved in		
	melanoma progression		
MelGene	83,343 CM cases and 187,809 controls and reported	2016	[24, 174]
	on 1,114 polymorphisms in 280 different genes		
MelanomaDB	Published melanoma genomic datasets	20 May 2013	[25]
	including clinical and molecular information		
Melanoma Gene Database	Relationship between melanoma protein-coding	02 Nov 2016	[26]
	genes, microRNAs and lncRNAs		

### Models of melanoma genomics

The melanoma-specific repositories contain mainly genetic data with not yet fully identified patterns. The mutation pattern within the genome of metastatic melanoma can be used to find mutually exclusive gene modules [28]. If two proteins are related in an interaction network and their genes are mutated in a way that one gets amplified while the other gets deleted or only one gets modified without the other, one could presume that this happens to intensify cancer pathways at the protein level under given pathophysiological pressure. Consequently, one can conclude that a protein inhibits or activates the other in a known interaction network. The pathophysiologic pressure on cancer protein pathways selects mutation patterns with survival benefits. One analysis of The Cancer Genome Atlas (TCGA) melanoma samples integrated somatic mutations with copy number alterations and found concomitant deregulation of the G-protein and MAPK signaling pathways [29]. Similarly, integrated genomic and epigenomic analyses have been used to classify melanoma brain metastases in different mutually exclusive molecular subtypes [30].

### Models of melanoma transcriptomics

The melanoma transcriptome is more context-specific than the genome and easier to measure than the proteome. The pattern changes can be used to stratify patients or to identify drug targets. Beyond this, they can give an impression of the re-wiring of pathways. Barter et al. applied three different strategies (single genes, gene sets, and network analysis) to 47 melanoma microarray datasets. They concluded, that network methods do not perform better overall, that these different approaches tend not to classify patients consistently, and that the optimal method might have to be identified patient-specifically [31]. Wang et al. performed 45 siRNA screens of the melanoma cell line A375, whole-genome sequencing, and Bayesian gene network interference to enable directional and synergistic conclusions. Similar to Barter’s findings, the network hubs alone were not sufficient to better stratify patients. However, if the network hubs are contextualized with cell-cycle and deoxyribonucleic acid (DNA)-repair function, the prediction of an individual prognosis was shown to be possible [32].

The concept of pathway re-wiring is based on the following reasoning. Some mutations can cause protein structure modifications, which in turn can alter the linkage between proteins without changing cellular protein levels. Two proteins only interact if the transcript level change of one protein correlates or anti-correlates with the transcript level change of another protein. If co-expression is abrogated, the network connectivity reduces. When two unrelated proteins show a new co-expression in the next progression stage, the connectivity increases, and a pathway re-wiring can be assumed. This network analysis can be performed independent of significantly changed differential expression and fold changes.

Kaushik et al. followed this strategy and meta-analyzed 632 melanoma microarray samples with melanoma progression stages: normal skin, non-metastatic (radial and vertical growth phase), metastatic, and lymph node metastases [33]. They diversified the clinical relevant groups by pooling the data of tissue samples with untreated and cisplatin-treated melanoma cell lines and melanocytes. The extracted re-wired pathway hubs were subsequently checked for drugability, which is important as many promising targets cannot be influenced pharmacologically [34].

### Models of melanoma proteomics

The proteome directly mirrors cellular function. Genomic and transcriptomic data can only indirectly show the post-transcriptional, translational, and further epigenetic changes and are thus limited in their representation of final physical processes. Proteomic data are, e.g., very beneficial for modeling signal transduction pathways such as the MAPK or phosphatidylinositol-4,5-bisphosphate 3-kinase catalytic subunit alpha (PIK3CA) pathway [35]. In the context of melanoma, most studies aim either at understanding resistance mechanisms or at the response rates to particular compounds. For example, it was possible to predict the degree of apoptosis for 11 melanoma cell lines treated with TRAIL and dacarbazine (DTIC) with high accuracy. This was achieved by grouping measurements in pathway-inspired functional groups and using these in multivariate statistical analysis [36]. Resistance in melanoma cell lines was studied with data-driven modeling and multivariate statistics. 21 phosphoproteins were measured over time in a panel of 10 cell lines subjected to different doses of five different RAF/MAP2K inhibitors [37]. This led to the identification of an early down-regulation of the mitogen-activated protein kinase 8 (MAPK8)^1^/jun proto-oncogene, AP-1 transcription factor subunit (JUN) pathway upon RAF/MAP2K inhibition, but an up-regulation in six cell lines at later time points. This study showed that a fraction of treated cells become quiescent and apoptosis-resistant. The same group further validated these results and suggested targeting MAPK8, protein tyrosine kinase 2 (PTK2), or SRC proto-oncogene, nonreceptor tyrosine kinase (SRC) to inhibit this particular drug-resistant phenotype [38]. Bernardo-Faura et al. used Fuzzy Logic to investigate the temporal network re-wiring in A375 cells in response to different kinase inhibitors [39]. The authors used a prior-knowledge network to simulate the behavior of the cells over time, and detected discrepancies at specific time-points between the model predictions and the measurements. This work, as well, underlines the importance of the MAPK8 pathway in early drug-induced changes in signaling pathways. Del Mistro et al. studied the signaling network changes in phospho-proteomic data due to underlying resistance of BRAF mutated melanoma cell lines to sublethal doses of tumor necrosis factor related apoptosis inducing ligand (TRAIL) receptor-targeted agonist IZI1551. Systemic network analysis with Dynamic Bayesian modeling identified X-linked inhibitor of apoptosis (XIAP) and NF- *κ*B inhibitor alpha (I *κ*B *α*) as potential drug targets. Consequently, targeting these nodes in the subsequent experimental validation led to a sensitization of cells [40]. Another comprehensive study contained 89 perturbation conditions and 143 proteomic/phenotypic measurements with the result that bHLH transcription factor (c-Myc) might be a potential therapeutic co-target in addition to BRAF or MEK inhibition [41].

### Models of melanoma metabolomics

The metabolic state is the consequence of proteomic function and environmental conditions such as nutrient and oxygen shortages. Metabolite concentrations can be obtained with robust measurements, and well-established methods are available [42]. Notably, Scott et al. used metabolic flux analysis to characterize the response of seven melanoma cell lines to hypoxia [43]. They showed the crucial roles of both Warburg and Pasteur effects in melanoma and paved the way for the therapeutic targeting of metabolism. While the Pasteur effect describes reduced glycolysis with increased oxygen, the Warburg effect refers to cancer cells performing glycolysis despite the presence of oxygen [44]. Future studies might further combine metabolic modeling with other omics-data.

### Mechanistic network models of melanoma

Completely validated mechanistic network models of melanoma have not been published yet, but a valid Boolean model of melanogenesis combines both keratinocyte and melanocyte signaling without cancer properties. Lee et al., thereby, imposed increasing ultraviolet B (UVB) light intensity and modeled the cellular response to it. The simulated profiles of the protein levels were individually compared to literature to check qualitative plausibility. Lee et al. demonstrated the central role of catenin beta 1 (CTNNB1) in the regulation of both melanogenesis and apoptosis. This prediction was then validated using UVB-exposed reconstituted human skin equivalents [45].

Moreover, a system of ordinary differential equations (ODE) was used to model the MAPK, PIK3CA/AKT serine/threonine kinase 1 (AKT1), and other pathways with 48 species and 48 biochemical reactions [46]. The model was an extension of the model of PC-12 (rat adrenal gland) cells [47] and showed that increasing dabrafenib concentrations cause declining pERK levels but in unphysiological ranges. Future ODE-based modeling of melanoma signaling would ideally improve the balance between model size and melanoma-specific data to enable robust predictions. Sensitivity analyses and a model selection procedure might help to suggest key mechanisms and intervention strategies.

As described in this section, the network information can be used to stratify patients, to find druggable targets, and to understand the impact of therapy on the biochemical pathways. The next section describes models to inter-connect cells instead of molecules. Cell population models are used to find coherencies between cell culture and clinical patient populations or to understand the immune system at the whole-body level.

## Cell population models: bridging cell culture to clinics

Melanoma cells are not isolated entities but interact with keratinocytes, fibroblasts, and immune cells. Moreover, melanoma cells might be divided into subtypes or phenotypes. Population models often describe the interaction between them, e.g., how the level of one cell population influences the abundance of another cell population. A subset of these models integrate cell culture data; another subset of these models are experimentally adjusted with human or murine in vivo data.

### Melanoma models can mimic the interplay of cell types

Flach et al. studied the interplay of melanoma cells, stromal fibroblasts, and stromal fibronectin. In their interpretation, free melanoma cells at the lesion border activate fibroblasts to get mechanical support. The mechanically supported cells proliferate until they become blocked due to space limitations, albeit the space limitation is simplified to state values in this ODE network model [48]. Accordingly, several studies point to the crucial role of remodeling, fibronectin, and PTK2 signaling in driving resistance to BRAF inhibitors [49, 50]. This conceptual model of Flach et al. has been refined, validated, and extended to BRAFi and PTK2i therapy [51]. The results allowed a deeper understanding of the role of stroma during acquired resistance and its potential role during targeted therapy in drug-resistant patients [48, 51]. The same group worked on a dynamic autophagy model with AKT1i therapy for melanoma [52]. Integration of cell culture and clinical patient data into the autophagy model enabled the identification of key stratification parameters. Stratification parameters could either accompany clinical trials or might support treatment selection in the future. Another melanoma cell population model is provided by Sun et al. with an excellent description of the parameter origin. The considered cell types are BRAFi sensitive, BRAFi resistant, and may or may not enter the metastatic state after drug treatment. Cells grow until a maximum cell burden. The set of stochastic differential equations with 19 parameters is experimentally adjusted via circulating tumor cell DNA and melanoma cell line data. Progression-free survival is set equal with the melanoma cell concentration for simplicity [53], whereby more data might allow a more clinical relevant linkage between these two. Future models with integrated pharmacokinetic elements might consider clinically relevant pharmacokinetic models [54].

### Cell interplay is studied for melanoma immunology

Cell population models of the interplay of melanoma cells with immune cells are helpful as melanoma is a highly immunogenic tumor [55]. This high immunogenicity is the reason for the success of therapies based on immune activation in this tumor type. Indeed, melanoma was the first cancer type for which an immune checkpoint inhibitor and an oncolytic virus were approved [56, 57]. As such, several computational models have been specially developed to study the interplay between immune and melanoma cells. For example, several ODE systems were devised to model melanoma with Th1 and Th2 helper lymphocytes [58], with natural killer cells (NK) in the context of interleukin 21 (IL21) therapy [59], with M1 and M2 macrophages [60], or both macrophages and helper lymphocytes [61]. Also, vaccine strategies based on dendritic cell therapy for melanoma were modeled with a multi-compartment ODE system to define adequate doses and schedules [62]. However, one drawback of these models is that the patients’ intrinsic variables, key determinants in immune-related therapies, were not taken into account [63]. One study took into account the genetic signatures being associated with resistance to immunotherapies. The parameterized ODE model suggested co-adjuvants for successful vaccine therapies [64]. In another study, Pappalardo et al. implemented an on-grid cellular automaton model of melanoma, in which melanoma cells interact with macrophages, T cells, and dendritic cells at different cellular states. Pappalardo et al. highlighted the role of TNF receptor superfamily member 9 (TNFRSF9) for successful therapy and adjusted their model with experimental data of activated or resting OT1 T-cells and anti-TNFRSF9 antibodies in B16 melanoma in mice [65]. Given the size of the model, additional experimental data would further improve model parameterization and robustness [66].

In summary, cell-population models can combine clinical and cell culture data and might support the determination of an individualized drug regimen based on cellular dynamics. While these models are suitable for freely acting cells, tumors are frequently restricted by the ECM and anatomic space limitations. These effects were simplified by three models mentioned above [48, 51, 65]. While one refers to threedimensional (3D) spheroid growth in collagen gel, two refer to tumor size in mice. Tumor growth is more complex and requires spatial, mechanical, and physiological characteristics being addressed in the following three sections.

## Spatial models of melanoma

The spatial tumor expansion in tissue has played a subsidiary role heretofore. In the following, spatially distributed factors of lesions and environment will be addressed. For instance, spatial patterns in dermoscopic pictures can be used to classify a particular lesion to obtain hints for prospective growth and the necessity of surgical intervention. Subsequently, combining cell-population models with geometry provide insights into the success of surgical therapy. When focussing on the cellular level, the positioning and shape of cells can hint to mechanical and thus biochemical factors, which stimulate local cell mass expansion. However, a more in-depth insight into histological features of skin and other host tissues indicate that solely geometrical solutions may not be sufficient as mechanical cues significantly impact deformation and development.

### Pattern recognition of melanoma

The pattern of naevi and melanoma in situ are the physical consequence of biochemical processes in the epidermis and are usually assessed and classified in dermatology to initiate early therapy. The related patterns can be modeled in two dimensions using a mixture theory model [67]. The study shows how different patterns of malignant cells can form within a healthy cell environment. two-dimensional (2D) patterns of naevi and melanoma can also be subjected to planar linear transformations using two subsequent dermoscopic pictures. Those pictures allow the classification of melanoma growth rates and naevi symmetry [68]. The ABCD criteria for asymmetry, border irregularity, color variation, and diameter of melanoma have been mathematically considered too [69].

Automated optical classification of naevi and melanomas is a fast-growing field and employs machine learning methods for image recognition. The sensitivity and specificity of these models coincided with the decision quality of dermatologists [70–72]. Specific features in 2D dermoscopic images can also be used to determine the Breslow depth with specificity and sensitivity of almost 100%, which has direct prognostic value [73]. Furthermore, the depth of invasion is an important prognostic marker for patient survival, and the Breslow index can be determined manually or automatically from histopathological images [71, 74].

### Models of surgical treatment

Surgical treatment is the best option for early identified melanomas. However, wide excision of primary melanoma can have counter-intuitive ramifications according to the reaction-diffusion model of Eikenberry et al.. The surgical resection of primary melanomas might include tumor-associated immune cells, which lead to an accelerated outgrowth of local metastasis due to reduced immune suppression [75]. Computational models are also used to assist image-guided and computer-assisted surgery, mainly for the brain [76]. The brain, besides lung and lymph nodes, is a preferred host tissue for metastatic melanoma [77].

### Dissecting parameters in spatial models is a challenge

Fully experimentally validated models of melanoma expansion are still limited to data based on 2D cell culture experiments. In a series of reports, Treloar et al. used a lattice cellular automaton model and an experimental approach to identify different parameters of colony growth, where cell motility, cell–to–cell adhesion, and cell proliferation influenced the same: the expansion of the cell colony [78, 79]. These parameters were also estimated using a Bayesian framework coupled with a stochastic model of 2D melanoma growth [80]. Using melanoma and fibroblast monocultures as well as different co-culture systems, Haridas et al. have parameterized a partial differential equations (PDE) model of the interactions of cancer cells and fibroblasts [81]. Continuous modeling of melanoma cells under different osmotic pressures was performed with a 2D lattice model to simulate scratch assays [82]. The aim was to differentiate migration/invasion between primary and metastatic cells. New vertex modeling strategies [83] and scratch assay analysis tools [84] might further improve this approach.

### Spatial organization of skin and confined spaces

The previously described spatial parameter determination strategy for cell lines is especially helpful for the epidermal skin layer. However, the skin is more complex and also contains irregular fibrous tissue beneath the epidermal layer separated by a collagenous basement membrane [85]. At the dermal-epidermal junction, keratinocytes are generated and stratify through the epidermis up to the skin surface, where they keratinize to form the protective barrier called stratum corneum. The epidermal layer is also the most common location for melanoma initiation. Residing melanocytes can become benign neoplasms and appear as innate or acquired naevi [77]. Further changes and appearing atypical cells constitute the first malignant stage: the radial growth phase. From the clinical perspective and the perspective of modeling, the basement membrane is crucial. Invasion through the basement membrane indicates the vertical growth phase, which may require adjuvant therapy besides surgical treatment. Pharmacological therapy is implicated for metastatic growth into secondary tissues. In contrast to the epidermis, the dermis layer is streaked with collagen and elastin fibers synthesized by fibroblasts [86], and these ECM fibers restrict tumor expansion [87].

Using colony growth in 2D cell culture experiments does not lead to quantitative parameters for spatial models representing stromal processes. For example, migration velocity depends on the ECM fiber geometry [88], the migration process is fundamentally different in confined structures [89], and depends on the paxillin (PXN) and transforming growth factor beta 1 induced transcript 1 (TGFB1I1) balance related to PTK2 [90]. Moreover, BRAF inhibition promotes matrix metalloproteinases (MMP) activity and cell migration in three dimensions [91]. A consequent experimental parameterization of realistic melanoma growth models is difficult to find and is aggravated by the diversity of parameter origins and their mutual dependency, as shown by Treloar et al. [78, 79]. The modeling of the tumor microenvironment has to consider additional factors like extracellular matrix stiffness and topography, oxygen and nutrients gradients, and interstitial fluid pressure [92].

## Mechanical models of melanoma

Mechanical cues in the environment directly influence important biochemical cancer pathways and have a complex impact on tumour progression [93, 94]. Consequently, mechanical models get more attention and three methods will be presented in the following: mixture theory, the thermodynamically constrained averaging theory (TCAT), and the discrete ansatz with cross-linked elastic cells. These three methodologies can mimic the growth in tissues, while a tissue without any malignant contortions is already a complex modelling task [95]. As the integration and measurement of mechanical cues is not yet widely used, a summary of experimental methods is given below.

### Impact of mechanoregulation

In three dimensions, additional factors impair drug sensitivity [49, 96] and either increase or decrease the tumor growth rate [97]. The stromal environment causes non-genetic phenotype switches between proliferative and mesenchymal stages [98, 99], and environmental melanoma-associated fibroblasts are known to play an essential role in melanoma progression [100]. Fibroblast activity is closely linked to ECM and thus biomechanics, which is now recognized as a central pillar of tumor progression and metastasis [93, 94]. Mechanical melanoma models consider the growth-induced deformation of the ECM rich environment. The more the proliferating mass expands, the more counterforce is generated by the connected ECM fibers. The elastic energy is conserved and geometry dependent [87]. The mechanical deformation of tissues and mechanical stress influence intracellular signaling by mechano-sensors like PTK2 [101] or YY1 associated protein 1 (YY1AP1)/tafazzin (TAZ) [102], which are discussed as one drug resistance mechanism for BRAF-mutant melanoma cells [38, 49, 103] or progression marker for cutaneous and G protein subunit alpha q (GNAQ) mutant uveal melanoma [104–106]. Proximity to mechano-regulating fibroblasts can induce pathway changes to PIK3CA/mechanistic target of rapamycin kinase (MTOR) and switch the phenotype of melanoma cells to the mesenchymal state [107]. Consequently, melanoma cells reduce the inherent stiffness to facilitate invasion [108, 109]. However, our knowledge of mechanosensitive pathways is far from complete [109–112], and comprehension of mechanical phenomenons require computational models. Additionally, the skin, being the primary site for cutaneous melanoma, is a mechano-sensitive organ. Skin can grow when it is stretched, and rete ridges, projections of the epidermis into the dermis, were recently suspected to form according to mechanical characteristics [86]. The skin has inspired many computational models describing dermal transport processes as well as providing a mechanical understanding of the skin’s optical, functional, and structural characteristics [113].

### Mixture theory

Two mixture theory models exist. One describes the skin surface, and one mimics the vertical section [67, 114]. Balois et al. consider interstitial fluid pressure, a mechanically optimal cell density, and friction between the melanocytic lesion and the surrounding tissue. Ciarletta et al. represent melanoma in the radial growth phase in the ECM free epidermis as a viscous fluid sliding on a basement membrane with friction dependent growth velocity. In a second step, this friction is neglected and instead considered between the basement membrane and an additional keratinocyte representing fluid. Melanoma cell and keratinocyte fluid are adjacent to each other, and the tumor front between them is a moving interface/ free boundary problem subjected to stability analysis [114].

### TCAT theory

TCAT models [115] represent a multi-phase approach, which is different from the mixture theory and circumvents the free boundary problem. TCAT models do not have a defined tumor boundary at the macro-scale, but the ECM spans the whole tissue, with a higher concentration at the basement membrane. The interstitial fluid, the healthy, and the malignant cells squeeze via local rules through the solid but deformable porous ECM network. Averaging of local properties causes a macroscale behavior that resembles the distortion of the tissue and the invasion of the basement membrane. By adjusting the cancer cell plasticity but not ECM integrity, the model changes from solid to invasive growth [116].

### Disordered lattice model

The discrete model [117] describes individual cells on a 2D disordered lattice. Cells are represented as spheres, which are connected via breakable springs. The springs mimic ECM and cell-cell contacts. Melanoma induced MMP activity is modeled by a higher probability of spring breaking near melanoma cells. Despite the simple mechanical and geometrical laws, the simulation results give a realistic impression. Because discrete models are more computationally demanding than continuous models, they allow only limited upscaling. However, the benefit of this single-cell modeling approach is the potential discrimination between compressive and tensile stress, which can differ strongly across the ECM biopolymer types [118] and tumor locations [87]. The model by Taloni et al. was validated with 2D experiments. The experiments were performed under osmotic pressure without fibronectin, which is an important linker between mechanics and intracellular signaling.

### Experimental methods for mechanical melanoma models

Although modeling promises to become more and more important in melanoma research, and continuous improvement of the computational power make more complex and realistic models accessible, experimentally validated parameterization remains a crucial bottleneck. To produce high-quality mathematical models, quantitative data under standardized operating procedures are required [119, 120]. Tumor spheres and spheroids in general [121], and organotypic in vitro models of melanoma [122] in particular offer more realistic experimental conditions. Fully functional organotypic skin constructs [123] can mimic all melanoma progression stages. 3D constructs do not only serve as carrier of cells; they modify the experimental outcome. Thus, quantification of hydrogel system parameters, such as the shear or Young’s modulus becomes standard. The shear modulus G of the gel system, or the roughly three times higher Young’s modulus E, is stated with the unit kPa (E=2G(1+ *ν*); *ν*: Poisson’s ratio) [49, 101, 111]. Knowing the impact of mechanical cues in the modeling process prevents common data integration problems. For example, the frequently used matrigel for invasion assays has an elastic modulus of 0.45 kPa and is consequently a weaker barrier than the basement membrane reaching 250-500 kPa [124, 125]. Additionally, the impact of stress relaxation should be not underestimated as it has a decisive impact on further development [126]. A range of hydrogel systems is available [127] and can also be used for automated drug testing [128] albeit questions of standardisation of 3D cell culture models remain to be addressed [129]. Mechanical parameters are difficult to measure and span up to 5 log steps depending on tissue moisture and experimental setting [130]. Experimental mechano-sensors enable the measurement of sub-molecular force transmissions [131], and fluorescent oil microdroplets allow the measurement of anisotropic stress fields in 3D tissues [132]. Tunable alginate microcapsules can be used to determine the mechanical growth-pressure of spheroids [133], and high throughput mechanical testing of cells is possible with optical deformation of cells [134]. If the direct measurement of stiffness is not possible, the lamin A/C (LMNA) to lamin B1/2 (LMNB1/2) ratio serves as an appropriate biomarker of stiffness sensing [135]. The clinical imaging technology elastography gives direct access to the tissue stiffness fields and thus tumor locations in vivo [136]. Elastography can also be used for the in vivo staging of melanoma [137]. The integration of elastography and melanoma mechano-signaling could highlight stiff areas where mechano-sensors influence melanoma signal transduction. This could facilitate the translation of these research models into clinically relevant predictive models. The complexity of the interaction of tumor cells with their environment requires a step-wise understanding with a multitude of experimental techniques [138] and related computational efforts (Fig. 1). Computational scientists must incorporate the experimental context to develop meaningful computational melanoma progression models.

## Transport of oxygen and drugs

Several models describe how oxygen and drugs are transported from the source to a melanocytic lesion as the presence of oxygen and nutrients control the viability of cancerous and healthy tissues. For melanoma, multiple oxygen sources as well as vascular and pericellular transport routes influence tumour progression as described in the following.

### Oxygenation of melanoma in skin and brain

Impaired oxygen and nutrient delivery cause necrotic cores, which is a widespread assumption. A necrotic core can be modelled explicitly [115] or indirectly via nutrient concentration reduction [114]. However, Thibaut Balois & Martine Ben Amar questioned the existence of necrotic cores in epidermal melanoma and took the atmospheric oxygen source into account [67, 139]. If oxygen came only from the dermal vasculature, the oxygen partial pressure would drop to around eight mmHg at the skin surface [140]. Mild hypoxic conditions are present around the basement membrane, promoting melanocyte proliferation [141] as well as melanoma progression [142]. Interestingly, the brain, a common location for metastasized melanoma, has also a low tissue oxygen concentrations reaching 35 mmHg [143].

### Experimental aspects of oxygen

Most established melanoma cell lines are cultured under atmospheric oxygen and are therefore evolutionarily adjusted to these artificial conditions. Molecular oxygen sensors for 3D settings [144] are as possible as advanced hypoxia sensors [145] to improve the validation of computational models. Oxygen consumption rates of cells can be obtained with the Seahorse technology [146] and were determined for melanocytes and melanoma cell lines [147].

### Models of melanoma-associated vascularization

The tumor-associated vascularization is influenced by oxygen limitations and mechanical cues [148, 149]. Mathematical blood vessel models define an independent computational research field [150]. Notably, Welter and Rieger combined the discrete modeling of vasculature remodeling with the continuous gradients of melanoma cells, oxygen, nutrients, and drugs [151]. They used melanoma-specific data for the vasculature [152]. This model is useful to simulate blood flow and to study the impact of space limitations on simple drug diffusion and nutrient supply. Wang et al. created an agent-based model containing both melanoma and endothelial cells with a focus on angiogenesis. They tested the combined effect of doxorubicin chemotherapy and kinase insert domain receptor (KDR)^2^ inhibition with sunitinib [153]. It might be interesting to see a follow-up model with improved use of biological data for parameter, synergy, and validation. Dzwinel et al. coupled several continuous sub-models of melanoma growth to increase modeling quality and efficiency. They used a single phase continuum for growth accompanied by angiogenesis, vascular remodeling, and tumor ECM interactions. The model was embedded in a realistic virtual skin structure, and the melanoma progression resembled nodular, lentigo maligna, and acral lentiginous melanoma [154]. The same group extended the model by a discrete vascularization dynamic, which was coupled intermittently [155]. The used approach, called “super-modeling” by the authors, is a theory on model synchronization [156]. However, the connection coefficients seem untrained in comparison to non-biological application areas, and the coupling remains weak [156]. This modeling group is very active in melanoma, refines the model continuously, and also uses particle automata models to produce visually realistic models [157, 158]. Taken together, while these models provide valuable insight into the vasculature, much work is needed to ensure adequate melanoma-specific parametrizations and validations. Einar Rofstads’ group provides excellent data sets on melanoma-associated vascularization and might be considered for further modeling projects [159].

### Drug delivery models

Blood vessels are an essential route for drugs to the location of action, and pharmacokinetics is studied to determine the drug concentration in local blood plasma. However, the transport from the blood vessels or skin surface to the melanoma cells depends on the diffusion coefficient of the microanatomical structure. Drug delivery models are available for both the penetration of spherical tumors with melanin-binding antibodies for radioimmunotherapy [160] and SPACE-EGF mediated transdermal delivery of siRNA against the MYC proto-oncogene, bHLH transcription factor (MYC) [161]. However, the impact of biomechanics and tumor physiology on drug delivery is not considered by those models but discussed for MU89 melanoma in mice [162, 163]. A proliferating mass makes fibrous tissue crowded and compressed. This might lead to a reduced interstitial fluid volume fraction and thus impaired drug transport. Such a phenomenon might be best modeled with the multi-phase flow in porous media [115].

## Discussion

The generation of a mechanistic and predictive model is a serious and work-intensive endeavor that forces all participants to think deeper [9]. Ultimately, the reward is more aim-tailored research but also the discovery of hidden causalities, which would otherwise have rendered explorative research inconclusive or contradictory. Recent progress in devising experimental procedures for parameter determination has fueled the work of several computational groups. Conversely, certain phenomena can only be understood with computational methods, such as computational mechanics. Mathematical modeling of melanoma presents several specificities ranging from the high mutation load and cell plasticity to oxygen uptake at the skin surface. Nevertheless, most of the current models of melanoma are not yet sufficiently adapted to the requirements in biology and medicine. The recurring problems in almost all reviewed research can be expressed in four challenges and are discussed accordingly.

### First challenge: tumor heterogenity

The first challenge is the cellular heterogeneity. The high mutation load, signaling network plasticity, and cell line heterogeneity makes the fitting of mechanistic ODE systems or straightforward network inference from patients’ biopsies difficult. Instead, most studies focus on well-characterized cell line collections to carefully extract specific regulatory network motifs with multivariate statistics. The cell line-specific models are suitable for understanding drug responses. Notable works used systems biology to investigate the impact of new compounds such as TRAIL [36, 40], while others focus on identifying potential targets by perturbing the biological system with several kinase inhibitors [37, 39].

### Second challenge: melanoma type specifity

The second challenge is melanoma type specific modeling. Melanocytic tumors occur in various forms at different locations and are based on different etiologies [164]. A few important types are lentigo maligna melanoma, superficial spreading melanoma, and acral lentiginous melanoma. Nonetheless, computational papers often refer to a general term of melanoma, albeit each melanoma type can substantially differ in treatment response, environmental setting, and growth pattern. In computational biology, mechanistic links between growth patterns and melanoma-type specific biochemical markers could prospectively reach the same importance as in pathology [77, 164]. Instead of constructing models around a few abstract mathematical parameters and retrospectively allocate histopathological sections to a given simulation outcome, modelers might emphasize the pathological causality and relevant biochemical root-causes leading to a melanoma-type specific growth outcome. A deeper examination of cancer pathology, anatomy, and physiology might also prevent unjustified assumptions. Some authors set initial lesions at positions, where they rarely occur, such as the epidermal stratum corneum, albeit the stratum basale is often the location of initial lesions [164]. The unique oxygen patterns in skin [139, 140], the tendency of melanocytes to proliferate better in mild hypoxic conditions [141], the strong oxygen consumption of melanoma cells [147] as well as the importance of driver mutations in this highly mutated cancer type [15, 16] are further factors, which might find more consideration by modelers of melanoma. Not all concepts, model structures, and parameters can be taken from models of other cancer types. Future melanoma models might represent more melanoma type specific characteristics and parameters, whereby attention should also be drawn to the respective histopathology and the host tissue in which the simulated melanoma is intended to be simulated. Eventually, context and tissue-specific modeling of certain melanoma types is more insightful than generic cancer or melanoma models.

### Third challenge: complexity

The third challenge is the appropriate level of complexity as neither very small and simple nor extensive models can deliver reliable predictions. Models that are as simple as possible are the gold standard in modeling, as shown by Kim or Picco et al. [51, 52]. However, if models neglect major effects, or the remaining model elements are too abstract to be interpreted, the result will be of little use. For example, careful work was performed to determine mutually dependent parameters of a cell colony [81]. However, the impact of the mechanical environment on these parameter values [94], such as migration [88], exacerbates the transfer of these parameters to complex 3D models. On the contrary, especially large scale models which have been set-up [46, 153] will benefit from sufficient and appropriate melanoma-specific data, to further increase the validity of their conclusions. The same can be observed in physical oncology. Mixture theory allows easier models and fewer parameters. Still, it is difficult to measure abstract parameters or to biologically interpret the equations as they pool too many biological sub-systems to homogeneous entities. In contrast, Sciumé et al. accurately differentiates between cells, fibrous compounds, and interstitial fluid. This makes experimental parameter determination easier and aligns better with medical and biological lines of thinking. However, the model requires many parameters, which must still be biologically validated. Best interdisciplinary communication is reached with agent-based models, were cells are separately depicted. However, the computational demand for simulating individual cells is substantial. More experience is necessary to find the right level of complexity that can be of practical use and allow both computationally feasible and biologically sound models.

### Fourth challenge: correct data integration

The fourth challenge is the accurate integration of evidence. Experimental facts and assumed fiction are difficult to distinguish in many publications, and it is of little use if an extensive biological section is written independently of the computational part but is hardly reflected by the equations at the end [114]. To enable scrutiny by melanoma experts and to facilitate evidence-based model extensions or improvements, it seems necessary that each element of a model structure is biologically explained, interpreted, and referenced only within the degree of factual implementation, even if this requires extensive supplemental information. The reasoning behind modeling decisions should be accountable. At least, the behavior of all system elements should be tested for plausibility as done in one work [45]. Besides the verifiability of the model structure, parameters are very ambiguous in most papers, and only a few papers provided supplemental information about data extraction and conversion [53]. Unfortunately, many publications work with uncurated parameter lists, and interested readers are recommended to trace back parameter values to the primary source to judge the validity. We found that data indicated as melanoma-specific were based on other diseases and tissue origins such as glioblastoma or the adrenal gland. Estimated or assumed parameters were often referenced in subsequent papers as if they were experimentally determined values. Moreover, the context of experimental origin often do not fit the intended model context, or whole parameter sets are normalized in an original paper and then carried over several computational paper generations regardless of biology studied. In order to bring models closer to biological evidence, parameters should be referenced only to the original experimental publications, and information should be given on the experimental context and potential parameter conversions. Not all required data are available, but transparency on evidence is generally lacking. It remains to be debated to which extend a model must contain melanoma-specific data to be considered a melanoma model or how close a model must match medical evidence to be seen as a valuable contribution to melanoma research.

### Lack of interdisciplinary is the root cause

These four challenges reflect the most persistent problem of melanoma-specific modeling: interdisciplinarity. Computational models require close collaboration between experimental, clinical, and computational scientists in an iterative procedure. Modeling generates hypotheses, which have to be tested in vitro, and experimental results must inform the design of better models and allow the falsification of theories [165]. However, a general problem in the interdisciplinary work in biology and medicine is that the more demanding the necessary mathematical and physical framework becomes, the more disconnected it becomes from the experimental and theoretical knowledge in biology, medicine, and pharmacology. On the one hand, computational groups cannot reproduce and test the diverse parameter sources in their labs, lacking the time and expertise to embrace the whole complexity of biological relationships and experimental methods. On the other hand, biologists and clinicians find it difficult to help, as the more developed computational procedures are likewise difficult to comprehend. Therefore, better quality standards between and in both computational [166] and biomedical research [167] need to be developed and adopted. A more sophisticated way might be the stepwise model development accompanied by advanced cell culture strategies (Fig. 1). The gap between the different disciplines is not closed yet, which leads to conceptual problems in the models and inappropriate parameter choices.

## Conclusion

Cancer is a highly complex, heterogeneous disease, characterized by a series of genetic, metabolic, and functional changes at the cellular and tissue level [168]. Melanoma-specific dynamics during tumor progression stages in both plasticity [108] and genetics [169] highlight the need for integrative models to better understand disease mechanisms of melanoma. The model-building community works across different scales and comprises studies centered on signaling pathways and gene regulation [22], metabolism [42, 170], epithelial tissue mechanics [171], tumor physiology [172], and the immune system [173], respectively. Four challenges for computational melanoma models have been discussed:

- Melanoma heterogeneity,
- Melanoma-type specificity,
- The balance between simplicity and thoroughness, and
- Melanoma data integration and evidence.

Consequently, interdisciplinarity and clinical relevance remain a challenge regarding the practical use of melanoma-specific systems biology and physical oncology models. However, if all disciplines improve interaction and collaboration, the future promises us an unmatched insight.

## Supplementary information

**Additional file 1** Supplementary table 1: Melanoma model overview. Appreviation: C=cellular, CP= cell population, T=tissue, O=organ, PP=patient population, 0D-3D : zero to three dimensional.

## Data Availability

Not applicable.

## Abbreviations

All biomolecule names in this thesis are approved by the HUGO Gene Nomenclature Committee (HGNC) of the Human Genome Organisation (HUGO). 2D: Two-dimensional
3D: Three-dimensional
AKT1: AKT serine/threonine kinase 1
BRAF: B-Raf proto-oncogene
CTLA4: Cytotoxic T-lymphocyte associated protein 4
CTNNB1: Catenin beta 1
c-Myc: BHLH transcription factor
DTIC: Dacarbazine
DNA: Deoxyribonucleic acid
ECM: Extracellular matrix
GNAQ: G protein subunit alpha q
HGNC: HUGO Gene Nomenclature Committee
HIF1A: Hypoxia inducible factor 1 subunit alpha
HUGO: Human Genome Organisation
I **κ** B **α**: NF- **κ** B inhibitor alpha
IL21: Interleukin 21
JUN: Jun proto-oncogene, AP-1 transcription factor subunit
KDR: Kinase insert domain receptor
LMNA: Lamin A/C
LMNB1/2: Lamin B1/2
MAP2K: Mitogen-activated protein kinase kinases
MAPK1: mitogen-activated protein kinase 1
MAPK8: mitogen-activated protein kinase 8
MGDB: Melanoma Gene Database
MMMP: Melanoma Molecular Map Project
MMP: matrix metalloproteinases
MTOR: Mechanistic target of rapamycin kinase
MYC: MYC proto-oncogene, bHLH transcription factor
R1I2: NR1I2 nuclear receptor subfamily 1 group I member 2
NK: Natural killer cells
NRAS: NRAS proto-oncogene, GTPase
ODE: Ordinary differential equations
PDCD1: Programmed cell death 1
PDE: Partial differential equations
PIK3CA: Phosphatidylinositol-4,5-bisphosphate 3-kinase catalytic subunit alpha
PTEN: Phosphatase and tensin homolog
PTK2: Protein tyrosine kinase 2
PXN: Paxillin
RAS: RAS type GTPase family
SRC: SRC proto-oncogene, non-receptor tyrosine kinase
TAZ: Tafazzin
TCAT: Thermodynamically constrained averaging theory
TCGA: The Cancer Genome Atlas
TRAIL: Tumor necrosis factor related apoptosis inducing ligand
TGFB1I1: Transforming growth factor beta 1 induced transcript 1
TNFRSF9: TNF receptor superfamily member 9
UVB: Ultraviolet B
XIAP: X-linked inhibitor of apoptosis
YY1AP1: YY1 associated protein 1

## Footnotes

1 Also known as c-Jun N-terminal kinases JNK

2 Also known as vascular endothelial growth factor receptor 2 (VEGFR)
