# Supplementary material for: Computational models of melanoma
Source: Theor Biol Med Model. 2020 May 14;17:8. doi: 10.1186/s12976-020-00126-7 (PMC7222475; doi:10.1186/s12976-020-00126-7)
Supplement: Supplementary file 1 — Additional file 1 Supplementary table 1: Melanoma model overview. Appreviation: C=cellular, CP= cell population, T=tissue, O=organ, PP=patient population, 0D-3D : zero to three dimensional. [file 12976_2020_126_MOESM1_ESM.pdf]

Review

Computational Models for Melanoma

Authors:

Marco Albrecht, University of Luxembourg, Luxembourg, marco.albrecht@posteo.eu

Philippe Lucarelli, University of Luxembourg, Luxembourg, philippe.lucarelli@brcch.org

Dagmar Kulms, Technical University Dresden, Germany, Dagmar.Kulms@uniklinikum-dresden.de

Thomas Sauter, University of Luxembourg, Luxembourg, thomas.sauter@uni.lu

Corresponding Author:

Prof. Dr. Thomas Sauter

Professor of Systems Biology

Life Sciences Research Unit

Université du Luxembourg

6, avenue du Swing

L-4367 Belvaux

Campus Belval

Building Biotech 2 (BT2), Room 4.05

Tél.: +352-46 66 44 6296

Fax: +352-46 66 44 6435

e-mail: thomas.sauter@uni.lu

http://bio.uni.lu/research_areas/systems_biology

http://misb.uni.lu

Dear Prof. Hiroshi Nishiura,

I am writing to re-submit our manuscript entitled, "Computational Models for Melanoma" for consideration as a *Theoretical Biology and Medical Modelling* review article. The title has been modified following a reviewer's comment. The review structures the diversity of mathematical modelling approaches around the common topic melanoma and thereby emphasizes the modelling peculiarities, which must be considered specifically for the cancer type melanoma.

We would like to thank the reviewers once again for carefully reading our manuscript and for their valuable comments which we addressed point-by-point as listed in the attached document.

The review originated during the EU funded MEL-PLEX programme: Exploiting MELanoma Disease ComPLEXity to Address European Research Training Needs in Translational Cancer Systems Biology and Cancer Systems Medicine. This review has been included in the PhD thesis of the first author Marco Albrecht who defended successfully in April 2019 at the University of Luxembourg.

Each of the authors confirms that this manuscript has not been previously published and is not currently under consideration by any other journal. Additionally, all the authors have approved the contents of this paper and have agreed to the *Theoretical Biology and Medical Modelling* submission policies.

Each named author has substantially contributed to conducting the underlying research and drafting this manuscript. Additionally, to the best of our knowledge, the named authors have no conflict of interest, financial or otherwise.

Sincerely,
Thomas Sauter

Professor of Systems Biology
Study Director

Life Sciences Research Unit
Université du Luxembourg
6, avenue du Swing
L-4367 Belvaux

Building Biotech II (BT2), Room 4.05
Tél.: +352-46 66 44 6296
Fax: +352-46 66 44 6435
e-mail: thomas.sauter@uni.lu
http://bio.uni.lu/research_areas/systems_biology
http://misb.uni.lu

Reviewer reports:

We would like to thank the reviewers for carefully reading our manuscript and for their valuable comments which we addressed point-by-point as listed below.

Reviewer #1: [Overall comment]

I'd like to appreciate the author's effort to improve the manuscript. Overall, the manuscript was edited in a clear way.

Background were remarkably improved by adding detail examples from the previous studies.

Conclusion section helps readers understand major challenges better.

I'd like to suggest minor comments and ask simple questions.

[Major comment]

Configuration of the manuscript

There are four main points for computational melanoma models; (1) Melanoma heterogeneity, (2) Melanoma-type specificity, (3) the balance between simplicity and thoroughness, and (4) Melanoma data integration and evidence. They were not highlighted well in the manuscript. I think the configuration of the manuscript is required to reveal the four challenges.

In addition, it doesn't need to be numerated because section 3 has only one subsection.

Section 4 has 10 subsections which are too many subsections compared to other sections.

For example, I think section 4 can be divided into three subsections such as 4.1-4.3, 4.4-4.6, 4.7-4.10. I suggest to consider the configuration of the manuscript especially for section 3-4 to highlight the main results.

Thank you very much for this advice. We restructured the paper accordingly and provide a better and more evenly spaced review. We also adjusted introducing and summarizing sentences. The main purpose of the review is to get an overview on melanoma-specific computational models to stimulate improvements in all parts. We feel that the reviewed problem-tailored research work should be valued individually in their specific context. All four mentioned challenges apply to any chapter in the maintext. While the main text follows closely model structures along the respective medical perspective, the discussion opens an additional dimension to crosslink the reviewed article according quality and interdisciplinary challenges. We thus abstained from re-iterating again in the discussion on each model which would make the discussion too lengthy from our point of view. The review is now structured as follows:

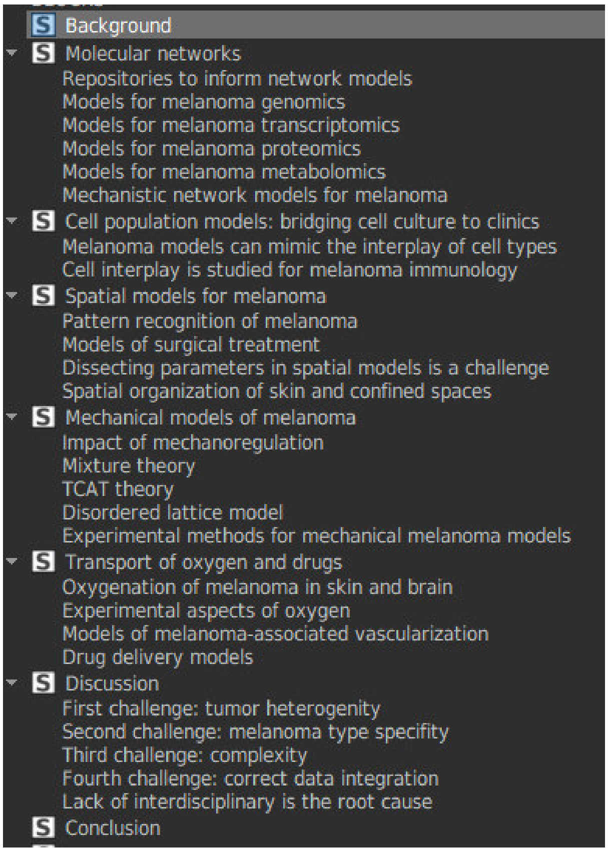

[Minor comment]

1. The Melanoma Gene Database (MGDB) was addressed in section 1 for the first time. Reference [175] can be cited in P2 line 52.

We corrected this accordingly.

2. In section 2, melanoma-specific modeling studies were introduced. I think it's better to describe why melanoma-specific modeling is important as authors explained from the reference [163] in Discussion in P9 line 5-10.

There is a difference between the first and the latter. The frequently occurring term "melanoma-specific" generally describes the larger group of melanocytic tumours and is the level of most reviewed research work. It is basically explained by the title of the review. In contrast, we used the term "melanoma-**type** specific" in the discussion because we believe that most papers do not tackle an actual disease but rather a group of many diseases. Now, it is not reasonable to take parameter from disease A, the initial condition from disease B, and to compare this with data of disease C.

3. In P3 line 56-60, authors described that "the interactions with the environment, the restriction to only one cell line, … in decision-making [42]." It's not clear why physicians might feel difficult to be supported in decision-making.

We agree. We also found that the sentence is rather fits into the discussion and thus removed it here.

The answer to this question has many facets and depends on how far each modelling discipline currently is. Of course, experts know all the details of their subject and do not need help from outside. However, it might be beneficial to hardcode the expertise and see whether it aligns quantitatively with data and to proof that an

understanding/research hypothesis is predictive. Integrating data to get predictive models becomes especially important if new evidence accumulates faster that experts can assess and evaluate or if interdependencies become too numerous. However, as long as the expertise is not sufficiently considered and models are not set up fast enough in sufficient quality, models will not be very useful for decision-making.

4. In P4 line 48, the role of "Akaike information criterion" was not clearly written. It is well known that a model selection procedure can be applied by using Akaike information criterion (AIC). I'd like to ask to describe how AIC was used.

Thank you very much for pointing this out. We removed the hint to the AIC to avoid distraction from the scope of the review. Moreover, the problem was rather that neither AIC nor a similar approach had been used. Thus, AIC was suggested as one of many ways to improve model quality. However, naming only one way without giving a whole picture might unintentionally overlook important research work on model selection procedures and might not live up to the expectations of the reader.

Reviewer #2: I am satisfied with revision made by the reviewers. I think their paper will be a good addition to TBMM.

Response to the reviewers:

- The following change made me confused: "The unique oxygen patterns in skin, the tendency of melanocytes to proliferate better in mild hypoxic conditions, the strong oxygen consumption of melanoma cells, or the importance of driver mutations found a rare propagation in the modeling community, [...]" Why is it a rare propagation in the modeling community? For example, one the latest papers of "Lahouel et al Vogelstein, Geman, Tomasetti 2019 Revisiting the tumorigenesis timeline with a data-driven generative model PNAS" and recently published "Rozhok deGregori 2019 A generalized theory of age-dependent carcinogenesis eLife" are both in between modeling and data, with implementation of concept of driver mutations. I agree it can be viewed a bit distant from approaches of systems biology to carcinogenesis, but the authors could be careful in choosing their words and not to call it "rare".

Thank you very much for this comment. We rephrased the assessment accordingly:

"The unique oxygen patterns in skin [], the tendency of melanocytes to proliferate better in mild hypoxic conditions [], the strong oxygen consumption of melanoma cells [], or the importance of driver mutations in this highly mutated cancer type [] are further factors, which might find more consideration by modelers of melanoma. ~~might find also more consideration found a rare propagation in the modeling community, which too often copies~~

Minor:

- I recommend to re-phrase the title a bit, because "approaches" may be the verb, and every time I read it I stop in the middle with some confusion. Maybe "Approaches of systems biology...". But then, approaches to what exactly?

We rephrased the title to "Computational Models of Melanoma"

- L12: I would recommend to rephrase "their melanoma research questions", because it sounds like research questions regarding melanoma of a reader.

We rephrased to: “[…] to address their research questions about melanoma”

RESEARCH

Computational Models of Melanoma

Marco Albrecht[1], Philippe Lucarelli[1], Dagmar Kulms[2] and Thomas Sauter[1*]

Abstract

Genes, proteins, or cells influence each other and consequently create patterns, which can be increasingly better observed by experimental biology and medicine. Thereby, descriptive methods of statistics and bioinformatics sharpen and structure our perception. However, additionally considering the interconnectivity between biological elements promises a deeper and more coherent understanding of melanoma. For instance, integrative network-based tools and well-grounded inductive *in silico* research reveal disease mechanisms, stratify patients, and support treatment individualization. This review gives an overview of different modeling techniques beyond statistics, shows how different strategies align with the respective medical biology, and identifies possible areas of new computational melanoma research.

Keywords: melanoma; systems biology; physical oncology; tumor growth

1 Background

Melanoma is a neoplasm of the skin and originates from transformed melanocytes. It causes the loss of 1.6 million disease-adjusted life-years worldwide, and the incidence rate will increase in the next decades [1]. Since the discovery of the high prevalence of mutations in b-Raf proto-oncogene (BRAF) and NRAS proto-oncogene, GTPase (NRAS) [2, 3], small-molecule inhibitors such as dabrafenib and vemurafenib were developed. More recently, immunotherapies, with antibodies binding immune receptors like the cytotoxic T-lymphocyte associated protein 4 (CTLA4) or the programmed cell death 1 (PDCD1), have proven clinically effective [4]. However, many drug resistance mechanisms occurred and represent a major problem in both targeted therapy and immunotherapy [5, 6, 7]. As a result, life expectancy remains low. The two-year survival rate is 53.5% for combined BRAF + mitogen-activated protein kinase kinases (MAP2K) inhibitors and 63% for combined CTLA4 + PDCD1 immunotherapy [8]. Consequently, a deeper understanding of disease mechanisms is still demanded.

An approach to better understand causative relations, to check hypothesis consistency, but also to reveal missing qualitative information is constructing evidence-based models of these biological systems [9]. Models depict several interconnected biological elements with a structure, which is derived from the current understanding, and parameters, which are based on data. While many life-scientists still rely on straight-forward relationships between observation and insight to extend their knowledge, leading scientists report that the direct link between observation and insight seems to fade [10]. Thus, experimentally proven relationships are increasingly transferred into the language of mathematics to enhance our understanding of experimental findings and underlying causes.

Cancer scientists can benefit from well-designed computational models, whereby systems biologists deliver models of cancer biochemistry, and physical oncologists provide models of tissues. Systems biology helps understanding how biochemical pathways change during melanoma cell proliferation, invasiveness, survival, and drug resistance based on network structure and dynamic behavior [11]. By contrast, physical oncology helps understanding how transport, growth, and deformations in tissues occur and is characterized by principles of geometry and mechanics [12, 13].

In this review, we tried to gather all published computational models of melanoma and describe them regarding their contribution to the field. In particular, we focus on the interconnection of system elements or network characteristics while omitting classical statistics and bioinformatics of melanoma. By sorting models and methods around the topic of melanoma, we intend to support readers in finding the most appropriate mathematical models to address their melanoma-specific research questions. Additionally, the review shall describe potentials for improvement, encourage readers to discover potential extensions, and create

*Correspondence: thomas.sauter@uni.lu
[1]Systems Biology Group, Life Science Research Unit, University of Luxembourg, 6, avenue du Swing, 4367 Belval, Luxembourg
Full list of author information is available at the end of the article

awareness of wholly missing melanoma topics to be tackled in the next decade. However, even if some models seem simplistic in biology, they often represent technically challenging stepping-stones for more biologically meaningful models in the future. Consequently, reviewing the currently existing models shall help to push forward the modeling and computational characterization of melanoma.

The review is structured as follows: Network-based approaches are explained in section two and contemplated by melanoma-specific repositories. The complex interaction between molecular players requires network-based approaches to suggest novel key intervention strategies, to stratify patients, and to individualize patient treatment. In section three, the dynamic changes in cell count of different melanoma cell types, immune cells, and fibroblasts are modeled and contemplated by stimulating or inhibiting effects between cells. Such cellular models represent another way to achieve therapy individualization and patient stratification. Section four leads to geometrical effects which will be augmented by the mechanics of melanoma in section five. Further aspects of oxygen, nutrient, and drug transport are presented in section six. The confined, spatial, and physiological tissue environment is relevant for tumor growth prognosis, drug delivery, surgery, and dermoscopic pattern recognition. All available computational melanoma models are listed in Supplemental Table 1 and organized, as shown in Figure 1.

2 Molecular networks

Molecular networks represent larger sets of molecules in an interconnected manner and go beyond the statistical significance of single features and the gene-set enrichment analysis paradigm [20]. Network science shows how biological functions emerge from the interactions between the components of living systems and how these emergent properties enable and constrain the behavior of those components [9]. In order to explore this rich information source, system biology provides frameworks tailored to each commonly known -omics data type. Melanoma-specific -omics data can be obtained from genomic [21, 22] and proteomic studies [23] but also from the secretome [24] or the metabolome [25, 26]. Because multiple -omics data are rarely integrated with a systems-centered approach [27], the following studies and repositories are only a starting point.

2.1 Repositories to inform network models

Published knowledge in the form of structured and centralized searchable databases facilitates model development. Beside general sources for system biologists [15], melanoma-specific databases are available (Table 1). The Melanoma Molecular Map Project (MMMP) is an open-access, participative project that structures published knowledge about molecules, genes, and pathways to enable translational perspectives [16]. The MelGene project provides an easily searchable database of genetic association studies of cutaneous melanoma, as well as a meta-analysis for many polymorphisms [17]. The MelanomaDB database lists published genomic datasets, including clinical and molecular information, and allows the creation of gene lists by merging selected studies [18]. The Melanoma Gene Database (MGDB) provides extensive entries about 527 melanoma-associated genes (422 protein-coding), including epigenetic and drug-related evidence [175]. Attention is required when using these databases, which accumulate data from multiple sources, sometimes in an automated manner, and are thus susceptible to perpetuate the biases and errors of the data source [19].

2.2 Models for melanoma genomics

The melanoma-specific repositories contain mainly genetic data with not yet fully identified patterns. The mutation pattern within the genome of metastatic melanoma can be used to find mutually exclusive gene modules [28]. If two proteins are related in an interaction network and their genes are mutated in a way that one gets amplified while the other gets deleted or only one gets modified without the other, one could presume that this happens to intensify cancer pathways at the protein level under given pathophysiological pressure. Consequently, one can conclude that a protein inhibits or activates the other in a known interaction network. The pathophysiologic pressure on cancer protein pathways selects mutation patterns with survival benefits. One analysis of The Cancer Genome Atlas (TCGA) melanoma samples integrated somatic mutations with copy number alterations and found concomitant deregulation of the G-protein and MAPK signaling pathways [29]. Similarly, integrated genomic and epigenomic analyses have been used to classify melanoma brain metastases in different mutually exclusive molecular subtypes [30].

2.3 Models for melanoma transcriptomics

The melanoma transcriptome is more context-specific than the genome and easier to measure than the proteome. The pattern changes can be used to stratify patients or to identify drug targets. Beyond this, they can give an impression of the re-wiring of pathways. Barter *et al.* applied three different strategies (single genes, gene sets, and network analysis) to 47 melanoma microarray datasets. They concluded that

network methods do not perform better overall, that these different approaches tend not to classify patients consistently, and that the optimal method might have to be identified patient-specifically [31]. Wang *et al.* performed 45 siRNA screens of the A375 cell line, whole-genome sequencing, and Bayesian gene network interference to enable directional and synergistic conclusions. Similar to Barter's findings, the network hubs alone were not sufficient to better stratify patients. However, if the network hubs are contextualized with cell-cycle and deoxyribonucleic acid (DNA)-repair function, the prediction of an individual prognosis is possible [32]. The concept of pathway re-wiring is based on the following reasoning. Some mutations can cause modified protein structures, which in turn can alter the links between the proteins without a change in protein concentration levels. Two proteins are interacting if the transcript level change of one protein correlates or anti-correlates with the transcript level change of another protein. When co-expression gets lost, the connection gets lost, and the connectivity reduces. When two unrelated proteins show a new co-expression in the next progression stage, the connectivity increases, and a pathway re-wiring can be assumed. This network analysis can be performed independent of significantly changed differential expression and fold changes. Kaushik *et al.* followed this strategy and meta-analyzed 632 melanoma microarray samples with melanoma progression stages: normal skin, non-metastatic (radial and vertical growth phase), metastatic, and lymph node metastases [33]. They diversified the clinical relevant groups by pooling the data of tissue samples with untreated and cisplatin-treated melanoma cell lines and melanocytes. The extracted re-wired pathway hubs were subsequently checked for drugability, which is important as many promising targets cannot be influenced pharmacologically [34].

2.4 Models for melanoma proteomics

The proteome directly mirrors cellular function. Genomic and transcriptomic data do not show the post-transcriptional, translational, and further epigenetic changes and are thus limited in their representation of final physical processes. Proteomic data is, *e.g.*, very beneficial for modeling the signal transduction such as MAPK or phosphatidylinositol-4,5-bisphosphate 3-kinase catalytic subunit alpha (PIK3CA) pathway [35]. In the context of melanoma, most studies aim either at understanding resistance mechanisms or at the responses to particular compounds.

For example, it was possible to predict with high accuracy the apoptosis susceptibility of 11 melanoma cell lines to TRAIL and dacarbazine (DTIC) using 17 protein measurements. This was achieved by grouping measurements in pathway-inspired functional groups and using these in multivariate statistical analysis [36]. Resistance in melanoma cell lines was studied with data-driven modeling and multivariate statistics. 21 phosphoproteins were measured over time in a panel of 10 cell lines subjected to different doses of five different RAF/MAP2K inhibitors [37]. This led to the identification of an early down-regulation of the mitogen-activated protein kinase 8 (MAPK8)[1]/jun proto-oncogene, AP-1 transcription factor subunit (JUN) pathway upon RAF/MAP2K inhibition, but an up-regulation in six cell lines at later time points. This study showed that a fraction of treated cells become quiescent and apoptosis-resistant. The same group further validated these results and suggested targeting MAPK8, protein tyrosine kinase 2 (PTK2), or SRC proto-oncogene, non-receptor tyrosine kinase (SRC) to inhibit this particular drug-resistant phenotype [38].

Bernardo-Faura *et al.* used Fuzzy Logic to investigate the temporal network re-wiring in A375 cells in response to different kinase inhibitors. The authors used a prior-knowledge network to simulate the behavior of the cells over time, and detected discrepancies at specific time-points between the model predictions and the measurements. This work, as well, underlines the importance of the MAPK8 pathway in early drug-induced changes in signaling pathways [39].

Del Mistro *et al.* studied the signaling network changes in phosphor-proteomic data due to underlying resistance of mutated BRAF melanoma cell lines to sublethal tumor necrosis factor related apoptosis inducing ligand (TRAIL) receptor-targeted agonist IZI1551. Systemic network analysis with Dynamic Bayesian modeling identified X-linked inhibitor of apoptosis (XIAP) and interleukin 21 (IκBα) as potential drug targets. Consequently, targeting these nodes in the subsequent experimental validation led to a re-sensitization of the cell lines [40].

Another comprehensive study is about the impact of MYC proto-oncogene, bHLH transcription factor (MYC) on the proteome and drug resistance, which lead to the identification of a co-targeting strategy [41].

2.5 Models for melanoma metabolomics

The metabolic state is the consequence of proteomic function and environmental conditions such as nutrient and oxygen shortages. Metabolite concentrations can be obtained with robust measurements, and well-established methods are available (Antoniewicz, 2015). Notably, Scott *et al.* used metabolic flux analysis to characterize the response of seven melanoma cell lines

[1] Also known as c-Jun N-terminal kinases JNK

to hypoxia [43]. They showed the crucial roles of both Warburg and Pasteur effects in melanoma and paved the way for the therapeutic targeting of metabolism. While the Pasteur effect describes reduced glycolysis with increased oxygen, the Warburg effect refers to cancer cells performing glycolysis despite the presence of oxygen [44]. Future studies might further combine metabolic modeling with other omics-data.

2.6 Mechanistic network models for melanoma

Completely validated mechanistic network models of melanoma seem not been published yet, but a valid Boolean model of melanogenesis covers both keratinocyte and melanocyte signaling. Lee *et al.*, thereby, imposed increasing ultraviolet B (UVB) light intensity and modeled the cellular response to it. The simulated profiles of the protein levels were individually compared to literature to check qualitative plausibility. Lee *et al.* demonstrated the central role of catenin beta 1 (CTNNB1) in the regulation of both melanogenesis and apoptosis. This prediction was then validated using UVB-exposed reconstituted human skin equivalents [45]. Moreover, a system of ordinary differential equations (ODE) was used to model the MAPK, PIK3CA/AKT serine/threonine kinase 1 (AKT1), and other pathways with 48 species and 48 biochemical reactions [46]. The model was an extension of the model of PC-12 (rat adrenal gland) cells and [47] shows that increasing dabrafenib concentrations cause declining pERK concentration but in unphysiological ranges. Future ODE-based modeling of melanoma signaling would ideally improve the balance between model size and melanoma-specific data to enable robust predictions. Sensitivity analyses and a model selection procedure might help to suggest key mechanisms and intervention strategies.

As described in this section, the network information can be used to stratify patients, to find druggable targets, and to understand the impact of therapy on the biochemical pathways. The next section describes models to inter-connect cells instead of molecules. Cell population models are used to find coherencies between cell culture and clinical patient populations or to understand the immune system at the whole-body level.

3 Cell population models: bridging cell culture to clinics

Melanoma cells are not isolated entities and interact with keratinocytes, fibroblasts, and immune cells. Moreover, melanoma cells might be divided into subtypes or phenotypes. Population models often describe the interaction between them, *e.g.*, how the abundance of one cell population influences the abundance of another cell population. A subset of these models integrate cell culture data; another subset of these models are experimentally adjusted with human or murine *in vivo* data.

3.1 Melanoma models can mimic the interplay of cell types

Flach *et al.* studied the interplay of melanoma cells, stromal fibroblasts, and stromal fibronectin. In their interpretation, free melanoma cells at the stromal interface activate fibroblasts to get mechanical support. The mechanically supported cells proliferate until they become blocked due to space limitations, albeit the space limitation is simplified to state values in this ODE network model [48]. Accordingly, several studies point to the crucial role of extracellular matrix (ECM) remodeling, fibronectin, and PTK2 signaling in driving resistance to BRAF inhibitors [49, 50]. This conceptual model of Flach *et al.* has been refined, validated, and extended to BRAFi and PTK2i therapy [51]. The results allowed a deeper understanding of the role of stroma during acquired resistance and its potential role during targeted therapy in drug-resistant patients [48, 51]. The same group worked on a dynamic autophagy model with AKT1i therapy for melanoma [52]. After cell culture and clinical patient data had been integrated into the autophagy model and key stratification parameters were identified. Stratification parameters could either accompany clinical trials or support treatment choice. Another melanoma cell population model is provided by Sun *et al.* with an excellent description of the parameter origin. The considered cell types are BRAFi sensitive, BRAFi resistant, and may or may not enter the metastatic cell state after the initiation of drug treatment. Cells grow until a maximum cell burden. The set of stochastic differential equations with 19 parameters is experimentally adjusted via circulating tumor cell DNA and melanoma cell line data. Progression-free survival is set equal with the melanoma cell concentration for simplicity [53], whereby more data might allow a more clinical relevant linkage between these two. Future models with integrated pharmacokinetic elements might consider clinically relevant pharmacokinetic models [54].

3.2 Cell interplay is studied for melanoma immunology

Cell population models for the interplay of melanoma cells with immune cells are helpful as melanomas are highly immunogenic tumors [55]. This high immunogenicity is the reason for the success of therapies based on immune activation in this tumor type. Indeed, melanoma was the first cancer type for which an immune checkpoint inhibitor and an oncolytic virus

were approved [56, 57]. As such, several computational models have been specially developed to study the interplay between immune and melanoma cells. For example, several ODE systems were devised to model melanoma with Th1 and Th2 helper lymphocytes [58], with natural killer cell (NK) cells in the context of interleukin 21 (IL21) therapy [59], with M1 and M2 macrophages [60], or both macrophages and helper lymphocytes [61]. Also, vaccine strategies based on dendritic cell therapy for melanoma were modeled with a multi-compartment ODE system to define adequate doses and schedules [62]. However, one drawback of these models is that the patients' intrinsic variables, key determinants in immune-related therapies, are not taken into account [63]. One study took into account the genetic signatures being associated with resistance to immunotherapies. The parameterized ODE model suggested co-adjuvants for successful vaccine therapies [64]. In another study, Pappalardo *et al.* implemented an on-grid cellular automaton model of melanoma, in which melanoma cells interact with macrophages, T cells, and dendritic cells in different cellular states. Pappalardo *et al.* highlighted the role of TNF receptor superfamily member 9 (TNFRSF9) for successful therapy and adjusted their model with experimental mice data of activated or resting OT1 T-cells and anti-TNFRSF9 antibodies in B16 melanoma [65]. Given the size of the model, additionally experimental data would further improve model parameterization and robustness [66].

In summary, cell-population models can combine clinical and cell culture data and might support the determination of an individualized drug regimen based on cellular dynamics. While these models are suitable for freely acting cells, tumors are frequently restricted by the ECM and anatomical space limitations. These effects were simplified by three models mentioned above [48, 65, 51]. While one refers to three-dimensional (3D) spheroid growth in collagen gel, two refer to tumor size in mice. Tumor growth is more complex and requires spatial, mechanical, and physiological characteristics being addressed in the following three sections.

4 Spatial models for melanoma

The spatial tumor expansion in tissue has played a subsidiary role heretofore. In the following, spatially distributed factors of lesion and environment are addressed. For instance, spatial patterns in dermoscopic pictures can be used to classify a particular lesion to obtain hints for prospective growth and the necessity of surgical intervention. Subsequently, combining cell-population models with geometry provide insights into the success of surgical therapy. When focussing on the cellular level, the collocation of cells can partly point to factors with the most control cell mass expansion. However, a more in-depth look at histological features of skin and other host tissues reveal that solely geometrical solutions may not be sufficient as mechanical cues significantly impact deformation and development.

4.1 Pattern recognition of melanoma

The pattern of naevi and melanoma *in situ* are the physical consequence of biochemical processes in the epidermis and are usually assessed and classified in dermatology to initiate early therapy. The related patterns can be modeled in two dimensions using a mixture theory model [101]. The study shows how different patterns of malignant cells can form within a healthy cell environment. two-dimensional (2D) patterns of naevi and melanoma can also be subjected to planar linear transformations using two subsequent dermoscopy pictures. Those pictures allow the classification of melanoma growth rates and naevi symmetry [141]. The ABCD criteria for melanoma have been mathematically considered too [142]. Automated optical classification of naevi and melanomas is a fast-growing field and employs machine learning methods for image recognition. The sensitivity and specificity of these models matched the decision quality of dermatologists [143, 144, 145]. Specific features in 2D dermoscopy pictures can also be used to determine the Breslow depth with specificity and sensitivity of almost 100%, which has direct prognostic value [146]. furthermore, the depth of invasion is an important prognostic marker for patient survival, and the Breslow index can be determined manually or automatically from histopathological images [144, 147].

4.2 Models of surgical treatment

Surgical treatment is the consequence of early identified melanomas. Wide excision of primary melanoma can have counter-intuitive ramifications according to the reaction-diffusion model of Eikenberry *et al.*. The surgical resection of primary melanomas might include tumor-associated immune cells, which lead to an accelerated outgrowth of local metastasis due to reduced immune suppression [148]. Computational models are also used to assist image-guided and computer-assisted surgery, mainly for the brain [149]. The brain, besides lung and lymph nodes, is a preferred host tissue for metastatic melanoma [75].

4.3 Dissecting parameters in spatial models is a challenge

Fully experimentally validated models of melanoma expansion are still limited to simple Petri dish experiments. In a series of reports, Treloar *et al.* use a

lattice cellular automaton model and an experimental approach to identify different parameters of MM127 colony growth where cell motility, cell–to–cell adhesion, and cell proliferation influence the same: the expansion of the cell colony [67, 68]. These parameters were also estimated using a Bayesian framework coupled with a stochastic model of 2D melanoma growth [69]. Using melanoma and fibroblast monocultures as well as different co-culture systems, Haridas *et al.* have parameterized a partial differential equations (PDE) model of the interactions of cancer cells and fibroblasts [70]. Continuous modeling of melanoma cells under different osmotic pressures was performed with a 2D lattice model to simulate scratch assays [71]. The aim was to distinct migration/invasion between primary and metastatic cells. New vertex modeling strategies [72] and scratch assay analysis tools [73] might further improve this approach.

4.4 Spatial organization of skin and confined spaces

The previously described spatial parameter determination strategy for cell lines is especially helpful for the epidermal skin layer. However, the skin is more complex and also contains irregular fibrous tissue beneath the epidermal layer separated by a collagenous basement membrane [74]. At the dermal-epidermal junction, keratinocytes are generated and migrate through the epidermis up to the skin surface, where they keratinize to the stratified protective barrier called stratum corneum. The epidermal layer is also the most common location for melanoma initiation. Residing melanocytes can become benign neoplasms and appear as innate or acquired naevi [75]. Further changes and appearing atypical cells constitute the first malignant stage: the radial growth phase. From the clinical perspective and the perspective of modeling, the basement membrane is crucial. Invasion through the basement membrane indicates the vertical growth phase, which may require adjuvant therapy besides surgical treatment. Pharmacological therapy is indicated for metastatic growth in secondary tissues. In contrast to the epidermis, the dermis layer is streaked with collagen and elastin fibers synthesized by fibroblasts [76], and these ECM fibers restrict tumor expansion [77]. Using colony growth in 2D cell culture experiments does not lead to quantitative parameters for spatial models representing stromal processes. For example, migration velocity depends on the ECM fiber geometry [78], the migration process is fundamentally different in confined structures [79], and depend on the paxillin (PXN) and transforming growth factor beta 1 induced transcript 1 (TGFB1I1) balance related to PTK2 [80]. Moreover, BRAF inhibition promotes matrix metalloproteinases (MMP) activity and cell migration in three dimensions [81]. A consequent experimental parameterization of realistic melanoma growth models is difficult to find and is aggravated by the diversity of parameter origin and their mutual dependency, as shown by Treloar *et al.* [67, 68]. The modeling of the tumor microenvironment has to consider additional factors like extracellular matrix stiffness and topography, oxygen and nutrients gradients, and interstitial fluid pressure [162].

5 Mechanical models of melanoma

Mechanical cues in the environment influence directly important biochemical cancer pathways and have a complex impact on tumour progression [87, 14]. Consequently mechanical models become more attention and three methods will be presented in the following such as mixture theory, the thermodynamically constrained averaging theory (TCAT), and the discrete ansatz with cross-linked elastic cells. These three methodologies can mimic the growth in tissues, while a tissue without any malignant contortions is already a complex modelling task [107]. As the integration and measurement of mechanical cues is not yet widely used, a summary of experimental methods is given.

5.1 Impact of mechanoregulation

In three dimensions, additional factors impair drug sensitivity [49, 82] and increase or decrease the tumor growth rate [83]. The stromal environment causes non-genetic phenotype switches between proliferative and mesenchymal stages [84, 85], and environmental melanoma-associated fibroblasts are suspected of playing an essential role in melanoma progression [86]. Fibroblast activity is closely linked to ECM and thus biomechanics, which is now recognized as a central pillar of tumor progression and metastasis [87, 14]. Mechanical melanoma models consider the growth-induced deformation of the ECM rich environment. The more the proliferating mass expands, the more counterforce is generated by the connected ECM fibers. The elastic energy is conserved and geometry dependent [77]. The mechanical deformation of tissues and mechanical stress influence intracellular signaling by mechano-sensors like PTK2 [88] or YY1 associated protein 1 (YY1AP1)/tafazzin (TAZ) [89], which are discussed as drug resistance mechanism for BRAF-mutant melanoma cells [38, 49, 90] or progression marker for cutaneous and G protein subunit alpha q (GNAQ) mutant uveal melanoma [91, 92, 93]. Proximity to mechano-regulating fibroblasts can induce pathway changes to PIK3CA/mechanistic target of rapamycin kinase (MTOR) and switch the phenotype of melanoma cells to the mesenchymal state [94]. Consequently, melanoma cells reduce the inherent stiffness to facilitate invasion [95, 96]. However, our knowledge of mechanosensitive pathways is far from complete [97, 98, 99, 96], and mechanical phenomenons

require computational models to comprehend.
Additionally, the skin, being the primary site for cutaneous melanoma, is a mechano-sensitive organ. Skin can grow when it is stretched, and rete ridges, projections of the epidermis into the dermis, were recently suspected to form according to mechanical characteristics [76]. The skin has inspired many computational models describing dermal transport processes as well as providing a mechanical understanding of the skin's optical, functional, and structural characteristics [100].

5.2 Mixture theory

Two mixture theory models exist. One describes the skin surface, and one mimics the vertical section [101, 102]. Balois *et al.* consider interstitial fluid pressure, a mechanically optimal cell density, and friction between the melanocytic lesion and the surrounding. Ciarletta *et al.* represent melanoma in the radial growth phase in the ECM free epidermis as a viscous fluid sliding on a basement membrane with friction dependent growth velocity. In a second step, this friction is neglected and instead considered between the basement membrane and an additional keratinocyte representing fluid. Melanoma cell and keratinocyte fluid are adjacent to each other, and the tumor front between them is a moving interface/ free boundary problem subjected to stability analysis.

5.3 TCAT theory

TCAT models [103] represent a multi-phase approach, which is different from mixture theory and circumvents the free boundary problem. TCAT models do not have a defined tumor boundary at the macro-scale, but the ECM spans the whole tissue, with a higher concentration at the basement membrane. The interstitial fluid, the healthy, and the malignant cells squeeze via local rules through the solid but deformable porous ECM network. Averaging of local properties causes a macroscale behavior that resembles the distortion of the tissue and the invasion of the basement membrane. By adjusting the cancer cell plasticity but not ECM integrity, the model changes from solid to invasive growth [104].

5.4 Disordered lattice model

The discrete model [105] describes individual cells on a 2D disordered lattice. Cells are represented as spheres, which are connected via breakable springs. The springs mimic ECM and cell-cell contacts. Melanoma induced MMP activity is modeled by a higher probability of spring breaking near melanoma cells. Despite the simple mechanical and geometrical laws, the simulation results give a realistic impression. Because discrete models are more computationally demanding than continuous models, they allow only limited upscaling. However, the benefit of this single-cell modeling approach is the potential discrimination between compressive and tensile stress, which can differ strongly across the ECM biopolymer types [106] and tumor locations [77]. The model by Taloni *et al.* was validated with 2D experiments. The experiments were performed under osmotic pressure without fibronectin, which is an important linker between mechanics and intracellular signaling.

5.5 Experimental methods for mechanical melanoma models

Although modeling promises to become more and more prominent in melanoma research, and continuous improvements in computational power make more complex and realistic models accessible, experimentally validated parameterization remains a crucial bottleneck. To produce high-quality mathematical models, quantitative data under standardized operating procedures are required [150, 151].
Tumor spheres and spheroids in general [152], and organotypic *in vitro* models for melanoma [153] in particular offer more realistic experimental conditions. Fully functional organotypic skin constructs [154] can mimic all melanoma progression stages. 3D constructs are not only a carrier of cells; they modify the experimental outcome. Thus, quantification of the hydrogel system parameter, such as the shear or Young's modulus becomes standard. The shear modulus G of the gel system, or the roughly three times higher Young's modulus E, is stated with the unit kPa ($E=2G(1+\nu)$; ν: Poisson's ratio) [49, 98, 88]. Knowing the impact of mechanical cues in the modeling process prevents common data integration problems. For example, the frequently used matrigel for invasion assays has an elastic modulus of 0.45 kPa and is consequently a weaker obstacle than the basement membrane reaching 250-500 kPa [155, 156]. Additionally, the impact of stress relaxation should be not underestimated as it has a decisive impact on further development [157]. A range of hydrogel systems is available [158] and can also be used for automated drug testing [159] albeit questions of standardisation of 3D cell culture models remain [160].
Mechanical parameters are difficult to measure and span up to 5 log steps depending on tissue moisture and experimental setting [108]. Experimental mechano-sensors enable the measurement of submolecular force transmissions [109], and fluorescent oil microdroplets allow the measurement of anisotropic stress fields in 3D tissues [110]. Tunable alginate microcapsules can be used to determine the mechanical growth-pressure of spheroids [111], and high throughput mechanical testing of cells is possible with optical deformation of cells [112]. If the direct measurement of stiffness is not possible, the lamin A/C (LMNA) to

lamin B1/2 (LMNB1/2) ratio serves as an appropriate biomarker [113].
The clinical imaging technology elastography gives direct access to the tissue stiffness fields and thus tumor locations *in vivo* [114]. Elastography can also be used for the *in vivo* staging of melanoma [115]. The integration of elastography and melanoma mechano-signaling could highlight stiff areas where mechano-sensors influence melanoma pathways. This could facilitate the translation of these research models into clinically relevant predictive models.
The complexity of tumor cell environment interactions requires a step-wise understanding with a multitude of experimental techniques [161] and related computational efforts (Figure 1). Computational scientists must incorporate the experimental context to develop meaningful computational melanoma progression models.

6 Transport of oxygen and drugs

Several models describe how oxygen and drugs move from the source to a melanocytic lesion as the presence of oxygen and nutrients control the viability of cancerous and healthy tissues. Thereby, multiple oxygen sources as well as vascular and pericellular transport routes matter as described in the following.

6.1 Oxygenation of melanoma in skin and brain

Impaired oxygen and nutrient delivery cause necrotic cores, which is a widespread assumption. A necrotic core can be modelled explicitly [103] or indirectly via nutrient concentration reduction [102]. However, Thibaut Balois & Martine Ben Amar question the existence of necrotic cores in epidermal melanoma and take the atmospheric oxygen source into account [116, 101]. If oxygen came only from the dermal vasculature, the oxygen partial pressure would drop to around eight mmHg at the skin surface [117]. Mild hypoxic conditions are present around the basement membrane, promoting melanocyte proliferation [118] as well as melanoma progression [119]. Interestingly, the brain, a common location for metastasized melanoma, has also a low tissue oxygen concentrations reaching 35 mmHg [120].

6.2 Experimental aspects of oxygen

Most established melanoma cell lines are cultured under atmospheric oxygen and are therefore evolutionarily adjusted to these artificial conditions. Molecular oxygen sensors for 3D settings [121] are as possible as advanced hypoxia sensors [122] to improve the validation of computational models. Oxygen consumption rates of cells can be obtained with the Seahorse technology [123] and were determined for melanocytes and melanoma cell lines [124].

6.3 Models of melanoma-associated vascularization

The tumor-associated vascularization is influenced by oxygen limitations and mechanical cues [125, 126]. Mathematical blood vessel models define an independent computational research field [127]. Notably, Welter and Rieger combined the discrete modeling of vasculature remodeling with the continuous gradients of melanoma cells, oxygen, nutrients, and drugs [128]. They used melanoma-specific data for the vasculature [129]. This excellent model is useful to simulate blood flow and to study the impact of space limitations on simple drug diffusion and nutrient supply. Wang *et al.* created an agent-based model with both melanoma and endothelial cells with a focus on angiogenesis. They tested the combined effect of doxorubicin chemotherapy and kinase insert domain receptor (KDR)[2] inhibition with sunitinib [130]. It might be interesting to see a follow-up model with improved use of biological data for parameter, synergy, and validation. Dzwinel *et al.* coupled several continuous submodels of melanoma growth to increase modeling quality and efficiency. They used a single phase continuum for growth accompanied by angiogenesis, vascular remodeling, and tumor ECM interactions. The model was embedded in a realistic virtual skin structure, and the melanoma progression resembled nodular, lentigo maligna, and acral lentiginous melanoma [131]. The same group extended the model by a discrete vascularization dynamic, which was coupled intermittently [132]. The used approach, called "super-modeling" by the authors, is a theory on model synchronization [133]. However, the connection coefficients seem untrained in comparison to non-biological application areas, and the coupling remains weak [133]. This modeling group is very active in melanoma, refines the model continuously, and also uses particle automata models to produce visually realistic models [134, 135].
In general, while these models provide valuable insight into the vasculature, much work is needed to ensure adequate melanoma-specific parametrizations and validations. Einar Rofstads' group provides excellent data sets on melanoma-associated vascularization and might be considered for further modeling projects [136].

6.4 Drug delivery models

Blood vessels are an essential route for drugs to the location of action, and pharmacokinetics is studied to determine the drug concentration in local blood plasma. However, the transport from the blood vessels or skin surface to the melanoma cells depends on the diffusion

[2] Also known as vascular endothelial growth factor receptor 2 (VEGFR)

coefficient of the microanatomical structure. Drug delivery models are available for both the penetration of spherical tumors with melanin-binding antibodies for radioimmunotherapy [137] and SPACE-EGF mediated transdermal delivery of MYC siRNA [138]. However, the impact of biomechanics and tumor physiology on drug delivery is not considered by those models but discussed for MU89 Melanoma in mice [139, 140]. A proliferating mass makes fibrous tissue crowded and compressed. This might lead to a reduced interstitial fluid volume fraction and thus impaired drug transport. Such a phenomenon might be best modeled with the multi-phase flow in porous media [103].

7 Discussion

The creation of a mechanistic and predictive model is a serious and work-intensive endeavor that forces all participants to think deeper [9]. Ultimately, the reward is more aim-tailored research but also the discovery of hidden causalities, which would otherwise have rendered explorative research inconclusive or contradictory. Recent progress in devising experimental procedures for parameter determination has fueled the work of several computational groups. Conversely, certain phenomena can only be understood with computational methods, such as computational mechanics. Mathematical modeling of melanoma presents several specificities ranging from the high mutation load and cell plasticity to oxygen uptake at the skin surface. Nevertheless, most of the current models of melanoma are not yet sufficiently adapted to the requirements in biology and medicine. The recurring problems in almost all reviewed research can be expressed in four challenges and are discussed accordingly.

7.1 First challenge: tumor heterogenity

The first challenge is the biochemical heterogeneity. The high mutation load, signaling network plasticity, and cell line heterogeneity makes the fitting of mechanistic ODE systems or straightforward network inference from patients' biopsies difficult. Instead, most studies focus on well-characterized cell line collections to carefully extract specific regulatory network motifs with multivariate statistics. The cell line-specific models are suitable for modeling and understanding drug testing. Notable works used systems biology to investigate the impact of new compounds such as TRAIL [36, 40], while others focus on identifying potential targets by perturbating the biological system with several kinase inhibitors [39, 37].

7.2 Second challenge: melanoma type specifity

The second challenge is melanoma type specific modeling. Melanocytic tumors occur in various forms at different locations and are based on different etiologies [163]. A few important types are lentigo maligna melanoma, superficial spreading melanoma, and acral lentiginous melanoma. Nonetheless, computational papers often refer to a general term of melanoma, albeit each melanoma type can substantially differ in treatment, environment, and growth pattern. In computational biology, mechanistic links between growth patterns and melanoma-type specific biochemical markers could prospectively find the same importance as in pathology [75, 163]. In contrast to constructing models around a few abstract mathematical parameters and retrospectively allocate histopathological sections to a given simulation outcome, modelers might emphasize the pathological causality and relevant biochemical root-causes leading to a melanoma-type specific growth outcome. A deeper examination of cancer pathology, anatomy, and physiology might also prevent unjustified assumptions. Some authors set initial lesions at positions, where they rarely project from such as the epidermal stratum corneum, albeit the stratum basale is often the location of initial lesions [163]. The unique oxygen patterns in skin [116, 117], the tendency of melanocytes to proliferate better in mild hypoxic conditions [118], the strong oxygen consumption of melanoma cells [124], or the importance of driver mutations in this highly mutated cancer type [21, 22] are further factors, which might find more consideration by modelers of melanoma. Not all concepts, model structures, and parameters can be ingenuously taken from models of other cancer types. Future melanoma models might represent more melanoma type specific characteristics and parameters, whereby attention should also be drawn to the respective histopathology and the host tissue in which the simulated melanoma is thought to be simulated. Eventually, context and tissue-specific modeling of certain melanoma types is more insightful than generic cancer or melanoma models.

7.3 Third challenge: complexity

The third challenge is the appropriate level of complexity as neither very small and simple nor extensive models can deliver reliable predictions. Models that are as simple as possible are the gold standard in modeling, as perfectly shown by Kim or Picco *et al.* [52, 51]. However, if models neglect major effects, or the remaining model elements are too abstract to be interpreted, the result will be of little use. For example, careful work was performed to determine mutually dependent parameters of a cell colony [70]. However, the impact of the mechanical environment on these parameter values [14], such as migration [78], exacerbates the transfer of these parameters to complex 3D models.

On the contrary, especially large scale models which have been set-up [46, 130] will benefit from sufficient and appropriate melanoma-specific data, to further increase the validity of their conclusions. The same can be observed in physical oncology. Mixture theory allows easier models and fewer parameters. Still, it is difficult to measure abstract parameters or to biologically interpret the equations as they pool too many biological sub-systems to homogeneous entities. In contrast, Sciumé *et al.* accurately differentiates between cells, fibrous compounds, and interstitial fluid. This makes experimental parameter determination easier and aligns better with medical and biological lines of thinking. However, the model requires many parameters, which must still be biologically characterized. Best interdisciplinary communication is reached with agent-based models, were cells are separately depicted. However, the computational demand for simulating individual cells is substantial. More experience is necessary to find the right level of complexity that can be of practical use and allow both computationally feasible and biologically sound models.

7.4 Fourth challenge: correct data integration

The fourth challenge is the accurate integration of evidence. Experimental fact and assumed fiction are difficult to distinguish in many publications, and it is of little use if an extensive biological section is written independently of the computational part but is hardly reflected by the equations at the end [102]. To enable scrutiny by melanoma experts and to facilitate evidence-based model extensions or improvements, it seems necessary that each element of a model structure is biologically explained, interpreted, and referenced only within the degree of factual implementation, even if this requires extensive supplemental information. The reasoning behind modeling decisions should be accountable. At least, the behavior of all system elements might be checked for plausibility as done in one work [45].

Besides the verifiability of the model structure, parameters are very ambiguous in most papers, and only a few papers provided supplemental information about data extraction and conversion [53]. Unfortunately, many publications work with uncurated parameter lists, and interested readers are recommended to trace back parameter values to the primary source to judge the validity. We found that data indicated as melanoma-specific were based on other diseases and tissue origins such as glioblastoma or the adrenal gland. Other estimated or assumed parameters are referenced in subsequent papers as if they are experimentally determined values. Moreover, the context of experimental origin often do not fit the intended model context, or whole parameter sets are normalized in an original paper and then carried over several computational paper generations regardless of studied biology. In order to bring models closer to biological evidence, parameters should be referenced only to the original experimental publications, and information should be given on the experimental context and potential parameter conversions.

Most likely, not all required data will be available, but transparency on evidence is generally lacking. It remains to be debated to which extend a model must contain melanoma-specific data to be considered a melanoma model or how close a model must match medical evidence to be seen as a valuable contribution to melanoma research.

7.5 Lack of interdisciplinary is the root cause

The four challenges reflect the most persistent problem of melanoma-specific modeling: interdisciplinarity. Computational models require close collaboration between experimental, clinical, and computational scientists in an iterative procedure. Modeling generates hypotheses, which can be tested *in vitro*, and experimental results inform the design of better models and allow the falsification of theories [164]. However, a general problem in the interdisciplinary work in biology and medicine is that the more demanding the necessary mathematical and physical framework becomes, the more disconnected it becomes from the experimental and theoretical knowledge in biology, medicine, and pharmacology. On the one hand, computational groups cannot reproduce and test the diverse parameter sources in their labs, lacking the time and expertise to embrace the whole complexity of biological relationships and experimental methods. On the other hand, biologists and clinicians find it difficult to help, as the more developed computational procedures are likewise difficult to comprehend. Therefore, better quality standards between and in both computational [165] and biomedical research [166] need to be developed and adopted. A more sophisticated way might be the stepwise model development accompanied by advanced cell culture strategies, as suggested by Figure 1. The gap between the different disciplines is not closed yet, which leads to conceptual problems in the models and inappropriate parameter choices.

Conclusion

Cancer is a highly complex, heterogeneous disease, characterized by a series of genetic, metabolic, and functional changes at the cellular and tissue level [167]. Melanoma-specific dynamics along tumor progression stages in both plasticity [95] and genetics [168] highlight the need for integrative models to better understand disease mechanisms of melanoma. The model-building community works across different scales and

comprises studies centered on signaling pathways and gene regulation [15], metabolism [169, 170], epithelial tissue mechanics [171], tumor physiology [172], or the immune system [173]. Four challenges for computational melanoma models have been discussed:

- Melanoma heterogeneity,
- Melanoma-type specificity,
- The balance between simplicity and thoroughness, and
- Melanoma data integration and evidence.

Consequently, interdisciplinarity and clinical relevance remain a bottleneck if it comes to the practical use of melanoma-specific systems biology and physical oncology models. However, if all disciplines improve interdisciplinary collaboration, the future promises us an unmatched insight.

Declarations

Ethics approval and consent to participate
Not applicable

Consent for publication
Not applicable

Availability of data and material
Not applicable

Competing interests
The authors declare that they have no competing interests.

Funding
We would like to acknowledge the European Union's Horizon 2020 research and innovation programme under the Marie Skłodowska-Curie grant agreement No 642295 and this work was supported by the Luxembourg National Research Fund (FNR) within the projects MelanomSensitivity (BMBF/BM/7643621).

Author's contributions
PL made crucial initial contributions to the manuscript, especially for systems biology and databases. MA collected the remaining literature for cell population and spatial models with related experimental approaches. DK provided the papers on melanoma and the experimental conditions. DK and TS ensured the quality, completeness, and scientific soundness of the review. All authors contributed substantially to the manuscript as well as critically read and corrected the final version.

Acknowledgements
The authors thank Sébastien De Landtsheer for the fruitful discussions and the critical reading of the manuscript.

Author details
[1]Systems Biology Group, Life Science Research Unit, University of Luxembourg, 6, avenue du Swing, 4367 Belval, Luxembourg.
[2]Experimental Dermatology, Department of Dermatology, Dresden University of Technology, Fetscherstraße 105, 01307 Dresden, Germany.

References

1. Karimkhani C, Green AC, Nijsten T, Weinstock M, Dellavalle RP, Naghavi M, et al. The global burden of melanoma: results from the global burden of disease study 2015. British Journal of Dermatology. 2017;177(1):134–140.
2. Kunz M. Oncogenes in melanoma: an update. European journal of cell biology. 2014;93(1-2):1–10.
3. Omholt K, Platz A, Kanter L, Ringborg U, Hansson J. NRAS and BRAF mutations arise early during melanoma pathogenesis and are preserved throughout tumor progression. Clinical Cancer Research. 2003;9(17):6483–6488.
4. Aris M, Barrio MM. Combining immunotherapy with oncogene-targeted therapy: a new road for melanoma treatment. Frontiers in immunology. 2015;6:46.
5. Amaral T, Sinnberg T, Meier F, Krepler C, Levesque M, Niessner H, et al. MAPK pathway in melanoma part II—secondary and adaptive resistance mechanisms to BRAF inhibition. European journal of cancer. 2017;73:93–101.
6. Amaral T, Sinnberg T, Meier F, Krepler C, Levesque M, Niessner H, et al. The mitogen-activated protein kinase pathway in melanoma part I–activation and primary resistance mechanisms to BRAF inhibition. European journal of cancer. 2017;73:85–92.
7. Sharma P, Hu-Lieskovan S, Wargo JA, Ribas A. Primary, adaptive, and acquired resistance to cancer immunotherapy. Cell. 2017;168(4):707–723.
8. Ugurel S, Röhmel J, Ascierto PA, Flaherty KT, Grob JJ, Hauschild A, et al. Survival of patients with advanced metastatic melanoma: the impact of novel therapies–update 2017. European Journal of Cancer. 2017;83:247–257.
9. Wolkenhauer O. Why model? Frontiers in physiology. 2014;5:21.
10. Weinberg RA. Coming full circle–from endless complexity to simplicity and back again. Cell. 2014;157(1):267–271.
11. Kolch W, Halasz M, Granovskaya M, Kholodenko BN. The dynamic control of signal transduction networks in cancer cells. Nature Reviews Cancer. 2015;15(9):515.
12. Mitchell MJ, Jain RK, Langer R. Engineering and physical sciences in oncology: challenges and opportunities. Nature Reviews Cancer. 2017;17(11):659.
13. Hatzikirou H, Chauviere A, Bauer AL, Leier A, Lewis MT, Macklin P, et al. Integrative physical oncology. Wiley Interdisciplinary Reviews: Systems Biology and Medicine. 2012;4(1):1–14.
14. Ju RJ, Stehbens SJ, Haass NK. The Role of Melanoma Cell-Stroma Interaction in Cell Motility, Invasion, and Metastasis. Frontiers in Medicine. 2018;5.
15. Werner HM, Mills GB, Ram PT. Cancer Systems Biology: a peek into the future of patient care? Nature reviews Clinical oncology. 2014;11(3):167.
16. Mocellin S, Rossi CR. The melanoma molecular map project. LWW; 2008.
17. Antonopoulou K, Stefanaki I, Lill CM, Chatzinasiou F, Kypreou KP, Karagianni F, et al. Updated field synopsis and systematic meta-analyses of genetic association studies in cutaneous melanoma: the MelGene database. Journal of Investigative Dermatology. 2015;135(4):1074–1079.
18. Trevarton AJ, Mann M, Knapp C, Araki H, Wren JD, Stones-Havas S, et al. MelanomaDB: a web tool for integrative analysis of melanoma genomic information to identify disease-associated molecular pathways. Frontiers in oncology. 2013;3:184.
19. Reinhold WC. Commentary on "MelanomaDB: a web tool for integrative analysis of melanoma genomic information to identify disease-associated molecular pathways". Frontiers in genetics. 2013;4:156.
20. Subramanian A, Tamayo P, Mootha VK, Mukherjee S, Ebert BL, Gillette MA, et al. Gene set enrichment analysis: a knowledge-based approach for interpreting genome-wide expression profiles. Proceedings of the National Academy of Sciences. 2005;102(43):15545–15550.
21. Akbani R, Akdemir KC, Aksoy BA, Albert M, Ally A, Amin SB, et al. Genomic classification of cutaneous melanoma. Cell. 2015;161(7):1681–1696.
22. Loftus SK. The next generation of melanocyte data: Genetic, epigenetic, and transcriptional resource datasets and analysis tools. Pigment cell & melanoma research. 2018;31(3):442–447.
23. Mittal P, Jain M. Proteomics: An indispensable tool for novel biomarker identification in melanoma. J Data Mining Genomics Proteomics. 2016;7(204):2153–0602.
24. Liberato T, Pessotti DS, Fukushima I, Kitano ES, Serrano SM, Zelanis A. Signatures of protein expression revealed by secretome analyses of cancer associated fibroblasts and melanoma cell lines. Journal of proteomics. 2018;174:1–8.
25. Fischer GM, Vashisht Gopal Y, McQuade JL, Peng W, DeBerardinis RJ, Davies MA. Metabolic strategies of melanoma cells: Mechanisms,

interactions with the tumor microenvironment, and therapeutic implications. Pigment cell & melanoma research. 2018;31(1):11–30.
26. Ratnikov BI, Scott DA, Osterman AL, Smith JW, Ronai ZA. Metabolic rewiring in melanoma. Oncogene. 2017;36(2):147.
27. Emmert-Streib F, Glazko G, De Matos Simoes R, et al. Statistical inference and reverse engineering of gene regulatory networks from observational expression data. Frontiers in genetics. 2012;3:8.
28. Ciriello G, Cerami E, Sander C, Schultz N. Mutual exclusivity analysis identifies oncogenic network modules. Genome research. 2012;22(2):398–406.
29. Guan J, Gupta R, Filipp FV. Cancer systems biology of TCGA SKCM: efficient detection of genomic drivers in melanoma. Scientific reports. 2015;5:7857.
30. Marzese DM, Scolyer RA, Roqué M, Vargas-Roig LM, Huynh JL, Wilmott JS, et al. DNA methylation and gene deletion analysis of brain metastases in melanoma patients identifies mutually exclusive molecular alterations. Neuro-oncology. 2014;16(11):1499–1509.
31. Barter RL, Schramm SJ, Mann GJ, Yang YH. Network-based biomarkers enhance classical approaches to prognostic gene expression signatures. BMC systems biology. 2014;8(4):S5.
32. Wang L, Hurley DG, Watkins W, Araki H, Tamada Y, Muthukaruppan A, et al. Cell cycle gene networks are associated with melanoma prognosis. PloS one. 2012;7(4):e34247.
33. Kaushik A, Bhatia Y, Ali S, Gupta D. Gene network rewiring to study melanoma stage progression and elements essential for driving melanoma. PloS one. 2015;10(11):e0142443.
34. Dang CV, Reddy EP, Shokat KM, Soucek L. Drugging the'undruggable'cancer targets. Nature Reviews Cancer. 2017;17(8):502.
35. Saez-Rodriguez J, MacNamara A, Cook S. Modeling signaling networks to advance new cancer therapies. Annual review of biomedical engineering. 2015;17:143–163.
36. Passante E, Würstle ML, Hellwig CT, Leverkus M, Rehm M. Systems analysis of apoptosis protein expression allows the case-specific prediction of cell death responsiveness of melanoma cells. Cell death and differentiation. 2013;20(11):1521.
37. Fallahi-Sichani M, Moerke NJ, Niepel M, Zhang T, Gray NS, Sorger PK. Systematic analysis of BRAFV600E melanomas reveals a role for JNK/c-Jun pathway in adaptive resistance to drug-induced apoptosis. Molecular systems biology. 2015;11(3):797.
38. Fallahi-Sichani M, Becker V, Izar B, Baker GJ, Lin JR, Boswell SA, et al. Adaptive resistance of melanoma cells to RAF inhibition via reversible induction of a slowly dividing de-differentiated state. Molecular systems biology. 2017;13(1):905.
39. Bernardo-Faura M, Massen S, Falk CS, Brady NR, Eils R. Data-derived modeling characterizes plasticity of MAPK signaling in melanoma. PLoS computational biology. 2014;10(9):e1003795.
40. Del Mistro G, Lucarelli P, Müller I, De Landtsheer S, Zinoveva A, Hutt M, et al. Systemic network analysis identifies XIAP and IκBα as potential drug targets in TRAIL resistant BRAF mutated melanoma. NPJ systems biology and applications. 2018;4(1):39.
41. Korkut A, Wang W, Demir E, Aksoy BA, Jing X, Molinelli EJ, et al. Perturbation biology nominates upstream–downstream drug combinations in RAF inhibitor resistant melanoma cells. Elife. 2015;4:e04640.
42. Levesque MP, Dummer R, Beerenwinkel N. Perturbing resistance: a network perspective. Pigment cell & melanoma research. 2015;1(29):5–7.
43. Scott DA, Richardson AD, Filipp FV, Knutzen CA, Chiang GG, Ze'ev AR, et al. Comparative metabolic flux profiling of melanoma cell lines beyond the warburg effect. Journal of Biological Chemistry. 2011;286(49):42626–42634.
44. Koppenol WH, Bounds PL, Dang CV. Otto Warburg's contributions to current concepts of cancer metabolism. Nature Reviews Cancer. 2011;11(5):325.
45. Lee HS, Goh MJ, Kim J, Choi TJ, Lee HK, Na YJ, et al. A systems-biological study on the identification of safe and effective molecular targets for the reduction of ultraviolet B-induced skin pigmentation. Scientific reports. 2015;5:10305.
46. Pappalardo F, Russo G, Candido S, Pennisi M, Cavalieri S, Motta S, et al. Computational modeling of PI3K/AKT and MAPK signaling pathways in melanoma cancer. PLoS One. 2016;11(3):e0152104.
47. Brown KS, Hill CC, Calero GA, Myers CR, Lee KH, Sethna JP, et al. The statistical mechanics of complex signaling networks: nerve growth factor signaling. Physical biology. 2004;1(3):184.
48. Flach EH, Rebecca VW, Herlyn M, Smalley KS, Anderson AR. Fibroblasts contribute to melanoma tumor growth and drug resistance. Molecular pharmaceutics. 2011;8(6):2039–2049.
49. Hirata E, Girotti MR, Viros A, Hooper S, Spencer-Dene B, Matsuda M, et al. Intravital imaging reveals how BRAF inhibition generates drug-tolerant microenvironments with high integrin β1/FAK signaling. Cancer cell. 2015;27(4):574–588.
50. Fedorenko IV, Abel EV, Koomen JM, Fang B, Wood ER, Chen YA, et al. Fibronectin induction abrogates the BRAF inhibitor response of BRAF V600E/PTEN-null melanoma cells. Oncogene. 2016;35(10):1225.
51. Picco N, Sahai E, Maini PK, Anderson AR. Integrating models to quantify environment-mediated drug resistance. Cancer research. 2017;77(19):5409–5418.
52. Kim E, Rebecca VW, Smalley KS, Anderson AR. Phase i trials in melanoma: A framework to translate preclinical findings to the clinic. European Journal of Cancer. 2016;67:213–222.
53. Sun X, Bao J, Shao Y. Mathematical modeling of therapy-induced cancer drug resistance: connecting cancer mechanisms to population survival rates. Scientific reports. 2016;6:22498.
54. Ouellet D, Gibiansky E, Leonowens C, O'Hagan A, Haney P, Switzky J, et al. Population pharmacokinetics of dabrafenib, a BRAF inhibitor: effect of dose, time, covariates, and relationship with its metabolites. The Journal of Clinical Pharmacology. 2014;54(6):696–706.
55. Herzberg B, Fisher DE. Metastatic melanoma and immunotherapy. Clinical Immunology. 2016;172:105–110.
56. Hodi FS, O'day SJ, McDermott DF, Weber RW, Sosman JA, Haanen JB, et al. Improved survival with ipilimumab in patients with metastatic melanoma. New England Journal of Medicine. 2010;363(8):711–723.
57. Andtbacka R, Kaufman HL, Collichio F, Amatruda T, Senzer N, Chesney J, et al. Talimogene laherparepvec improves durable response rate in patients with advanced melanoma. J clin Oncol. 2015;33(25):2780–2788.
58. Kogan Y, Agur Z, Elishmereni M. A mathematical model for the immunotherapeutic control of the Th1/Th2 imbalance in melanoma. Discrete & Continuous Dynamical Systems-Series B. 2013;18(4).
59. Cappuccio A, Elishmereni M, Agur Z. Cancer immunotherapy by interleukin-21: potential treatment strategies evaluated in a mathematical model. Cancer Research. 2006;66(14):7293–7300.
60. den Breems NY, Eftimie R. The re-polarisation of M2 and M1 macrophages and its role on cancer outcomes. Journal of theoretical biology. 2016;390:23–39.
61. Eftimie R, Hamam H. Modelling and investigation of the CD4+ T cells–Macrophages paradox in melanoma immunotherapies. Journal of theoretical biology. 2017;420:82–104.
62. DePillis L, Gallegos A, Radunskaya A. A model of dendritic cell therapy for melanoma. Frontiers in oncology. 2013;3.
63. Pizzurro GA, Barrio MM. Dendritic cell-based vaccine efficacy: aiming for hot spots. Frontiers in immunology. 2015;6:91.
64. Santos G, Nikolov S, Lai X, Eberhardt M, Dreyer FS, Paul S, et al. Model-based genotype-phenotype mapping used to investigate gene signatures of immune sensitivity and resistance in melanoma micrometastasis. Scientific reports. 2016;6:24967.
65. Pappalardo F, Forero IM, Pennisi M, Palazon A, Melero I, Motta S. SimB16: modeling induced immune system response against B16-melanoma. PloS one. 2011;6(10):e26523.
66. Altrock PM, Liu LL, Michor F. The mathematics of cancer: integrating quantitative models. Nature Reviews Cancer. 2015;15(12):730.
67. Treloar KK, Simpson MJ, Binder BJ, McElwain DS, Baker RE. Assessing the role of spatial correlations during collective cell spreading. Scientific reports. 2014;4:5713.
68. Treloar KK, Simpson MJ, Haridas P, Manton KJ, Leavesley DI, McElwain DS, et al. Multiple types of data are required to identify the mechanisms influencing the spatial expansion of melanoma cell colonies. BMC systems biology. 2013;7(1):137.

69. Vo BN, Drovandi CC, Pettitt AN, Pettet GJ. Melanoma cell colony expansion parameters revealed by approximate Bayesian computation. PLoS computational biology. 2015;11(12):e1004635.
70. Haridas P, Penington CJ, McGovern JA, McElwain DS, Simpson MJ. Quantifying rates of cell migration and cell proliferation in co-culture barrier assays reveals how skin and melanoma cells interact during melanoma spreading and invasion. Journal of theoretical biology. 2017;423:13–25.
71. La Porta CA, Ghilardi A, Pasini M, Laurson L, Alava MJ, Zapperi S, et al. Osmotic stress affects functional properties of human melanoma cell lines. The European Physical Journal Plus. 2015;130(4):64.
72. Barton DL, Henkes S, Weijer CJ, Sknepnek R. Active vertex model for cell-resolution description of epithelial tissue mechanics. PLoS computational biology. 2017;13(6):e1005569.
73. Stichel D, Middleton AM, Müller BF, Depner S, Klingmüller U, Breuhahn K, et al. An individual-based model for collective cancer cell migration explains speed dynamics and phenotype variability in response to growth factors. NPJ systems biology and applications. 2017;3(1):5.
74. Breitkreutz D, Koxholt I, Thiemann K, Nischt R. Skin basement membrane: the foundation of epidermal integrity—BM functions and diverse roles of bridging molecules nidogen and perlecan. BioMed research international. 2013;2013.
75. Shain AH, Bastian BC. From melanocytes to melanomas. nature reviews Cancer. 2016;16(6):345.
76. Evans ND, Oreffo RO, Healy E, Thurner PJ, Man YH. Epithelial mechanobiology, skin wound healing, and the stem cell niche. Journal of the mechanical behavior of biomedical materials. 2013;28:397–409.
77. Nia HT, Liu H, Seano G, Datta M, Jones D, Rahbari N, et al. Solid stress and elastic energy as measures of tumour mechanopathology. Nature Biomedical Engineering. 2016;1:0004.
78. Tozluoğlu M, Tournier AL, Jenkins RP, Hooper S, Bates PA, Sahai E. Matrix geometry determines optimal cancer cell migration strategy and modulates response to interventions. Nature cell biology. 2013;15(7):751.
79. Paul CD, Mistriotis P, Konstantopoulos K. Cancer cell motility: lessons from migration in confined spaces. Nature reviews Cancer. 2017;17(2):131.
80. Deakin NO, Turner CE. Distinct roles for paxillin and Hic-5 in regulating breast cancer cell morphology, invasion, and metastasis. Molecular biology of the cell. 2011;22(3):327–341.
81. Leight JL, Tokuda EY, Jones CE, Lin AJ, Anseth KS. Multifunctional bioscaffolds for 3D culture of melanoma cells reveal increased MMP activity and migration with BRAF kinase inhibition. Proceedings of the National Academy of Sciences. 2015;112(17):5366–5371.
82. Shao Y, Aplin AE. Akt3-mediated resistance to apoptosis in B-RAF–targeted melanoma cells. Cancer research. 2010;p. 0008–5472.
83. Ambrosi D, Pezzuto S, Riccobelli D, Stylianopoulos T, Ciarletta P. Solid tumors are poroelastic solids with a chemo-mechanical feedback on growth. Journal of Elasticity. 2017;129(1-2):107–124.
84. Levesque MP, Cheng PF, Raaijmakers MI, Saltari A, Dummer R. Metastatic melanoma moves on: translational science in the era of personalized medicine. Cancer and Metastasis Reviews. 2017;36(1):7–21.
85. Hoek KS, Eichhoff OM, Schlegel NC, Döbbeling U, Kobert N, Schaerer L, et al. In vivo switching of human melanoma cells between proliferative and invasive states. Cancer Res. 2008;68(3):650–656.
86. Izar B, Joyce CE, Goff S, Cho NL, Shah PM, Sharma G, et al. Bidirectional cross talk between patient-derived melanoma and cancer-associated fibroblasts promotes invasion and proliferation. Pigment cell & melanoma research. 2016;29(6):656–668.
87. Hutchenreuther J, Leask A. Why target the tumor stroma in melanoma? Journal of cell communication and signaling. 2018;12(1):113–118.
88. Paszek MJ, Zahir N, Johnson KR, Lakins JN, Rozenberg GI, Gefen A, et al. Tensional homeostasis and the malignant phenotype. Cancer cell. 2005;8(3):241–254.
89. Halder G, Dupont S, Piccolo S. Transduction of mechanical and cytoskeletal cues by YAP and TAZ. Nature reviews Molecular cell biology. 2012;13(9):591.
90. Kim MH, Kim J, Hong H, Lee SH, Lee JK, Jung E, et al. Actin remodeling confers BRAF inhibitor resistance to melanoma cells through YAP/TAZ activation. The EMBO journal. 2016;35(5):462–478.
91. Feng X, Degese MS, Iglesias-Bartolome R, Vaque JP, Molinolo AA, Rodrigues M, et al. Hippo-independent activation of YAP by the GNAQ uveal melanoma oncogene through a trio-regulated rho GTPase signaling circuitry. Cancer cell. 2014;25(6):831–845.
92. Sanchez IM, Aplin AE. Hippo: hungry, hungry for melanoma invasion. Journal of Investigative Dermatology. 2014;134(1):14–16.
93. Nallet-Staub F, Marsaud V, Li L, Gilbert C, Dodier S, Bataille V, et al. Pro-invasive activity of the Hippo pathway effectors YAP and TAZ in cutaneous melanoma. Journal of Investigative Dermatology. 2014;134(1):123–132.
94. Seip K, Fleten KG, Barkovskaya A, Nygaard V, Haugen MH, Engesæter BØ, et al. Fibroblast-induced switching to the mesenchymal-like phenotype and PI3K/mTOR signaling protects melanoma cells from BRAF inhibitors. Oncotarget. 2016;7(15):19997.
95. Weder G, Hendriks-Balk MC, Smajda R, Rimoldi D, Liley M, Heinzelmann H, et al. Increased plasticity of the stiffness of melanoma cells correlates with their acquisition of metastatic properties. Nanomedicine. 2014;10(1):141–148.
96. Wei SC, Yang J. Forcing through tumor metastasis: the interplay between tissue rigidity and epithelial–mesenchymal transition. Trends in cell biology. 2016;26(2):111–120.
97. Ringer P, Colo G, Faessler R, Grashoff C. Sensing the mechano-chemical properties of the extracellular matrix. Matrix Biology. 2017;64:6–16.
98. Northey JJ, Przybyla L, Weaver VM. Tissue force programs cell fate and tumor aggression. Cancer discovery. 2017;.
99. Charras G, Yap AS. Tensile forces and mechanotransduction at cell–cell junctions. Current Biology. 2018;28(8):R445–R457.
100. Querleux B. Computational biophysics of the skin. vol. 10. Jenny Stanford Publishing; 2014.
101. Balois T, Amar MB. Morphology of melanocytic lesions in situ. Scientific reports. 2014;4:3622.
102. Ciarletta P, Foret L, Ben Amar M. The radial growth phase of malignant melanoma: multi-phase modelling, numerical simulations and linear stability analysis. Journal of the Royal Society Interface. 2010;8(56):345–368.
103. Sciumè G, Santagiuliana R, Ferrari M, Decuzzi P, Schrefler B. A tumor growth model with deformable ECM. Physical biology. 2014;11(6):065004.
104. **Albrecht** t, Sciumè G, Lucarelli P, Sauter T. Thermodynamically constrained averaging theory for cancer growth modelling. IFAC-PapersOnLine. 2016;49(26):289–294.
105. Taloni A, Alemi AA, Ciusani E, Sethna JP, Zapperi S, La Porta CA. Mechanical properties of growing melanocytic nevi and the progression to melanoma. PloS one. 2014;9(4):e94229.
106. Pritchard RH, Huang YYS, Terentjev EM. Mechanics of biological networks: from the cell cytoskeleton to connective tissue. Soft matter. 2014;10(12):1864–1884.
107. Lanir Y. Multi-scale structural modeling of soft tissues mechanics and mechanobiology. Journal of Elasticity. 2017;129(1-2):7–48.
108. Derler S, Gerhardt LC. Tribology of skin: review and analysis of experimental results for the friction coefficient of human skin. Tribology Letters. 2012;45(1):1–27.
109. Grashoff C, Hoffman BD, Brenner MD, Zhou R, Parsons M, Yang MT, et al. Measuring mechanical tension across vinculin reveals regulation of focal adhesion dynamics. Nature. 2010;466(7303):263.
110. Campàs O, Mammoto T, Hasso S, Sperling RA, O'connell D, Bischof AG, et al. Quantifying cell-generated mechanical forces within living embryonic tissues. Nature methods. 2014;11(2):183.
111. Alessandri K, Sarangi BR, Gurchenkov VV, Sinha B, Kießling TR, Fetler L, et al. Cellular capsules as a tool for multicellular spheroid production and for investigating the mechanics of tumor progression in vitro. Proceedings of the National Academy of Sciences. 2013;110(37):14843–14848.
112. Otto O, Rosendahl P, Mietke A, Golfier S, Herold C, Klaue D, et al. Real-time deformability cytometry: on-the-fly cell mechanical phenotyping. Nature methods. 2015;12(3):199.

113. Swift J, Ivanovska IL, Buxboim A, Harada T, Dingal PDP, Pinter J, et al. Nuclear lamin-A scales with tissue stiffness and enhances matrix-directed differentiation. Science. 2013;341(6149):1240104.
114. Kim W, Ferguson VL, Borden M, Neu CP. Application of elastography for the noninvasive assessment of biomechanics in engineered biomaterials and tissues. Annals of biomedical engineering. 2016;44(3):705–724.
115. Jid CB, Bolboacă SD, Cosgarea R, Şenilă S, Rogojan L, Lenghel M, et al. Doppler ultrasound and strain elastography in the assessment of cutaneous melanoma: preliminary results. Medical ultrasonography. 2015;17(4):509–514.
116. Stücker M, Struk A, Altmeyer P, Herde M, Baumgärtl H, Lübbers DW. The cutaneous uptake of atmospheric oxygen contributes significantly to the oxygen supply of human dermis and epidermis. The Journal of physiology. 2002;538(3):985–994.
117. Wang W, Winlove C, Michel C. Oxygen partial pressure in outer layers of skin of human finger nail folds. The Journal of physiology. 2003;549(3):855–863.
118. Horikoshi T, Balin AK, Carter DM. Effects of oxygen tension on the growth and pigmentation of normal human melanocytes. Journal of investigative dermatology. 1990;96(6):841–844.
119. Hanna SC, Krishnan B, Bailey ST, Moschos SJ, Kuan PF, Shimamura T, et al. HIF1α and HIF2α independently activate SRC to promote melanoma metastases. J Clin Invest. 2013;123(5):2078–2093.
120. Carreau A, Hafny-Rahbi BE, Matejuk A, Grillon C, Kieda C. Why is the partial oxygen pressure of human tissues a crucial parameter? Small molecules and hypoxia. Journal of cellular and molecular medicine. 2011;15(6):1239–1253.
121. Dmitriev RI, Borisov SM, Düssmann H, Sun S, Müller BJ, Prehn J, et al. Versatile conjugated polymer nanoparticles for high-resolution O2 imaging in cells and 3D tissue models. ACS nano. 2015;9(5):5275–5288.
122. Erapaneedi R, Belousov VV, Schäfers M, Kiefer F. A novel family of fluorescent hypoxia sensors reveal strong heterogeneity in tumor hypoxia at the cellular level. The EMBO journal. 2016;35(1):102–113.
123. Brand MD, Nicholls DG. Assessing mitochondrial dysfunction in cells. Biochemical Journal. 2011;435(2):297–312.
124. Hall A, Meyle KD, Lange MK, Klima M, Sanderhoff M, Dahl C, et al. Dysfunctional oxidative phosphorylation makes malignant melanoma cells addicted to glycolysis driven by the V600EBRAF oncogene. Oncotarget. 2013;4(4):584.
125. Chwalek K, Tsurkan MV, Freudenberg U, Werner C. Glycosaminoglycan-based hydrogels to modulate heterocellular communication in in vitro angiogenesis models. Scientific reports. 2014;4:4414.
126. Balcioglu HE, Van De Water B, Danen EH. Tumor-induced remote ECM network orientation steers angiogenesis. Scientific reports. 2016;6:22580.
127. Scianna M, Bell C, Preziosi L. A review of mathematical models for the formation of vascular networks. Journal of theoretical biology. 2013;333:174–209.
128. Welter M, Rieger H. Physical determinants of vascular network remodeling during tumor growth. The European Physical Journal E. 2010;33(2):149–163.
129. Döme B, Paku S, Somlai B, Tímár J. Vascularization of cutaneous melanoma involves vessel co-option and has clinical significance. The Journal of pathology. 2002;197(3):355–362.
130. Wang J, Zhang L, Jing C, Ye G, Wu H, Miao H, et al. Multi-scale agent-based modeling on melanoma and its related angiogenesis analysis. Theoretical Biology and Medical Modelling. 2013;10(1):41.
131. Dzwinel W, Kłusek A, Vasilyev OV. Supermodeling in simulation of melanoma progression. Procedia Computer Science. 2016;80:999–1010.
132. Łoś M, Paszyński M, Kłusek A, Dzwinel W. Application of fast isogeometric L2 projection solver for tumor growth simulations. Computer Methods in Applied Mechanics and Engineering. 2017;316:1257–1269.
133. Duane GS, Grabow C, Selten F, Ghil M. Introduction to focus issue: Synchronization in large networks and continuous media—data, models, and supermodels. AIP Publishing; 2017.
134. Kłusek A, Łoś M, Paszyński M, Dzwinel W. Efficient model of tumor dynamics simulated in multi-GPU environment. The International Journal of High Performance Computing Applications. 2019;33(3):489–506.
135. Panuszewska M, Minch B, Wcisło R, Dzwinel W. PAM: Discrete 3-D Model of Tumor Dynamics in the Presence of Anti-tumor Treatment. In: International Conference on Cellular Automata. Springer; 2018. p. 42–54.
136. Gaustad JV, Simonsen TG, Leinaas MN, Rofstad EK. Sunitinib treatment does not improve blood supply but induces hypoxia in human melanoma xenografts. BMC cancer. 2012;12(1):388.
137. Schweitzer AD, Rakesh V, Revskaya E, Datta A, Casadevall A, Dadachova E. Computational model predicts effective delivery of 188-Re-labeled melanin-binding antibody to metastatic melanoma tumors with wide range of melanin concentrations. Melanoma research. 2007;17(5):291–303.
138. Liu J, Ding W, Ruan R, Zou L, Chen M, Wei P, et al. A theoretical study on inhibition of melanoma with controlled and targeted delivery of siRNA via skin using SPACE-EGF. Annals of biomedical engineering. 2017;45(6):1407–1419.
139. Stylianopoulos T, Martin JD, Chauhan VP, Jain SR, Diop-Frimpong B, Bardeesy N, et al. Causes, consequences, and remedies for growth-induced solid stress in murine and human tumors. Proceedings of the National Academy of Sciences. 2012;109(38):15101–15108.
140. Ramírez-Torres A, Rodríguez-Ramos R, Merodio J, Penta R, Bravo-Castillero J, Guinovart-Díaz R, et al. The influence of anisotropic growth and geometry on the stress of solid tumors. International Journal of Engineering Science. 2017;119:40–49.
141. Mendes AI, Nogueira C, Pereira J, Fonseca-Pinto R. On the geometric modulation of skin lesion growth: a mathematical model for melanoma. Research on Biomedical Engineering. 2016;32(1):44–54.
142. Lee H, Kwon K. A mathematical analysis of the ABCD criteria for diagnosing malignant melanoma. Physics in Medicine & Biology. 2017;62(5):1865.
143. Esteva A, Kuprel B, Novoa RA, Ko J, Swetter SM, Blau HM, et al. Dermatologist-level classification of skin cancer with deep neural networks. Nature. 2017;542(7639):115.
144. Haenssle H, Fink C, Schneiderbauer R, Toberer F, Buhl T, Blum A, et al. Man against machine: diagnostic performance of a deep learning convolutional neural network for dermoscopic melanoma recognition in comparison to 58 dermatologists. Annals of Oncology. 2018;29(8):1836–1842.
145. Marchetti MA, Codella NC, Dusza SW, Gutman DA, Helba B, Kalloo A, et al. Results of the 2016 international skin imaging collaboration international symposium on biomedical imaging challenge: comparison of the accuracy of computer algorithms to dermatologists for the diagnosis of melanoma from dermoscopic images. Journal of the American Academy of Dermatology. 2018;78(2):270–277.
146. Satheesha T, Satyanarayana D, Prasad MG, Dhruve KD. Melanoma is skin deep: a 3D reconstruction technique for computerized dermoscopic skin lesion classification. IEEE journal of translational engineering in health and medicine. 2017;5:1–17.
147. Xu H, Wang H, Berendt R, Jha N, Mandal M. Computerized measurement of melanoma depth of invasion in skin biopsy images. In: 2017 IEEE EMBS International Conference on Biomedical & Health Informatics (BHI). IEEE; 2017. p. 17–20.
148. Eikenberry S, Thalhauser C, Kuang Y. Tumor-immune interaction, surgical treatment, and cancer recurrence in a mathematical model of melanoma. PLoS computational biology. 2009;5(4):e1000362.
149. Payan Y. Soft tissue biomechanical modeling for computer assisted surgery. vol. 11. Springer; 2012.
150. Silk D, Kirk PD, Barnes CP, Toni T, Stumpf MP. Model selection in systems biology depends on experimental design. PLoS computational biology. 2014;10(6):e1003650.
151. Stanford NJ, Wolstencroft K, Golebiewski M, Kania R, Juty N, Tomlinson C, et al. The evolution of standards and data management practices in systems biology. Molecular systems biology. 2015;11(12):851.
152. Weiswald LB, Bellet D, Dangles-Marie V. Spherical cancer models in tumor biology. Neoplasia. 2015;17(1):1–15.
153. Kulms D, Meier F. In vitro models of melanoma. In: Skin Tissue Models. Elsevier; 2018. p. 57–75.

154. Vörsmann H, Groeber F, Walles H, Busch S, Beissert S, Walczak H, et al. Development of a human three-dimensional organotypic skin-melanoma spheroid model for in vitro drug testing. Cell death & disease. 2013;4(7):e719.
155. Halfter W, Oertle P, Monnier CA, Camenzind L, Reyes-Lua M, Hu H, et al. New concepts in basement membrane biology. The FEBS journal. 2015;282(23):4466–4479.
156. Soofi SS, Last JA, Liliensiek SJ, Nealey PF, Murphy CJ. The elastic modulus of Matrigel^{TM} as determined by atomic force microscopy. Journal of structural biology. 2009;167(3):216–219.
157. Chaudhuri O, Gu L, Klumpers D, Darnell M, Bencherif SA, Weaver JC, et al. Hydrogels with tunable stress relaxation regulate stem cell fate and activity. Nature materials. 2016;15(3):326.
158. Caliari SR, Burdick JA. A practical guide to hydrogels for cell culture. Nature methods. 2016;13(5):405–414.
159. Rimann M, Angres B, Patocchi-Tenzer I, Braum S, Graf-Hausner U. Automation of 3D cell culture using chemically defined hydrogels. Journal of laboratory automation. 2014;19(2):191–197.
160. Verjans ET, Doijen J, Luyten W, Landuyt B, Schoofs L. Three-dimensional cell culture models for anticancer drug screening: Worth the effort? Journal of cellular physiology. 2018;233(4):2993–3003.
161. Peela N, Truong D, Saini H, Chu H, Mashaghi S, Ham SL, et al. Advanced biomaterials and microengineering technologies to recapitulate the stepwise process of cancer metastasis. Biomaterials. 2017;133:176–207.
162. Holle AW, Young JL, Spatz JP. In vitro cancer cell–ECM interactions inform in vivo cancer treatment. Advanced drug delivery reviews. 2016;97:270–279.
163. Elder DE, Massi D, Scolyer RA, Willemze R. WHO classification of skin tumours. International Agency for Research on Cancer; 2018.
164. Howard J. Quantitative cell biology: the essential role of theory. Molecular biology of the cell. 2014;25(22):3438–3440.
165. Waltemath D, Wolkenhauer O. How modeling standards, software, and initiatives support reproducibility in systems biology and systems medicine. IEEE Transactions on Biomedical Engineering. 2016;63(10):1999–2006.
166. Begley CG, Ioannidis JP. Reproducibility in science: improving the standard for basic and preclinical research. Circulation research. 2015;116(1):116–126.
167. Hanahan D, Weinberg RA. Hallmarks of cancer: the next generation. cell. 2011;144(5):646–674.
168. Shain AH, Yeh I, Kovalyshyn I, Sriharan A, Talevich E, Gagnon A, et al. The genetic evolution of melanoma from precursor lesions. New England Journal of Medicine. 2015;373(20):1926–1936.
169. Antoniewicz MR. Methods and advances in metabolic flux analysis: a mini-review. Journal of industrial microbiology & biotechnology. 2015;42(3):317–325.
170. Pacheco MP, Sauter T. The FASTCORE family: for the fast reconstruction of compact context-specific metabolic networks models. In: Metabolic Network Reconstruction and Modeling. Springer; 2018. p. 101–110.
171. Brodland GW. How computational models can help unlock biological systems. In: Seminars in cell & developmental biology. vol. 47. Elsevier; 2015. p. 62–73.
172. Cristini V, Koay E, Wang Z. An Introduction to physical oncology: how mechanistic mathematical modeling can improve cancer therapy outcomes. CRC Press; 2017.
173. Konstorum A, Vella AT, Adler AJ, Laubenbacher RC. Addressing current challenges in cancer immunotherapy with mathematical and computational modelling. Journal of The Royal Society Interface. 2017;14(131):20170150.
174. Chatzinasiou F, Lill CM, Kypreou K, Stefanaki I, Nicolaou V, Spyrou G, et al. Comprehensive field synopsis and systematic meta-analyses of genetic association studies in cutaneous melanoma. Journal of the National Cancer Institute. 2011;103(16):1227–1235.
175. Zhang D, Zhu R, Zhang H, Zheng CH, Xia J. MGDB: a comprehensive database of genes involved in melanoma. Database. 2015;2015.

List of abbreviations

All biomolecule names in this thesis are approved by the HUGO Gene Nomenclature Committee (HGNC) of the Human Genome Organisation (HUGO).

**2D**	two-dimensional
**3D**	three-dimensional
**AKT1**	AKT serine/threonine kinase 1
**BRAF**	b-Raf proto-oncogene
**CTLA4**	cytotoxic T-lymphocyte associated protein 4
**CTNNB1**	catenin beta 1
**DTIC**	dacarbazine
**DNA**	deoxyribonucleic acid
**ECM**	extracellular matrix
**GNAQ**	G protein subunit alpha q
**HGNC**	HUGO Gene Nomenclature Committee
**HIF1A**	hypoxia inducible factor 1 subunit alpha
**HUGO**	Human Genome Organisation
**IκBα**	interleukin 21
**IL21**	interleukin 21
**JUN**	jun proto-oncogene, AP-1 transcription factor subunit
**KDR**	kinase insert domain receptor
**LMNA**	lamin A/C
**LMNB1/2**	lamin B1/2
**MAP2K**	mitogen-activated protein kinase kinases
**MAPK1**	mitogen-activated protein kinase 1
**MAPK8**	mitogen-activated protein kinase 8
**MGDB**	Melanoma Gene Database
**MMMP**	Melanoma Molecular Map Project
**MMP**	matrix metalloproteinases
**MTOR**	mechanistic target of rapamycin kinase
**MYC**	MYC proto-oncogene, bHLH transcription factor
**NR1I2**	NR1I2 nuclear receptor subfamily 1 group I member 2
**NK**	natural killer cell
**NRAS**	NRAS proto-oncogene, GTPase
**ODE**	ordinary differential equations
**PDCD1**	programmed cell death 1
**PDE**	partial differential equations
**PIK3CA**	phosphatidylinositol-4,5-bisphosphate 3-kinase catalytic subunit alpha
**PTEN**	phosphatase and tensin homolog
**PTK2**	protein tyrosine kinase 2
**PXN**	paxillin
**RAS**	RAS type GTPase family
**SRC**	SRC proto-oncogene, non-receptor tyrosine kinase
**TAZ**	tafazzin
**TCAT**	thermodynamically constrained averaging theory
**TCGA**	The Cancer Genome Atlas
**TRAIL**	tumor necrosis factor related apoptosis inducing ligand
**TGFB1I1**	transforming growth factor beta 1 induced transcript 1
**TNFRSF9**	TNF receptor superfamily member 9
**UVB**	ultraviolet B
**XIAP**	X-linked inhibitor of apoptosis
**YY1AP1**	YY1 associated protein 1

Figures

Figure 1 Computational and experimental approaches to understand cancer. Experimental approaches span from 2D cell culture to clinical data and are often correlated directly. Possible intermediate steps can delineate the response of cells to certain characteristics of the environment. Cells on gel sense the rigidity of the substratum, spheroids in hanging drops can develop a necrotic core, spheroid growing in alginate capsules reveal the growth pressure at which the capsule burst, spheroids in gel reveal the cellular response to a confined environment, spheroids in a tissue construct shows interactions with fibroblasts and host cells in a confined environment, and organotypic constructs and histological sections emphasize the behavior in a realistic anatomical structure. **Computational models** change accordingly in scale and approach. Methods are classified counter-clockwise, beginning at the top left corner. *Descriptive methods of statistics and bioinformatics* focus on the identification of single features. Often groups are compared, or the explanatory power of certain factors is investigated. *Systems biologists* increasingly connect different elements, focus on network information, and study dynamic effects. The network topology in steady-state is the first step but can also be extended to time dynamic and directed interactions. The networks might be compartmentalized to study communication across different cells, but the cells themselves can also represent network nodes, which is common in immunological studies. If interconnections between cells, with or without ECM, are studied and spatially distributed, on-grid and off-grid cellular automatons, vertex models, and reaction-diffusion models become relevant. Deformed tissue structures and anatomical obstacles require the integration of mechanical information. The more the approaches move from cell data to clinical images, the more pattern recognition becomes relevant. The functioning of the blood vessel system often depends on the pattern of the vessel network. Clinical images, such as from dermoscopy, might be linked via artificial intelligence to various pathologies. At the top right, computational methods of pharmacokinetics and pharmacodynamics relate drug dose to the concentration in blood plasma and then to the mode of action. The upper half of the figure pronounce the statistical significance; the bottom half of the figure shows models, which pronounce the importance of physical and mechanistic dependencies. In conclusion, a direct correlation between *in vitro* and *in vivo* data might be straight-forward, but might be also too simplistic. The laborious indirect way with step-wise experimental and computational extension of knowledge might be harder and more expensive, but more insightful in the long term and can enrich meaningful model development.

Additional Files

Supplementary Table 1:
Melanoma model overview. Appreviation: C=cellular, CP= cell population, T=tissue, O=organ, PP=patient population, 0D-3D : zero to three dimensional.

Table 1 Data bases containing melanoma data.

Databases	Information	Last update	Source
Melanoma Molecular Map Project	Information about single molecules molecular profiles and molecular pathways involved in melanoma progression	2015	[16]
MelGene	83,343 CM cases and 187,809 controls and reported on 1,114 polymorphisms in 280 different genes	2016	[17, 174]
MelanomaDB	Published melanoma genomic datasets including clinical and molecular information	20 May 2013	[18]
Melanoma Gene Database	Relationship between melanoma protein-coding genes, microRNAs and lncRNAs	02 Nov 2016	[175]

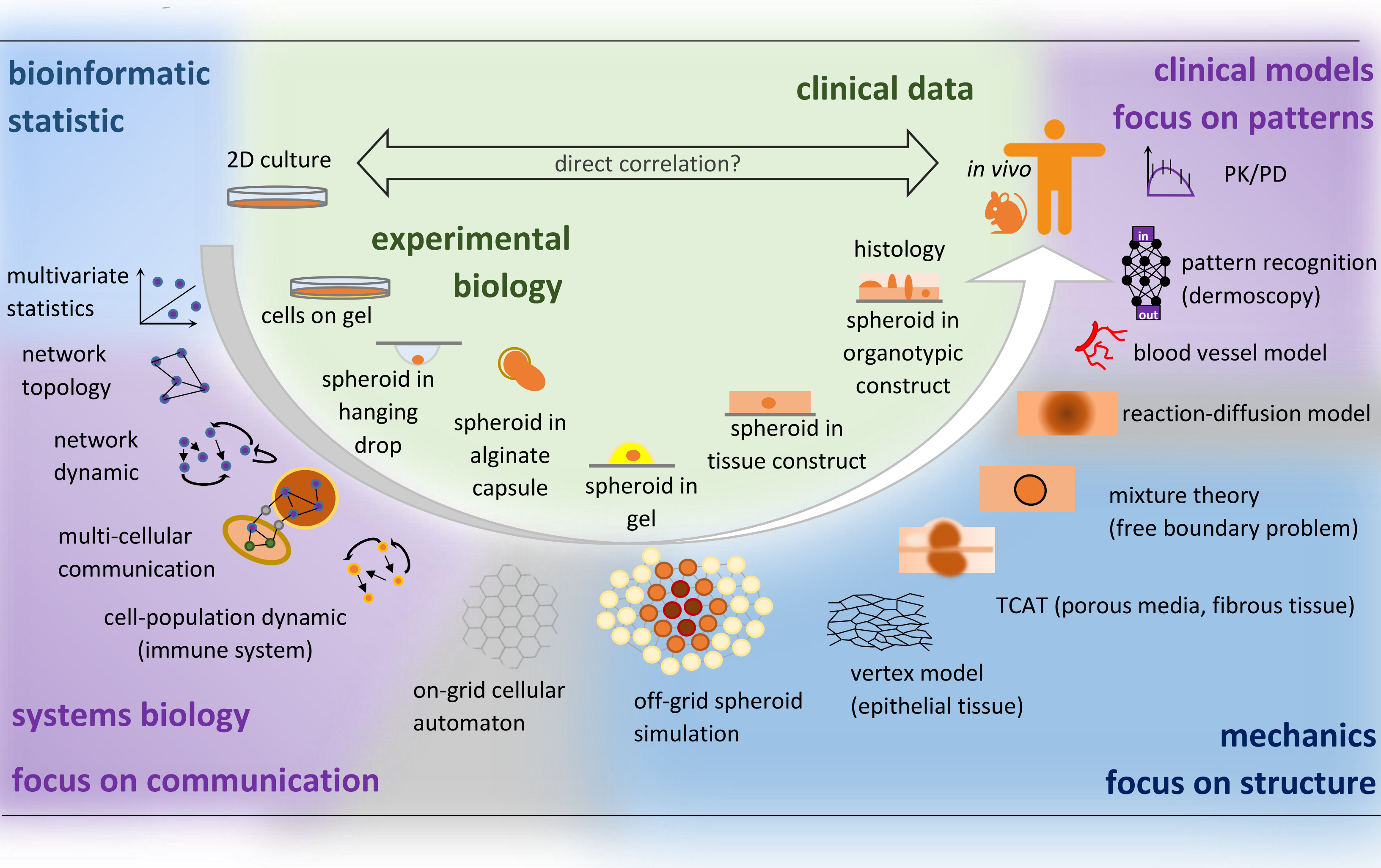

bioinformatic
statistic
clinical data
clinical models
focus on patterns
2D culture
direct correlation?
in vivo
PK/PD
experimental
biology
histology
pattern recognition
(dermoscopy)
in
out
multivariate
statistics
cells on gel
spheroid in
organotypic
construct
blood vessel model
network
topology
spheroid in
hanging
drop
spheroid in
alginate
capsule
reaction-diffusion model
network
dynamic
spheroid in
tissue construct
spheroid in
gel
mixture theory
(free boundary problem)
multi-cellular
communication
cell-population dynamic
(immune system)
TCAT (porous media, fibrous tissue)
on-grid cellular
automaton
off-grid spheroid
simulation
vertex model
(epithelial tissue)
systems biology
focus on communication
mechanics
focus on structure

Databases:	Source	Available data/information	updated
**Melanoma Molecular Map Project**	Mocellin and Rossi, 2008	single molecules, molecular profiles, molecular pathways	2010
**MelGene**	Antonopoulou et al., 2014; Chatzinasiou et al., 2011	192 Studies, 1114 polymorphisms and 280 different genes, 79 meta analysis 79.	2014
**MelanomaDB**	Trevarton et al., 2013	genes	2013
**Melanoma Gene Database**	Zhang et al., 2015	mutation,interaction, pathway, drug, expression, methylation	2016

	Source	Model type	Special player	aim	treatment	Exp. source and validation	scales	spatial	mechanical	Network coupling
network	Guan et al., 2015	Searches network motives in mutations	Deregulation of melanogenesis by G-protein and cyclin pathway			Cancer Genome Atlas (TCGA),303 metastatic melanomas	T	0D	no	yes
	Barter et al., 2014	Searches network motives in transcriptome				GSE53118	T	0D	no	yes
	Kaushik et al., 2015	Pathway rewiring in trascriptome				Divers microarrays of tissues and cell lines with or without treatmen	C, T	0D	no	yes
	Scott et al. 2011	Metabolic flux balance analysis	Actate, Alanine, Glycine, Proline, Fumarate, Serine, Malate, Aspartate, Glutamate, Citrate,Lactate, Pyruvate, Alanine	Warburg and Pasteur effect	normoxia, hypoxia, U-13C-glucose	WM35, Mel501, UACC903, WM793, Lu1205, MeWo	C	0D	no	yes
	Passante et al., 2013	Multivariate statistical analysis with network fragments	apoptosis signaling (BCL, XIAP)	Accurate cell death susceptibility determination	TRAIL, dacarbazine	PM-WK, RPM-MC, RPM-EP, MM-RU, MM-AN, MM-LH, A375, MeWo, SK-Mel-30, IGR-3	C	0D	no	yes
	Fallahi-Sichani et al., 2015	Multivariate statistical analysis , partial least square regression	JNK/cJUN, pS6, Kip1, Histone H3, PARP, Bim, NFKB, HSP27, p-38, cJun, AMPK, mTor, AKT, p90R	Understand drug resistance	PLX4720, Vemurafenib, SB590885, AZ628, AZD6244	C32, COLO858, MZ7MEL, MMACSF, RVH421, SKMEL28, WM115, WM1552C, PLX472	C	0D	no	yes
	Bernardo-Faura et al., 2014	Fuzzy logic	MAPK signaling pathway (JNK)	Plasticity of the MAPK signaling pathway	DMSO, U0126, AZD6244, Sorafenib	A375	C	0D	no	yes
	Lee et al., 2015	Boolean	Keratinocytes, Melanocytes interplay (b-catenin)	Study melanogenesis	UVB	Keratinocytes, Melanocytes	C	0D	no	yes
	Pappalardo *et al.* , 2016	ODE	MAPK, PI3K, AKT	pERK	Dabrafenib	rat adrenal gland PC12 cells for protein level and A375 gene expression	C	0D	no	yes
mechanic	Ciarletta et al., 2011	Mixture theory, multi-phases, linear stability analysis	adherent junctions, basement membrane, nutrient, indirect necrotic core	radial growth phase	no	literature	T	2D	yes	no
	Balois et al., 2014	Two-phase mixture, physical	Interstitial fluid, oxygen, keratinocytes	Pigmented lesion shape	no	literature, tissue slide, dermoscopy	T	2D	yes	no
	Sciumè et al., 2014	PDE, TCAT, multi-phase, porous	ECM, basement membrane (BM), Interstitial fluid, oxygen, necrosis (not observed for melanoma)	Basement membrane interruption	no	compared to tissue slides, no melanoma specific parameters	CP,T,O	2D,3D	yes	no
	Taloni et al., 2014	Interwired single cells	osmotic pressure, MMP, ECM,	growth	no	IgR39, IgR37, tissue slides	CP,O,T	2D	yes	no
diagnostic	Mendes et al., 2016	Planar linear transformations	nevi shape	growth	no	Clinical, dermoscopy	O	2D	no	no
	Arasi et al., 2017	Artificial neural networks	nevi diagnostic		no	Clinical, dermoscopy	O	2D	no	no
	Satheesha et al., 2017	Multiclass classifier	nevi diagnostic		no	Clinical, dermoscopy	O	2D	no	no
blood	Dzwinel et al., 2016,Łoś et al., 2	Super modelling, coupled ODE and PDE sub-modules, single-phase, lattice	tumour growth with vasculature	growth	no	literature	O	2D, 3D	no	no
	Wang et al., 2013	agent-based	glucose, angiogenesis, Cell cycle	melanoma angiogenesis interplay	doxorubicin, Sunitinib	literature	C,CP,T	3D	no	yes
	Welter and Rieger, 2010	hybrid model	blood vessel around melanoma	drug flow simulation	no	literature	T	3D	no	no
cell culture to clinical application	Treloar *et al.* , 2013;	on lattice cellular automaton	Adhesion, proliferation, motility	Experimental parameter improvement	Mitomycin-C (stops proliferation)	MM127	C	2D	no	no
	Treloar *et al.* , 2014	on lattice cellular automaton, descrete random walk simulation	Adhesion, proliferation	Experimental parameter improvement	no	MM127	C	2D	no	no
	Vo et al., 2015	on lattice cellular automaton, approximate Bayesian Computation	Adhesion, proliferation, motility	Experimental parameter improvement	Mitomycin-C (stops proliferation)	MM127	C	2D	no	no
	Haridas et al., 2017	PDE, discrete lattice	Adhesion, proliferation, motility	cell colony expansion	Mitomycin-C (stops proliferation)	MM127/fibroblasts, SK-MEL-28	C, CP	2D	no	no
	La Porta et al., 2015	PDE	proliferation, motility	osmotic pressure impact on growth	Dextran (osmotic pressure), FBS	IgR37, IgR39	C	2D	yes	no
	Flach et. Al., 2011	ODE, compartment model	fibroblasts	cell adhesion mediated drug resistance (CAMDR)	cisplatin	WM793,1205Lu,WM1366, FF2441	CP	0D	no	no
	Picco et al., 2017	ODE, compartment model	fibroblasts	intrinsic versus environmental resistance	BRAFi (PLX4720) ; FAKi (PF562271)	5555, 4434	CP	0D	no	no
	Kim et al., 2016	ODE, compartment model	autophagy	autophagy	MK2206 AKT inhibitor	M257,WM3918	CP	0D	no	no
	Ouellet et al., 2014	compartment model	Dabrafenib Pk		Dabrafenib	clinical data	PP	0D	no	no
	Sun et al., 2016	stochastic differential equations	Connecting cancer mechanisms to population survival rates	drug-induced resistance factors,PFS, tumour cell r	Dabrafenib, Trametinib,	clinical data, 54 patients	CP,PP	0D	no	no
Immunotherapy and drug delivery	Kogan et al., 2013	ODE, phase plane analysis	CTL, DC, IL-10, IFNγ, Th1, Th2, NK	adjust immunotherapy	IL-12	based on not cited published data	CP	0D	no	no
	Eftimie and Hamam, 2017	ODE	Th1/Th2 amd Macrophages M1,M2	adjust immunotherapy		literature, B16 Melanoma	CP	0D	no	no
	den Breems and Eftimie, 2016	ODE	Th1/Th2 amd Macrophages M1,M2	adjust immunotherapy		literature, B16 Melanoma	CP	0D	no	no
	Depillis et al., 2013	DDE	CTL, DC (spleen, blood)	adjust immunotherapy	DCs infusion	literature	CP	0D	no	no
	Castillo-Montiel et al., 2015	DDE	CTL, DC TGF-β,	adjust immunotherapy	DCs infusion	mice, http://dx.doi.org/10.1155/2014/158980	CP	0D	no	no
	Santos et al., 2016	DDE,ODE	cytotoxic T cells, natural killer cells, vaccine, HLA	immunotherapy	anti-MAGE-A3 ; IL-2, IFNα	clinical data (gene signature)	CP,PP	0D	no	no
	Pappalardo et al., 2011	cellula automaton on hexagonal lattice, model size too huge for data, Gomp	CD137	CD137	anti-CD137, resting and activated OT1 T cells	B16-OVA, C57 BL/6 female mice	CP	2D	no	no
	Eikenberry et al., 2009	PDE	blood vessel, oxygen, necrosis, basement membrane, immune celles, immune attracting factor (IAF)	aggressive metastasis after excision	surgery	literature	O	3D	no	no
	Schweitzer et al., 2007	PDE	188-rhenium-labeled monoclonal antibody, melanin	antibody penetration	radioimmunotherapy	literature, C57BL6 mice	T	3D	no	no
	Liu et al., 2017	PDE	Target delivery via the skin	Target delivery via the skin	siRNA	literature	O	2D, 3D	no	no
	Ramírez-Torres et al., 2017	PDE, diffusion equation	Tumor modeling, anisotropic growth, stress, hyperelasticity			Melanoma (MU89)	T	2D	yes	no

Appreviation

C=cellular, CP= cell population, T=tissue, O=Organ , B=Body; PP=patient population

{Background}
Melanoma is a neoplasm of the skin and originates from transformed melanocytes. It causes the loss of 1.6 million disease-adjusted life-years worldwide, and the incidence rate will increase in the next decades \cite{karimkhani2017global}. ~~After~~Since the discovery of the high prevalence of mutations in \ac{braf} and \ac{nras}~~ mutations in melanoma~~ \cite{kunz2014oncogenes, omholt2003nras}, small-molecule inhibitors such as dabrafenib and vemurafenib were developed. More recently, immunotherapies, with antibodies binding immune receptors like the \ac{CTLA4} or the \ac{PDCD1}, have proven clinically effective \cite{aris2015combining}. However, many drug resistance mechanisms ~~have developed~~occurred and represent a major problem in both targeted therapy and immunotherapy \cite{amaral2017mapk, amaral2017mitogen, sharma2017primary}. As a result, life expectancy remains low. The two-year survival rate is 53.5\% for combined \ac{braf} + \ac{mek} inhibitors and 63\% for combined \ac{CTLA4} + \ac{PDCD1} immunotherapy \cite{ugurel2017survival}. Consequently, a deeper understanding of disease mechanisms is still demanded. \\
An approach to better understand causative relations, to check hypothesis consistency, but also to reveal missing qualitative information is constructing evidence-based models of these biological systems \cite{wolkenhauer2014model}. ~~Irrespective of that,~~Models depict several interconnected biological elements with a structure, which is derived from the current understanding, and parameters, which are based on data. While many life-scientists still rely on straight-forward relationships between observation and insight to extend their knowledge~~ of systems and their ability to manipulate them. However, since~~, leading scientists report that the direct link between observation and insight seems to fade \cite{weinberg2014coming~~},~~}. Thus, experimentally proven relationships are increasingly transferred into the language of mathematics to enhance our understanding of experimental findings and underlying causes. ~~Extensive simulation studies can show where an implemented hypothesis diverges from observations and can guide experiments based on \textit{in silico} if-then scenarios.~~ \\
~~Computational support tools are being developed for cancer~~ Cancer scientists can benefit from well-designed computational models, whereby systems biologists ~~focus on the~~ deliver models of cancer biochemistry, and physical oncologists ~~try to represent~~provide models of tissues. Systems biology helps understanding how biochemical pathways change during melanoma cell proliferation, invasiveness, survival, and drug resistance based on network structure and dynamic behavior \cite{kolch2015dynamic}. By contrast, physical oncology helps understanding how transport, growth, and deformations in tissues occur and is characterized by principles of geometry and mechanics \cite{mitchell2017engineering, hatzikirou2012integrative}. ~~Mechanics is especially important to understand metastatic melanoma, where cell plasticity and motility depend on mechanical characteristics of the environment \cite{ju2018role}. Consequently, the study of complex biological systems with several interconnected elements, such as melanoma, can benefit from implementing the research hypothesis in a mathematical form.~~ \\
In this review, we tried to gather all published computational models of melanoma and describe them regarding their contribution to the field. In particular, we focus on the interconnection of system elements or network characteristics while omitting classical statistics and bioinformatics of melanoma. By sorting models and methods around the topic of melanoma, we intend to support readers in finding the most appropriate mathematical models to address their melanoma~~ research questions. Even~~-specific research questions. Additionally, the review shall describe potentials for improvement, encourage readers to discover potential extensions, and create awareness of wholly missing melanoma topics to be tackled in the next decade. However, even if some models seem simplistic in biology, they often represent technically challenging stepping-stones for more biologically meaningful models in the future. ~~Readers will likely find potential extensions, improvements, or even wholly missing melanoma topics to be~~

tackled in the next decade. Consequently, reviewing the currently existing models shall help to push forward the modeling and computational characterization of melanoma. \\
Melanoma-specific databases are briefly mentioned in section 1, and networkThe review is structured as follows: Network-based approaches are explained in section 2two and contemplated by melanoma-specific repositories. The complex interaction between molecular players requires network-based approaches to suggest novel key intervention strategies, to stratify patients, and to individualize patient treatment. In section 3three, the dynamic changes in cell count of different melanoma cell types, immune cells, and fibroblasts represent a model class integratingare modeled and contemplated by stimulating or inhibiting effects between cells. Such cellular models represent another way to achieve therapy individualization and patient stratification. Section four leads to geometrical effects which will be augmented by the mechanics of melanoma in section five. Further aspects of oxygen, nutrient, and drug transport are presented in section six. The confined, spatial, and physiological tissue environment is relevant for tumor growth prognosis, drug delivery, surgery, and dermoscopic pattern recognition and will be discussed as topic 4.. All available computational melanoma models are listed in Supplemental Table 1 and organized, as shown in Figure 1.
\section{Molecular networks}
Molecular networks represent larger sets of molecules in an interconnected manner and go beyond the statistical significance of single features and the gene-set enrichment analysis paradigm \cite{subramanian2005gene}. Network science shows how biological functions emerge from the interactions between the components of living systems and how these emergent properties enable and constrain the behavior of those components \cite{wolkenhauer2014model}. In order to explore this rich information source, system biology provides frameworks tailored to each commonly known -omics data type. Melanoma-specific -omics data can be obtained from genomic \cite{akbani2015genomic, loftus2018next} and proteomic studies \cite{mittal2016proteomics} but also from the secretome \cite{liberato2018signatures} or the metabolome \cite{fischer2018metabolic, ratnikov2017metabolic}. Because multiple -omics data are rarely integrated with a systems-centered approach \cite{emmert2012statistical}, the following studies Tools and repositories are only a starting point.
\subsection{Repositories to inform network models}
Published knowledge in the form of structured and centralized searchable databases facilitates model development. Beside general sources for system biologists \cite{werner2014cancer}, melanoma-specific databases are available (Table \ref{tab: database}). The \ac{MMMP} is an open-access, participative project that structures published knowledge about molecules, genes, and pathways to enable translational perspectives \cite{mocellin2008melanoma}. The MelGene project provides an easily searchable database of genetic association studies of cutaneous melanoma, as well as a meta-analysis for many polymorphisms \cite{antonopoulou2015updated}. The MelanomaDB database lists published genomic datasets, including clinical and molecular information, and allows the creation of gene lists by merging selected studies \cite{trevarton2013melanomadb}. The \ac{MGDB} provides extensive entries about 527 melanoma-associated genes (422 protein-coding), including epigenetic and drug-related evidence. \cite{zhang2015mgdb}. Attention is required when using these databases, which accumulate data from multiple sources, sometimes in an automated manner, and are thus susceptible to perpetuate the biases and errors of the data source \cite{reinhold2013commentary}.
\section{Molecular networks and pathway modules}
Molecular networks represent larger sets of molecules in an interconnected manner and go beyond the statistical significance of single features and the gene-set enrichment analysis paradigm \cite{subramanian2005gene}. Network science shows how biological functions emerge from the interactions between the components of living systems and how these emergent properties

enable and constrain the behavior of those components \cite{wolkenhauer2014model}. In order to explore this rich information source, system biology provides frameworks tailored to each commonly known -omics data type. Melanoma-specific -omics data can be obtained from genomic \cite{akbani2015genomic, loftus2018next} and proteomic studies \cite{mittal2016proteomics} but also from the secretome \cite{liberato2018signatures} or the metabolome \cite{fischer2018metabolic, ratnikov2017metabolic}. Because multiple -omics data are rarely integrated with a systems-centered approach \cite{emmert2012statistical}, the following studies are only a starting point. We present melanoma-specific modeling studies across the omics-data types and begin with an approach based on genomic data.

\subsection{Models for melanoma genomics}

The melanoma-specific repositories contain mainly genetic data with not yet fully identified patterns. The mutation pattern within the genome of metastatic melanoma can be used to find mutually exclusive gene modules \cite{ciriello2012mutual}. If two proteins are related in an interaction network and their genes are mutated in a way that one gets amplified while the other gets deleted or only one gets modified without the other, one could presume that this happens to intensify cancer pathways at the protein level under given pathophysiological pressure. Consequently, one can conclude that a protein inhibits or activates the other in a known interaction network. The pathophysiologic pressure on cancer protein pathways selects mutation patterns with survival benefits. One analysis of \ac{TCGA} melanoma samples integrated somatic mutations with copy number alterations and found concomitant deregulation of the G-protein and MAPK signaling pathways \cite{guan2015cancer}. Similarly, integrated genomic and epigenomic analyses have been used to classify melanoma brain metastases in different mutually exclusive molecular subtypes \cite{marzese2014dna}.

\subsection{Models for melanoma transcriptomics}

The melanoma transcriptome is more context-specific than the genome and easier to measure than the proteome. The pattern changes can be used to stratify patients or to identify drug targets. Beyond this, they can give an impression of the re-wiring of pathways. Barter \textit{et al.} applied three different strategies (single genes, gene sets, and network analysis) to 47 melanoma microarray datasets. They concluded that network methods do not perform better overall, that these different approaches tend not to classify patients consistently, and that the optimal method might have to be identified patient-specifically \cite{barter2014network}. Wang \textit{et al.} performed 45 siRNA screens of the A375 cell line, whole-genome sequencing, and Bayesian gene network interference to enable directional and synergistic conclusions. Similar to Barter's findings, the network hubs alone were not sufficient to better stratify patients. However, if the network hubs are contextualized with cell-cycle and \ac{dna}-repair function, the prediction of an individual prognosis is possible \cite{wang2012cell}. The concept of pathway re-wiring is based on the following reasoning. Some mutations can cause modified protein structures, which in turn can alter the links between the proteins without a change in protein concentration levels. Two proteins are interacting if the proteintranscript level change of one protein correlates or anti-correlates with the transcript level change of another protein. When this co-expression gets lost, the connection gets lost, and the connectivity reduces. When two unrelated proteins show a new co-expression in the next progression stage, the connectivity increases, and a pathway re-wiring can be assumed. This network analysis can be performed independent of significantly changed differential expression and fold changes. Kaushik \textit{et al.} followed this strategy and meta-analyzed 632 melanoma microarray samples with melanoma progression stages: normal skin, non-metastatic (radial and vertical growth phase), metastatic, and lymph node metastases \cite{kaushik2015gene}. They diversified the clinical relevant groups by pooling the data of tissue samples with untreated and cisplatin-treated melanoma cell lines and melanocytes. The extracted re-wired pathway hubs were subsequently checked for drugability, which is important as many

promising targets cannot be influenced pharmacologically \cite{dang2017drugging}.
\subsection{Models for melanoma proteomics}
The proteome directly mirrors cellular function. Genomic and transcriptomic data do not show the post-transcriptional, translational, and further epigenetic changes and are thus limited in their representation of final physical processes. Proteomic data is, \textit{e.g.}, very beneficial for modeling the signal transduction such as MAPK or \ac{PI3K} pathway \cite{saez2015modeling}. In the context of melanoma, most studies aim either at understanding resistance mechanisms or at the responses to particular compounds. \\
For example, it was possible to predict with high accuracy the apoptosis susceptibility of 11 melanoma cell lines to TRAIL and \ac{DTIC} using 17 protein measurements. This was achieved by grouping measurements in pathway-inspired functional groups and using these in multivariate statistical analysis \cite{passante2013systems}. \\ Resistance in melanoma cell lines was studied with data-driven modeling and multivariate statistics. 21 phosphoproteins were measured over time in a panel of 10 cell lines subjected to different doses of five different RAF/\ac{mek} inhibitors \cite{fallahi2015systematic}. This led to the identification of an early down-regulation of the \ac{jnk}\footnote{Also known as c-Jun N-terminal kinases JNK}/\ac{jun} pathway upon RAF/\ac{mek} inhibition, but an up-regulation in six cell lines at later time points. This study showed that a fraction of treated cells become quiescent and apoptosis-resistant. The same group further validated these results and suggested targeting \ac{jnk}, \ac{fak}, or \ac{src} to inhibit this particular drug-resistant phenotype \cite{fallahi2017adaptive}. \\ Bernardo-Faura \textit{et al.} used Fuzzy Logic to investigate the temporal network re-wiring in A375 cells in response to different kinase inhibitors. The authors used a prior-knowledge network to simulate the behavior of the cells over time, and detected discrepancies at specific time-points between the model predictions and the measurements. This work, as well, underlines the importance of the \ac{jnk} pathway in early drug-induced changes in signaling pathways \cite{bernardo2014data}. \\ Del Mistro \textit{et al.} studied the signaling network changes in phosphor-proteomic data due to underlying resistance of mutated \ac{braf} melanoma cell lines to sublethal \ac{trail} receptor-targeted agonist IZI1551. Systemic network analysis with Dynamic Bayesian modeling identified \ac{xiap} and \ac{ikba} as potential drug targets. Consequently, targeting these nodes in the subsequent experimental validation led to a re-sensitization of the cell lines \cite{del2018systemic}. \\ Another comprehensive study ~~studied~~is about the impact of \ac{myc} on the proteome and drug resistance, which lead to the identification of a co-targeting strategy \cite{korkut2015perturbation}. ~~Especially this paper raised some interest. However, the interactions with the environment, the restriction to only one cell line, and the limited appropriateness of the used drugs for clinical purposes show that it is still a long way to support physicians in decision-making \cite{levesque2015perturbing}.~~
\subsection{Models for melanoma metabolomics}
The metabolic state is the consequence of proteomic function and environmental conditions such as nutrient and oxygen shortages. Metabolite concentrations can be obtained with robust measurements, and well-established methods are available (Antoniewicz, 2015). Notably, Scott \textit{et al.} used metabolic flux analysis to characterize the response of seven melanoma cell lines to hypoxia \cite{scott2011comparative}. They showed the crucial roles of both Warburg and Pasteur effects in melanoma and paved the way for the therapeutic targeting of metabolism. While the Pasteur effect describes reduced glycolysis with increased oxygen, the Warburg effect refers to cancer cells performing glycolysis despite the presence of oxygen \cite{koppenol2011otto}. Future studies might further combine metabolic modeling with other omics-data.
\subsection{Mechanistic network models for melanoma}

Completely validated mechanistic network models of melanoma seem not been published yet, but a valid Boolean model of melanogenesis covers both keratinocyte and melanocyte signaling. Lee \textit{et al.}, thereby, imposed increasing \ac{uvb} light intensity and modeled the cellular response to it. The simulated profiles of the protein levels were individually compared to literature to check qualitative plausibility. Lee \textit{et al.} demonstrated the central role of \ac{Bcat} in the regulation of both melanogenesis and apoptosis. This prediction was then validated using \ac{uvb}-exposed reconstituted human skin equivalents \cite{lee2015systems}. Moreover, a system of \ac{ode} was used to model the MAPK, \ac{PI3K}/\ac{act}, and other pathways with 48 species and 48 biochemical reactions \cite{pappalardo2016computational}. The model was an extension of the model of PC-12 (rat adrenal gland) cells ~~from Brown \textit{et al.}~~and \cite{brown2004statistical~~}. The model~~} shows that increasing dabrafenib concentrations cause declining pERK concentration but in unphysiological ranges. Future \ac{ode}-based modeling of melanoma signaling would ideally improve the balance between model size and melanoma-specific data ~~(Akaike information criterion)~~ to enable robust predictions. Sensitivity analyses and a model selection procedure might help to suggest key mechanisms and intervention strategies. \\
\ \\
As described in this section, the network information can be used to stratify patients, to find druggable targets, and to understand the impact of therapy on the biochemical pathways. The next section describes models to inter-connect cells instead of molecules. Cell population models are used to find coherencies between cell culture and clinical patient populations or to understand the immune system at the whole-body level.
\section{Cell population models: bridging cell culture to clinics}
Melanoma cells are not isolated entities and interact with keratinocytes, fibroblasts, and ~~the various cells of the~~ immune ~~system~~cells. Moreover, melanoma cells might be divided into subtypes or phenotypes. Population models often describe the interaction between them, \textit{e.g.}, how the abundance of one cell population influences the abundance of another cell population. A subset of these models integrate cell culture data; another subset of these models are experimentally adjusted with human or murine \textit{in vivo} data.
\subsection{Melanoma models can mimic the interplay of cell types}
Flach \textit{et al.} studied the interplay of melanoma cells, stromal fibroblasts, and stromal fibronectin. ~~It is rather a simple \ac{ode} model of spheroid growth.~~ In their interpretation, free melanoma cells at the stromal interface activate fibroblasts to get mechanical support. The mechanically supported cells proliferate ~~then~~ until they become blocked due to space limitations, albeit the space limitation is simplified to state values in this ODE network model \cite{flach2011fibroblasts}. Accordingly, several studies point to the crucial role of \ac{ecm} remodeling, fibronectin, and \ac{fak} signaling in driving resistance to \ac{braf} inhibitors \cite{hirata2015intravital, fedorenko2016fibronectin}. This conceptual model of Flach \textit{et al.} has been refined, validated, and extended to \ac{braf}i and \ac{fak}i therapy \cite{picco2017integrating}. ~~These~~The results allowed a deeper understanding of the role of stroma during acquired resistance and its potential role during targeted therapy in drug-resistant patients \cite{flach2011fibroblasts, picco2017integrating}. The same group worked on a dynamic autophagy model with \ac{act}i therapy for melanoma \cite{kim2016phase}. After cell culture and clinical patient data had been integrated into the autophagy model~~,~~ and key stratification parameters were identified. Stratification parameters could either accompany clinical trials or support treatment choice. Another melanoma cell population model is provided by Sun \textit{et al.} with an excellent description of the parameter origin. The considered cell types are ~~primary~~ \ac{braf}i sensitive, ~~and~~ \ac{braf}i resistant, and ~~which~~ may or may not enter the metastatic cell state after the initiation of drug treatment. Cells grow until a maximum cell burden. The set of stochastic differential equations with 19 parameters is experimentally adjusted via

circulating tumor cell \ac{dna} and melanoma cell line data. Progression-free survival is set equal with the melanoma cell concentration for simplicity \cite{sun2016mathematical}, whereby more data might allow a more clinical relevant linkage between these two. Future models with integrated pharmacokinetic elements might consider clinically relevant pharmacokinetic models \cite{ouellet2014population}.
\subsection{Cell interplay is studied for melanoma immunology}
Cell population models for the interplay of melanoma cells with immune cells are helpful as melanomas are highly immunogenic tumors \cite{herzberg2016metastatic}. This high immunogenicity is the reason for the success of therapies based on immune activation in this tumor type. Indeed, melanoma was the first cancer type for which an immune checkpoint inhibitor and an oncolytic virus were approved \cite{hodi2010improved, andtbacka2015talimogene}. As such, several computational models have been specially developed to study the interplay between ~~the~~ immune and melanoma cells. For example, several \ac{ode} systems were devised to model ~~the tumor relationship~~melanoma with Th1 and Th2 helper lymphocytes \cite{kogan2013mathematical}, with \ac{NK} cells in the context of \ac{IL21} therapy \cite{cappuccio2006cancer}, with M1 and M2 macrophages \cite{den2016re}, or both macrophages and helper lymphocytes \cite{eftimie2017modelling}. Also, vaccine strategies based on dendritic cell therapy for melanoma were modeled with a multi-compartment \ac{ode} system to define adequate doses and schedules \cite{depillis2013model}. However, one drawback of these models is that the patients' intrinsic variables, key determinants in immune-related therapies, are not taken into account \cite{pizzurro2015dendritic}. One study took into account the genetic signatures being associated with resistance to immunotherapies~~, which helped the parameterization of an~~. The parameterized \ac{ode} model ~~and~~ suggested co-adjuvants for successful ~~anticancer~~ vaccine therapies \cite{santos2016model}. In another study, Pappalardo \textit{et al.} implemented an on-grid cellular automaton model of melanoma, in which melanoma cells interact with macrophages, T cells, and dendritic cells in different cellular states. Pappalardo \textit{et al.} highlighted the role of \ac{CD137} for successful therapy and adjusted their model with experimental mice data of activated or resting OT1 T-cells and anti-\ac{CD137} antibodies in B16 melanoma \cite{pappalardo2011simb16}. Given the size of the model, additionally experimental data would further improve model parameterization and robustness \cite{altrock2015mathematics}. \\
\ \\
In summary, cell-population models can combine clinical and cell culture data and might support the determination of an individualized drug regimen based on cellular dynamics. While these models are suitable for freely acting cells, tumors are frequently restricted by the \ac{ecm} and anatomical space limitations. These effects were simplified by three models mentioned above \cite{flach2011fibroblasts, pappalardo2011simb16, picco2017integrating}. While ~~the first~~one refers to \ac{3d} spheroid growth in collagen gel, ~~the latter~~ two refer to tumor size in mice. Tumor growth is more complex and ~~will be~~requires spatial, mechanical, and physiological characteristics being addressed in the ~~next section.~~following three sections.
\section{Spatial models for melanoma}
~~Revealing the molecular networks within cells is a crucial step to develop appropriate drug combination therapies for melanoma. Cell population models are an additional way to stratify patients and to individualize the therapeutic regimen. However, the spatial tumor expansion in tissue has played a subsidiary role heretofore, and physical barriers modify the expansion rate. Consequently, the biological interactions with spatially distributed environmental factors are addressed in the following.~~
The spatial tumor expansion in tissue has played a subsidiary role heretofore. In the following, spatially distributed factors of lesion and environment are addressed. For instance, spatial patterns in dermoscopic pictures can be used to classify a particular lesion to obtain hints for prospective growth and the necessity of surgical intervention.

Subsequently, combining cell-population models with geometry provide insights into the success of surgical therapy. When focussing on the cellular level, the collocation of cells can partly point to factors with the most control cell mass expansion. However, a more in-depth look at histological features of skin and other host tissues reveal that solely geometrical solutions may not be sufficient as mechanical cues significantly impact deformation and development.
\subsection{Pattern recognition of melanoma}
The pattern of naevi and melanoma \textit{in situ} are the physical consequence of biochemical processes in the epidermis and are usually assessed and classified in dermatology to initiate early therapy. The related patterns can be modeled in two dimensions using a mixture theory model \cite{balois2014morphology}. The study shows how different patterns of malignant cells can form within a healthy cell environment. \ac{2d} patterns of naevi and melanoma can also be subjected to planar linear transformations using two subsequent dermoscopy pictures. Those pictures allow the classification of melanoma growth rates and naevi symmetry \cite{mendes2016geometric}. The ABCD criteria for melanoma have been mathematically considered too \cite{lee2017mathematical}. Automated optical classification of naevi and melanomas is a fast-growing field and employs machine learning methods for image recognition. The sensitivity and specificity of these models matched the decision quality of dermatologists \cite{esteva2017dermatologist, haenssle2018man, marchetti2018results}. Specific features in \ac{2d} dermoscopy pictures can also be used to determine the Breslow depth with specificity and sensitivity of almost 100\%, which has direct prognostic value \cite{satheesha2017melanoma}. furthermore, the depth of invasion is an important prognostic marker for patient survival, and the Breslow index can be determined manually or automatically from histopathological images \cite{haenssle2018man, xu2017computerized}.
\subsection{Models of surgical treatment}
Surgical treatment is the consequence of early identified melanomas. Wide excision of primary melanoma can have counter-intuitive ramifications according to the reaction-diffusion model of Eikenberry \textit{et al.}. The surgical resection of primary melanomas might include tumor-associated immune cells, which lead to an accelerated outgrowth of local metastasis due to reduced immune suppression \cite{eikenberry2009tumor}. Computational models are also used to assist image-guided and computer-assisted surgery, mainly for the brain \cite{payan2012soft}. The brain, besides lung and lymph nodes, is a preferred host tissue for metastatic melanoma \cite{shain2016melanocytes}.
\subsection{Dissecting parameters in spatial models is a challenge}
Fully experimentally validated models of melanoma expansion are still limited to simple Petri dish experiments. In a series of reports, Treloar \textit{et al.} use a lattice cellular automaton model and an experimental approach to identify different parameters of MM127 colony growth where cell motility, cell-to-cell adhesion, and cell proliferation influence the same: the expansion of the cell colony \cite{treloar2014assessing, treloar2013multiple}. These parameters were also estimated using a Bayesian framework coupled with a stochastic model of \ac{2d} melanoma growth \cite{vo2015melanoma}. Using melanoma and fibroblast monocultures as well as different co-culture systems, Haridas \textit{et al.} have parameterized a \ac{pde} model of the interactions of cancer cells and fibroblasts \cite{haridas2017quantifying}. Continuous modeling of melanoma cells under different osmotic pressures was performed with a \ac{2d} lattice model to simulate scratch assays \cite{la2015osmotic}. The aim was to distinct migration/invasion between primary and metastatic cells. New vertex modeling strategies \cite{barton2017active} and scratch assay analysis tools \cite{stichel2017individual} might further improve this approach.
\subsection{Spatial organization of skin and confined spaces}
The previously described spatial parameter determination strategy for cell lines is especially helpful for the epidermal skin layer. However, the skin is more complex and also contains irregular fibrous tissue beneath the

epidermal layer separated by a collagenous basement membrane \cite{breitkreutz2013skin}. At the dermal-epidermal junction, keratinocytes are generated and migrate through the epidermis up to the skin surface, where they keratinize to the stratified protective barrier called stratum corneum. The epidermal layer is also the most common location for melanoma initiation. Residing melanocytes can become benign neoplasms and appear as innate or acquired naevi \cite{shain2016melanocytes}. Further changes and appearing atypical cells constitute the first malignant stage: the radial growth phase. From the clinical perspective and the perspective of modeling, the basement membrane is crucial. Invasion through the basement membrane indicates the vertical growth phase, which may require adjuvant therapy besides surgical treatment. Pharmacological therapy is indicated for metastatic growth in secondary tissues. In contrast to the epidermis, the dermis layer is streaked with collagen and elastin fibers synthesized by fibroblasts \cite{evans2013epithelial}, and these \ac{ecm} fibers restrict tumor expansion \cite{nia2016solid}. Using colony growth in \ac{2d} cell culture experiments does not lead to quantitative parameters for spatial models representing stromal processes. For example, migration velocity depends on the \ac{ecm} fiber geometry \cite{tozluouglu2013matrix}, the migration process is fundamentally different in confined structures \cite{paul2017cancer}, and depend on the \ac{PXN} and \ac{hic5} balance related to \ac{fak} \cite{deakin2011distinct}. Moreover, \ac{braf} inhibition promotes \ac{mmp} activity and cell migration in three dimensions \cite{leight2015multifunctional}. A consequent experimental parameterization of realistic melanoma growth models is difficult to find and is aggravated by the diversity of parameter origin and their mutual dependency, as shown by Treloar \textit{et al.} \cite{treloar2014assessing, treloar2013multiple}. The modeling of the tumor microenvironment has to consider additional factors like extracellular matrix stiffness and topography, oxygen and nutrients gradients, and interstitial fluid pressure \cite{holle2016vitro}. \\
\ \\
Not only the parameterization is challenging, but also the computational framework is tedious if the correct physiological modeling of the host tissues is required. Physical models are used to understand the progression of melanoma, the contortion of the tumor tissue, and the drug penetration of tumor tissue. Physical models are also used in image-guided surgery.

\section{Mechanical models of melanoma}

Mechanical cues in the environment influence directly important biochemical cancer pathways and have a complex impact on tumour progression \cite{hutchenreuther2018target, ju2018role}. Consequently mechanical models become more attention and three methods will be presented in the following such as mixture theory, the \ac{tcat}, and the discrete ansatz with cross-linked elastic cells. These three methodologies can mimic the growth in tissues, while a tissue without any malignant contortions is already a complex modelling task \cite{lanir2017multi}. As the integration and measurement of mechanical cues is not yet widely used, a summary of experimental methods is given.

\subsection{Impact of mechanoregulation}

In three dimensions, additional factors impair drug sensitivity \cite{hirata2015intravital, shao2010akt3} and increase or decrease the tumor growth rate \cite{ambrosi2017solid}. The stromal environment causes non-genetic phenotype switches between proliferative and mesenchymal stages \cite{levesque2017metastatic, hoek2008vivo}, and environmental melanoma-associated fibroblasts are suspected of playing an essential role in melanoma progression \cite{izar2016bidirectional}. Fibroblast activity is closely linked to ECM and thus biomechanics, which is now recognized as a central pillar of tumor progression and metastasis \cite{hutchenreuther2018target, ju2018role}. Mechanical melanoma models consider the growth-induced deformation of the \ac{ecm} rich environment. The more the proliferating mass expands, the more counterforce is generated by the connected \ac{ecm} fibers. The elastic energy is conserved and

geometry dependent \cite{nia2016solid}. The mechanical deformation of tissues and mechanical stress influence intracellular signaling by mechano-sensors like \ac{fak} \cite{paszek2005tensional} or \ac{YAP}/\ac{taz} \cite{halder2012transduction}, which are discussed as drug resistance mechanism for \ac{braf}-mutant melanoma cells \cite{fallahi2017adaptive, hirata2015intravital, kim2016actin} or progression marker for cutaneous and \ac{GNAQ} mutant uveal melanoma \cite{feng2014hippo, sanchez2014hippo, nallet2014pro}. Proximity to mechano-regulating fibroblasts can induce pathway changes to \ac{PI3K}/\ac{mtor} and switch the phenotype of melanoma cells to the mesenchymal state \cite{seip2016fibroblast}. Consequently, melanoma cells reduce the inherent stiffness to facilitate invasion \cite{weder2014increased, wei2016forcing}. However, our knowledge of mechanosensitive pathways is far from complete \cite{ringer2017sensing, northey2017tissue, charras2018tensile, wei2016forcing}, and mechanical phenomenons require computational models to comprehend. \\
Additionally, the skin, being the primary site for cutaneous melanoma, is a mechano-sensitive organ. Skin can grow when it is stretched, and rete ridges, projections of the epidermis into the dermis, were recently suspected to form according to mechanical characteristics \cite{evans2013epithelial}. The skin has inspired many computational models describing dermal transport processes as well as providing a mechanical understanding of the skin's optical, functional, and structural characteristics \cite{querleux2014computational}.
\subsection~~{Mechanical models of melanoma}~~
~~Mechanical models of melanoma are build up with the mixture theory, the \ac{tcat}, or the discrete ansatz with cross-linked elastic cells.~~
~~\subsubsection~~{Mixture theory}
Two mixture theory models exist. One describes the skin surface, and one mimics the vertical section \cite{balois2014morphology,ciarletta2010radial}. Balois \textit{et al.} consider interstitial fluid pressure, a mechanically optimal cell density, and friction between the melanocytic lesion and the surrounding. Ciarletta \textit{et al.} represent melanoma in the radial growth phase in the ECM free epidermis as a viscous fluid sliding on a basement membrane with friction dependent growth velocity. In a second step, this friction is neglected and instead considered between the basement membrane and an additional keratinocyte representing fluid. Melanoma cell and keratinocyte fluid are adjacent to each other, and the tumor front between them is a moving interface/ free boundary problem subjected to stability analysis.
\\~~subsubsection~~subsection{TCAT theory}
TCAT models \cite{sciume2014tumor~~}, however,~~} represent a multi-phase approach, which is different from mixture theory and circumvents the free boundary problem. \ac{tcat} models do not have a defined tumor boundary at the macro-scale, but the \ac{ecm} spans the whole tissue, with a higher concentration at the basement membrane. The interstitial fluid, the healthy, and the malignant cells squeeze via local rules through the solid but deformable porous \ac{ecm} network. Averaging of local properties causes a macroscale behavior that resembles the distortion of the tissue and the invasion of the basement membrane. By adjusting the cancer cell plasticity but not \ac{ecm} integrity, the model changes from solid to invasive growth \cite{albrecht2016thermodynamically}.
~~\subsubsection~~\subsection{Disordered lattice model}
The discrete model \cite{taloni2014mechanical} describes individual cells on a \ac{2d} disordered lattice. Cells are represented as spheres, which are connected via breakable springs. The springs mimic ECM and cell-cell contacts. Melanoma induced \ac{mmp} activity is modeled by a higher probability of spring breaking near melanoma cells. Despite the simple mechanical and geometrical laws, the simulation results give a realistic impression. Because discrete models are more computationally demanding than continuous models, they allow only limited upscaling. However, the benefit of this single-cell modeling approach is the potential discrimination between compressive and tensile stress, which can differ strongly across the \ac{ecm} biopolymer types \cite{pritchard2014mechanics} and tumor

locations \cite{nia2016solid}. The model by Taloni \textit{et al.} was validated with \ac{2d} experiments. The experiments were performed under osmotic pressure without fibronectin, which is an important linker between mechanics and intracellular signaling.

\subsubsection{Difficulties in determiningsubsection{Experimental methods for mechanical parametersmelanoma models}

Although modeling promises to become more and more prominent in melanoma research, and continuous improvements in computational power make more complex and realistic models accessible, experimentally validated parameterization remains a crucial bottleneck. To produce high-quality mathematical models, quantitative data under standardized operating procedures are required \cite{silk2014model, stanford2015evolution}. \\
Tumor spheres and spheroids in general \cite{weiswald2015spherical}, and organotypic \textit{in vitro} models for melanoma \cite{kulms2018vitro} in particular offer more realistic experimental conditions. Fully functional organotypic skin constructs \cite{vorsmann2013development} can mimic all melanoma progression stages. \ac{3d} constructs are not only a carrier of cells; they modify the experimental outcome. Thus, quantification of the hydrogel system parameter, such as the shear or Young's modulus becomes standard. The shear modulus G of the gel system, or the roughly three times higher Young's modulus E, is stated with the unit kPa (E=2G(1+ν); ν: Poisson's ratio) \cite{hirata2015intravital, northey2017tissue, paszek2005tensional}. Knowing the impact of mechanical cues in the modeling process prevents common data integration problems. For example, the frequently used matrigel for invasion assays has an elastic modulus of 0.45 kPa and is consequently a weaker obstacle than the basement membrane reaching 250-500 kPa \cite{halfter2015new, soofi2009elastic}. Additionally, the impact of stress relaxation should be not underestimated as it has a decisive impact on further development \cite{chaudhuri2016hydrogels}. A range of hydrogel systems is available \cite{caliari2016practical} and can also be used for automated drug testing \cite{rimann2014automation} albeit questions of standardisation of \ac{3d} cell culture models remain \cite{verjans2018three}. \\
Mechanical parameters are difficult to measure, and tissue is already complex without any malignant contortions \cite{lanir2017multi}. Mechanical tissue parameters and span up to 5 log steps depending on tissue moisture and experimental setting \cite{derler2012tribology}. Some recent developments in experimental strategies are hardly known by experimental and computational scientists but can inform computational models.

Experimental mechano-sensors enable the measurement of sub-molecular force transmissions \cite{grashoff2010measuring}, and fluorescent oil microdroplets allow the measurement of anisotropic stress fields in \ac{3d} tissues \cite{campas2014quantifying}. Tunable alginate microcapsules can be used to determine the mechanical growth-pressure of spheroids \cite{alessandri2013cellular}, and high throughput mechanical testing of cells is possible with optical deformation of cells \cite{otto2015real}. If the direct measurement of stiffness is not possible, the \ac{LMNA} to \ac{LMNB} ratio serves as an appropriate biomarker \cite{swift2013nuclear}. \\
The clinical imaging technology elastography gives direct access to the tissue stiffness fields and thus tumor locations \textit{in vivo} \cite{kim2016application}. Elastography can also be used for the \textit{in vivo} staging of melanoma \cite{jid2015doppler}. The integration of elastography and melanoma mechano-signaling could visualize thehighlight stiff areas where mechano-sensors influence melanoma pathways. This could facilitate the translation of these research models into clinicalclinically relevant predictive models. \\
The complexity of tumor cell environment interactions requires a step-wise understanding with a multitude of experimental techniques \cite{peela2017advanced} and related computational efforts (Figure 1). Computational scientists must incorporate the experimental context to develop meaningful computational melanoma progression models.

\section{Transport of oxygen and drugs}

Several models describe how oxygen and drugs move from the source to a melanocytic lesion as the presence of oxygen and nutrients control the viability of cancerous and healthy tissues. Thereby, multiple oxygen sources as well as vascular and pericellular transport routes matter as described in the following.
\subsection{Oxygenation of melanoma in skin and brain}
Impaired oxygen and nutrient delivery cause necrotic cores, which is a widespread assumption. A necrotic core can be modelled explicitly \cite{sciume2014tumor} or indirectly via nutrient concentration reduction \cite{ciarletta2010radial}. However, Thibaut Balois \& Martine Ben Amar question the existence of necrotic cores in epidermal melanoma and take the atmospheric oxygen source into account \cite{stucker2002cutaneous, balois2014morphology}. If oxygen came only from the ~~dermis~~dermal vasculature, the oxygen partial pressure would drop to around eight mmHg at the skin surface~~\footnote{Skin surface was sealed with paraffin oil}~~ \cite{wang2003oxygen}. Mild hypoxic conditions are present around the basement membrane, promoting melanocyte proliferation \cite{horikoshi1990effects} as well as melanoma progression \cite{hanna2013hif1alpha}. Interestingly, the brain, a common location for metastasized melanoma, has also a low tissue oxygen concentrations reaching 35 mmHg \cite{carreau2011partial}.
\subsection{Experimental aspects of oxygen}
Most established melanoma cell lines are cultured under atmospheric oxygen and are therefore evolutionarily adjusted to these artificial conditions. Molecular oxygen sensors for \ac{3d} settings \cite{dmitriev2015versatile} are as possible as advanced hypoxia sensors \cite{erapaneedi2016novel} to improve the validation of computational models. Oxygen consumption rates of cells can be obtained with the Seahorse technology \cite{brand2011assessing} and were determined for melanocytes and melanoma cell lines \cite{hall2013dysfunctional}.
\subsection{Models of melanoma-associated vascularization}
The tumor-associated vascularization is ~~a consequence of~~ influenced by oxygen limitations and ~~influenced by~~ mechanical cues \cite{chwalek2014glycosaminoglycan, balcioglu2016tumor}. Mathematical blood vessel models define an independent computational research field \cite{scianna2013review}. Notably, Welter and Rieger combined the discrete modeling of vasculature remodeling with the continuous gradients of melanoma cells, oxygen, nutrients, and drugs \cite{welter2010physical}. They used melanoma-specific data for the vasculature \cite{dome2002vascularization}. This excellent model is useful to simulate blood flow and to study the impact of space limitations on simple drug diffusion and nutrient supply. Wang \textit{et al.} created an agent-based model with both melanoma and endothelial cells with a focus on angiogenesis. They tested the combined effect of doxorubicin chemotherapy and \ac{VEGFR}\footnote{Also known as vascular endothelial growth factor receptor 2 (VEGFR)} inhibition with sunitinib \cite{wang2013multi}. It might be interesting to see a follow-up model with improved use of biological data for parameter, synergy, and validation. Dzwinel \textit{et al.} coupled several continuous sub-models of melanoma growth to increase modeling quality and efficiency. They used a single phase continuum for growth accompanied by angiogenesis, vascular remodeling, and tumor \ac{ecm} interactions. The model was embedded in a realistic virtual skin structure, and the melanoma progression resembled nodular, lentigo maligna, and acral lentiginous melanoma \cite{dzwinel2016supermodeling}. The same group extended the model by a discrete vascularization dynamic, which was coupled intermittently \cite{los2017application}. The used approach, called "super-modeling" by the authors, is a theory on model synchronization \cite{duane2017introduction}. However, the connection coefficients seem untrained in comparison to non-biological application areas, and the coupling remains weak \cite{duane2017introduction}. This modeling group is very active in melanoma, refines the model continuously, and also uses particle automata models to produce visually realistic models \cite{klusek2019efficient, panuszewska2018pam}. \\

In general, while these models provide valuable insight into the vasculature, much work is needed to ensure adequate melanoma-specific parametrizations and validations. Einar Rofstads' group provides excellent data sets on melanoma-associated vascularization and might be considered for further modeling projects \cite{gaustad2012sunitinib}.
\subsection{Drug delivery models}
Blood vessels are an essential route for drugs to the location of action, and pharmacokinetics is studied to determine the drug concentration in local blood plasma. However, the transport from the blood vessels or skin surface to the melanoma cells depends on the diffusion coefficient of the microanatomical structure. Drug delivery models are available for both the penetration of spherical tumors with melanin-binding antibodies for radioimmunotherapy \cite{schweitzer2007computational} and SPACE-EGF mediated transdermal delivery of \ac{myc} siRNA \cite{liu2017theoretical}. However, the impact of biomechanics and tumor physiology on drug delivery is not considered by those models but discussed for MU89 Melanoma in mice \cite{stylianopoulos2012causes, ramirez2017influence}. A proliferating mass makes fibrous tissue crowded and compressed. This might lead to a reduced interstitial fluid volume fraction and thus impaired drug transport. Such a phenomenon might be best modeled with the multi-phase flow in porous media \cite{sciume2014tumor}.
\subsection{Pattern recognition of melanoma}
The pattern of naevi and melanoma \textit{in situ} are the physical consequence of the above-described processes in the epidermis and are usually assessed and classified in dermatology. The regular differentiation between harmless naevi and potential melanomas by a dermatologist enables early therapy. The related patterns can be modeled in two dimensions using a mixture theory model \cite{balois2014morphology}. The study shows how different patterns of malignant cells can form within a healthy cell environment. \ac{2d} patterns of naevi and melanoma can also be subjected to planar linear transformations using two subsequent dermoscopy pictures. Those pictures allow the classification of melanoma growth rates and naevi symmetry \cite{mendes2016geometric}. The ABCD criteria for melanoma have been mathematically considered too \cite{lee2017mathematical}. Automated optical classification of naevi and melanomas is a fast growing field and employs machine learning methods for image recognition. The sensitivity and specificity of these models matched the decision quality of dermatologists \cite{esteva2017dermatologist, haenssle2018man, marchetti2018results}. Specific features in \ac{2d} dermoscopy pictures can also be used to determine the Breslow depth with specificity and sensitivity of almost 100\%, which has direct prognostic value \cite{satheesha2017melanoma}. The depth of invasion is an important prognostic marker for patient survival, and the Breslow index can be determined manually or automatically from histopathological images \cite{haenssle2018man, xu2017computerized}.
\subsection{Models of surgical treatment}
Surgical treatment is the consequence of early identified melanomas. Wide excision of primary melanoma can have counter-intuitive ramifications according to the reaction-diffusion model of Eikenberry \textit{et al.}. The surgical resection of primary melanomas might include tumor-associated immune cells, which lead to an accelerated outgrowth of local metastasis due to reduced immune suppression \cite{eikenberry2009tumor}. Computational models are also used to assist image-guided and computer-assisted surgery, mainly for the brain \cite{payan2012soft}. The brain, besides lung and lymph nodes, is a preferred host tissue for metastatic melanoma \cite{shain2016melanocytes}.
\subsection{Spatial melanoma models need specific experiments}
Although modeling promises to become more and more prominent in melanoma research, and continuous improvements in computational power make more complex and realistic models accessible, experimentally validated parameterization remains a crucial bottleneck. To produce high-quality mathematical models, quantitative data under standardized operating procedures are required \cite{silk2014model, stanford2015evolution}. Tumor spheres and spheroids in general \cite{weiswald2015spherical}, and

organotypic \textit{in vitro} models for melanoma \cite{kulms2018vitro} in particular offer more realistic experimental conditions. Fully functional organotypic skin constructs \cite{vorsmann2013development} can mimic all melanoma progression stages. \ac{3d} constructs are not just a carrier of cells; they modify the experimental outcome. Thus, quantification of the hydrogel system parameter, such as the shear or Young's modulus becomes standard. The shear modulus G of the gel system, or the roughly three times higher Young's modulus E, is stated with the unit kPa (E=2G(1+ν); ν: Poisson's ratio) \cite{hirata2015intravital, northey2017tissue, paszek2005tensional}. For example, the frequently used matrigel for invasion assays has an elastic modulus of 0.45 kPa and is consequently a weaker obstacle than the basement membrane reaching 250-500 kPa \cite{halfter2015new, soofi2009elastic}. Additionally, the impact of stress relaxation should be not underestimated \cite{chaudhuri2016hydrogels}. A range of hydrogel systems is available \cite{caliari2016practical} and can also be used for automated drug testing \cite{rimann2014automation} albeit questions of standardisation of \ac{3d} cell culture models remain \cite{verjans2018three}. The complexity of tumor cell environment interactions requires a step-wise understanding with a multitude of experimental techniques \cite{pecla2017advanced} and related computational efforts. Computational scientists must incorporate the experimental context to develop meaningful computational melanoma progression models. \\
\ \\
This section showed that spatially resolved models can give us further insight into surgical interventions, drug delivery, and interactions between melanoma cells and stroma. Tissues can promote and constrain tumor progression via the extracellular matrix or the blood vessel system. {The modeling of the tumor microenvironment has to consider additional factors like extracellular matrix stiffness and topography, oxygen and nutrients gradients, and interstitial fluid pressure \cite{holle2016vitro}. How difficult the parameter determination can be, has been shown with the colony growth \cite{treloar2013multiple} and with the section on mechanical cues. Parameters are crucial to have a falsifiable test hypothesis for model selection. Only in this way, a deeper understanding of the complex relationships can be achieved. Unfortunately, many publications work with uncurated parameter lists, and interested readers are recommended to trace back parameter values to the primary source to judge the validity. At the moment, the field of physical oncology is still in transition. Modelers focus on computational frameworks creating patterns, which are qualitatively comparable with \textit{in vivo} observations. A more sophisticated way might be the stepwise model development accompanied by advanced cell culture strategies, as suggested by Figure 1.
\section*{Discussion}
The creation of a mechanistic and predictive model is a serious and work-intensive endeavor that forces all participants to think deeper \cite{wolkenhauer2014model}. Ultimately, the reward is more aim-tailored research but also the discovery of hidden causalities, which would otherwise have rendered explorative research inconclusive or contradictory. Recent progress in devising experimental procedures for parameter determination has fueled the work of several computational groups. Conversely, certain phenomena can only be understood with computational methods, such as computational mechanics. Mathematical modeling of melanoma presents several specificities ranging from the high mutation load and cell plasticity to the oxygen-delivery uptake at the skin surface. Therefore,Nevertheless, most of the current models of melanoma are not yet sufficiently adapted to the requirements in biology and medicine. The recurring problems in almost all reviewed research can be expressed in four challenges became apparent. \\and are discussed accordingly.
\subsection{First challenge: tumor heterogenity}
The first challenge is the biochemical heterogeneity. The high mutation load, signaling network plasticity, and cell line heterogeneity makes the fitting of mechanistic \ac{ode} systems or straightforward network inference from patients' biopsies difficult. Instead, most studies focus on

well-characterized cell line collections to carefully extract specific regulatory network motifs with multivariate statistics. The cell line-specific models are suitable for modeling and understanding drug testing. Notable works used systems biology to investigate the impact of new compounds such as TRAIL \cite{passante2013systems, del2018systemic}, while others focus on identifying potential targets by perturbating the biological system with several kinase inhibitors \cite{bernardo2014data,fallahi2015systematic}. ~~\\~~

\subsection{Second challenge: melanoma type specifity}

The second challenge is melanoma type~~-~~ specific modeling. Melanocytic tumors occur in various forms at different locations and are based on different etiologies \cite{elder2018classification}. ~~However~~A few important types are lentigo maligna melanoma, superficial spreading melanoma, and acral lentiginous melanoma. Nonetheless, computational papers often refer ~~often~~ to a general term of melanoma, albeit each melanoma type can substantially differ in treatment, environment, and growth pattern. In computational biology, mechanistic links between growth patterns ~~are rarely linked to~~ and melanoma-type specific biochemical markers could prospectively find the same importance as in pathology \cite{shain2016melanocytes, elder2018classification~~}, depend only on one or two~~}. In contrast to constructing models around a few abstract mathematical parameters~~,~~ and ~~authors~~ retrospectively allocate histopathological sections to ~~their~~a given simulation outcome, modelers might emphasize the pathological causality and relevant biochemical root-causes leading to a melanoma-type specific growth outcome. A deeper examination of cancer pathology, anatomy, and physiology might also prevent unjustified assumptions. Some authors set initial lesions at positions, where they rarely project from such as the epidermal stratum corneum, albeit the stratum basale is often the location of initial lesions \cite{elder2018classification}. The unique oxygen patterns in skin \cite{stucker2002cutaneous, wang2003oxygen}, the tendency of melanocytes to proliferate better in mild hypoxic conditions \cite{horikoshi1990effects}, the strong oxygen consumption of melanoma cells \cite{hall2013dysfunctional}, or the importance of driver mutations in this highly mutated cancer type \cite{akbani2015genomic, loftus2018next} ~~found a rare propagation in the modeling community~~are further factors, which ~~too often copies~~might find more consideration by modelers of melanoma. Not all concepts, model structures, and parameters can be ingenuously taken from models of other cancer types. Future melanoma models might represent more melanoma type~~-~~ specific characteristics and parameters, whereby attention should also be drawn to the respective histopathology and the host tissue in which the simulated melanoma is thought to be simulated. Eventually, context and tissue-specific modeling of certain melanoma types is more insightful than generic cancer or melanoma models~~. \\~~.

\subsection{Third challenge: complexity}

The third challenge is the appropriate level of complexity as neither very small and simple nor extensive models can deliver reliable predictions. Models that are as simple as possible are the gold standard in modeling, as perfectly shown by Kim or Picco \textit{et al.} \cite{kim2016phase, picco2017integrating}. However, if models neglect major effects, or the remaining model elements are too abstract to be interpreted, the result will be of little use. For example, careful work was performed to determine mutually dependent parameters of a cell colony \cite{haridas2017quantifying}. However, the impact of the mechanical environment on these parameter values \cite{ju2018role}, such as migration~~,~~ \cite{tozluouglu2013matrix}, exacerbates the transfer of these parameters to complex 3D models. On the contrary, especially large scale models which have been set-up \cite{pappalardo2016computational, wang2013multi} will benefit from sufficient and appropriate melanoma-specific data, to further increase the validity of their conclusions. The same can be observed in physical oncology. Mixture theory allows easier models and fewer parameters. Still, it is difficult to measure abstract parameters or to biologically interpret the equations as they pool too many biological sub-

systems to homogeneous entities. In contrast, Sciumé \textit{et al.} accurately differentiates between cells, fibrous compounds, and interstitial fluid. This makes experimental parameter determination easier and aligns better with medical and biological lines of thinking. However, the model requires many parameters, which must still be biologically characterized. Best interdisciplinary communication is reached with agent-based models, were cells are separately depicted. However, the computational demand for simulating individual cells is substantial. More experience is necessary to find the right level of complexity that can be of practical use and allow both computationally feasible and biologically sound models. \\
\subsection{Fourth challenge: correct data integration}
The fourth challenge is the accurate integration of evidence. Experimental fact and assumed fiction are difficult to distinguish in many publications, and it is of little use if an extensive biological section is written independently of the computational part but at the end is nothardly reflected by the equations at the end \cite{ciarletta2010radial}. To enable scrutiny by melanoma experts and to facilitate evidence-based model extensions or improvements, it seems necessary that each element of a model structure is biologically explained, interpreted, and referenced only within the degree of factual implementation, even if this requires extensive supplemental information. The reasoning behind modeling decisions should be accountable. At least, the behavior of all system elements might be checked for plausibility as done in one work \cite{lee2015systems}. \\
Besides the verifiability of the model structure, parameters are very ambiguous in most papers, and only a few papers provided supplemental information about data extraction and conversion \cite{sun2016mathematical}. Unfortunately, many publications work with uncurated parameter lists, and interested readers are recommended to trace back parameter values to the primary source to judge the validity. We found that data indicated as melanoma-specific turned out to bewere based on other diseases and tissue origins such as glioblastoma or the adrenal gland, that. Other estimated or assumed parameters are referenced in subsequent papers as if they are experimentally determined values, that. Moreover, the context of experimental origin often do not fit the intended model context, and thator whole parameter sets are normalized in an original paper and then carried over several computational paper generations regardless of studied biology. In order to bring models closer to biological evidence, parameters should be referenced only to the original experimental publications, and information should be given on the experimental context and potential parameter conversions. \\
Most likely, not all required data will be available, but transparency on evidence is generally lacking, and it seems that readers have to trace back parameter values on their own to judge a computational work.. It remains to be debated to which extend a model must contain melanoma-specific data to be considered a melanoma model or evenhow close a model must match medical evidence to be seen as a valuable contribution to melanoma research. \\
\subsection{Lack of interdisciplinary is the root cause}
The four challenges reflect the greatestmost persistent problem of melanoma-specific modeling: interdisciplinarity. Computational models require close collaboration between experimental, clinical, and computational scientists in an iterative procedure. Modeling generates hypotheses, which can be tested \textit{in vitro}, and experimental results inform the design of better models and allow the falsification of theories \cite{howard2014quantitative}. However, a general problem in the interdisciplinary work in biology and medicine is that the more demanding the necessary mathematical and physical framework becomes, the more disconnected it becomes from the experimental and theoretical knowledge in biology, medicine, and pharmacology. On the one hand, computational groups cannot reproduce and test the diverse parameter sources in their labs, lacking the time and expertise to embrace the whole complexity of biological relationships and experimental methods. On the other hand, biologists and clinicians find it difficult to help, as the more developed

computational procedures are likewise difficult to comprehend. Therefore, better quality standards between and in both computational \cite{waltemath2016modeling} and biomedical research \cite{begley2015reproducibility} need to be developed and adopted. A more sophisticated way might be the stepwise model development accompanied by advanced cell culture strategies, as suggested by Figure 1. The gap between the different disciplines is not closed yet, which leads to conceptual problems in the models and inappropriate parameter choices. ~~\\~~
~~The creation of a mechanistic and predictive model is a serious and work-intensive endeavor that forces all participants to think deeper \cite{wolkenhauer2014model}. Ultimately, the reward is more aim-tailored research but also the discovery of hidden causalities, which would otherwise have rendered explorative research inconclusive or contradictory. Recent progress in devising experimental procedures for parameter determination has fueled the work of several computational groups. Conversely, certain phenomena can only be understood with computational methods, such as computational mechanics.~~

\section*{Conclusion}

Cancer is a highly complex, heterogeneous disease, characterized by a series of genetic, metabolic, and functional changes at the cellular and tissue level \cite{hanahan2011hallmarks}. Melanoma-specific dynamics along tumor progression stages in both plasticity \cite{weder2014increased} and genetics \cite{shain2015genetic} highlight the need for integrative models to better understand disease mechanisms of melanoma~~ better~~. The model-building community works across different scales and comprises studies centered on signaling pathways and gene regulation \cite{werner2014cancer}, metabolism \cite{antoniewicz2015methods, pacheco2018fastcore}, epithelial tissue mechanics \cite{brodland2015computational}, tumor physiology \cite{cristini2017introduction}, or the immune system \cite{konstorum2017addressing}. Four challenges for computational melanoma models have been discussed:

\begin{itemize}
\item Melanoma heterogeneity,
\item Melanoma-type specificity,
\item The balance between simplicity and thoroughness, and
\item Melanoma data integration and evidence.
\end{itemize}

Consequently, interdisciplinarity and clinical relevance remain a bottleneck if it comes to the practical use of melanoma-specific systems biology and physical oncology models. However, if all disciplines improve interdisciplinary collaboration, the future promises us an unmatched insight.
